# Integrated In Silico and In Vitro Study of Copper Nanocatalyzed Carbonyl‐Functionalized Triazoles—Inducing S Phase Cell Cycle Arrest and Apoptosis in MCF‐7

**DOI:** 10.1002/open.202500543

**Published:** 2026-01-16

**Authors:** Joydip Mondal, Tiasha Dasgupta, Chitluri Kiran Kumar, Prasanth Babu Nandagopal, Sadananda Mal, Sourav Paul, Aishwarya S, Chayan Pandya, Isaac Arnold Emerson, Venkatraman Manickam, Akella Sivaramakrishna

**Affiliations:** ^1^ School of Advanced Sciences Department of Chemistry Vellore Institute of Technology (VIT) Vellore Tamil Nadu India; ^2^ School of Biosciences and Technology Department of Bio‐Medical Sciences Vellore Institute of Technology (VIT) Vellore Tamil Nadu India

**Keywords:** apoptosis, cell cycle arrest, CuO‐NP, glutathione, green chemistry, lipid peroxidation, mitochondrial membrane potential, reactive oxygen species

## Abstract

The demand for novel, selective anticancer agents, driven by drug resistance and systemic toxicity of current treatments, underscores the importance of targeted drug discovery. Present research involved cytotoxic screening of a series of synthesized copper nanocatalyzed carbonyl‐functionalized triazoles (**3a** **–** **p**), where **3i** and **3j** have shown highest selectivity index (SI) scores of 2.30 and 4.44, respectively. Computational validation of the lead compounds demonstrated specific interaction with BCL2‐associated X protein (BAX) and BCL2, characterized by strong binding affinities ranging between −6.73 and −7.70 kcal/mol. Corresponding protein–ligand complexes demonstrated robust conformational stability throughout their 100 ns of molecular dynamics simulation. Subsequent in vitro validation using MCF‐7 cells firmly corroborated the in silico findings, by revealing significant upregulation of BAX (*p *< 0.001) and downregulation of BCL2 (*p *< 0.001). Compound induced cellular stress, elevated the ROS‐producing cell population up to 40%. Resulting cellular oxidative stress, rapidly depleted the glutathione reserves up to 50% (*p* < 0.001), consequently compromising the mitochondrial membrane potential leading to mitochondrial dysfunction. Furthermore, the compound induced S‐phase cell cycle arrest (upto 51.5%), played a pivotal role in promoting apoptosis by activating DNA damage response pathways. In conclusion, this study has successfully identified two lead compounds (**3i** & **3j**) that modulate multiple converging oncogenic pathways, providing compelling preclinical candidates for targeted management of breast cancer.

## Graphical abstract

A targeted drug discovery pipeline identified novel copper‐nanocatalyzed carbonyl‐functionalized triazoles as potent breast cancer therapeutics (selectivity index up to 4.44). Lead compounds (**3i**, **3j**) induced apoptosis in breast cancer cells (MCF‐7) by modulating BAX/BCL2, causing reactive oxygen species (ROS)‐mediated glutathione (GSH) depletion, mitochondrial membrane potential (MMP) compromise, and DNA damage response activation, thereby causing S‐phase cell cycle arrest.

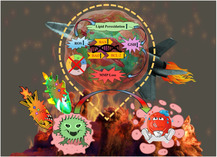

## 1 Introduction

Cancer remains a significant global health challenge, and while numerous anticancer drugs are commercially available, many suffer from limitations such as low specificity, safety concerns, severe side effects, and resistance issues. This underscores the urgent need to develop safer and more targeted anticancer treatments. Interestingly, over 85% of physiologically active pharmaceutical compounds incorporate heterocyclic structures or at least one heteroatom, with nitrogen‐containing heterocycles being the most prevalent framework in drug design [1].

Apoptosis, a crucial physiological mechanism of programmed cell death, plays a fundamental role in embryonic development, immune system homeostasis, and the cellular response to DNA damage [2]. This process is designed to proceed in a controlled manner, preventing the leakage of intracellular components and thereby avoiding the induction of an inflammatory reaction. However, any disruption in apoptosis regulation can have profound consequences, particularly in the context of cancer development [3]. Cancer cells, irrespective of their origin or type, universally exhibit key malignant traits, including angiogenesis, uncontrolled proliferation, and evasion of apoptosis. Apoptosis serves a crucial role in cancer prevention by eliminating damaged or aberrant cells. However, in cancer, the intrinsic apoptotic pathway is frequently suppressed, leading to unchecked cellular survival [4]. When apoptotic regulation fails, mutations can accumulate, enhancing tumor invasiveness, promoting angiogenesis, disrupting normal cell cycle control, and impairing differentiation. This extended survival enables cancer cells to persist and expand [5]. Various mechanisms allow cancer cells to circumvent apoptosis, such as inhibiting caspase activity or neutralizing apoptotic signals. The most common strategies for apoptosis evasion include the loss of proapoptotic proteins like BCL2‐associated X protein (BAX) and BCL2 antagonist/killer (BAK), along with the overexpression of antiapoptotic BCL‐2 proteins [6]. Although BCL‐2 is not classified as an oncogene, its mutations significantly accelerate tumor progression. Notably, over half of all malignancies, regardless of type, exhibit elevated BCL‐2 protein levels, reinforcing the role of apoptotic resistance in cancer pathogenesis [7]. One approach to cancer treatment involves controlling or halting the uncontrolled proliferation of cancer cells. Leveraging the cell's inherent apoptotic mechanism presents a highly effective strategy [8]. Among nonsurgical interventions, those that focus on apoptosis are particularly promising. Since evading apoptosis is a fundamental trait of cancer, independent of its origin or type, therapies designed to restore apoptotic pathways hold potential for treating all forms of cancer [9]. This universality makes apoptosis‐targeted strategies compelling for the development of novel anticancer treatments. A therapeutic approach capable of selectively activating apoptotic pathways could pave the way for more broadly applicable cancer treatments [10]. Nitrogen containing bi‐heterocycles play a crucial role in medicinal chemistry, drug design, and biotechnology. Their structural complexity allows for interaction with multiple biological targets, making them essential in cancer therapy, infectious disease treatment, CNS drugs, and imaging agents [11]. There is a pressing demand for the discovery of new synthetic methodology to access bio active heterocyclic molecules because a slight alteration in the electronic and steric properties of distant substituents within a molecule can influence its structural and binding characteristics when interacting with biological substrates [12, 13]. Moreover, presence of carbonyl group in core moiety is indispensable in biological systems, influencing protein structure, genetic material stability, metabolism, enzymatic reactions, and drug action [14]. Its versatility in hydrogen bonding and reactivity makes it a key component in life processes and pharmaceutical applications.

**FIGURE 1:**
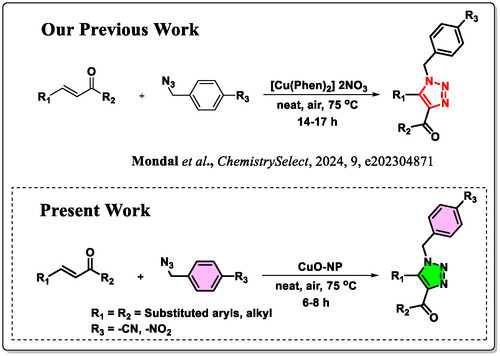
CuO nanoparticle based regioselective synthesis of trisubstituted triazole.

In recent years, numerous synthetic approaches for the preparation of 1,2,3‐triazoles have been explored, often involving the use of external additives, expensive catalyst, and prolong reaction time to facilitate the reaction between chalcones and azides [15, 16, 17–18]. In comparison to previously reported methodologies, we have developed a novel and efficient copper nanoparticle mediated synthesis of disubstituted triazoles using chalcones and azides under solvent‐free conditions, this method requires less time compared to previously reported methodologies, and the CuO nanoparticles are reusable for up to five cycles (Figure 1). This newly developed approach stands out due to its simplicity, cost‐effectiveness, and ecofriendly nature. Unlike conventional methods that require additional reagents or harsh reaction conditions, our strategy eliminates the need for solvents and external additive, making it a more sustainable and environmentally viable alternative. The combination of enhanced accessibility, affordability, and reduced environmental impact makes this method a promising advancement in triazole synthesis. In this research work we took an approach of in silico and in vitro to study the potentiality of synthesized carbonyl functionalized N heterocycle to induce apoptosis in MCF‐7 breast cancer cells.

## 2 Experimental Section

### 2.1 Materials and Instruments

Substituted aldehydes, substituted acetophenone, various benzyl bromides, and common organic solvents (*n*‐hexane, ethyl acetate, methanol, ethanol, *N*,*N*‐dimethyl formamide, dimethyl sulfoxide, etc.) were purchased from Sigma‐Aldrich and AVRA chemicals (INDIA). 0.25 mm silica gel‐coated analytical thin‐layer chromatography (TLC) was utilized (Kieselgel 60 F254 plates). Bruker DRX400 spectrometer was utilized for characterization of ^1^H NMR (400 MHz) and ^13^C NMR (100 MHz) and were designated in Hertz (Hz) and parts per million (ppm), respectively, using TMS as internal standard solvent. Spectrum multiplicity was assigned as s (singlet), d (doublet), dd (doublet of doublet), dt (doublet of triplet), and m (multiplet). FT‐IR spectra were obtained from a IRAffinity (SHIMADZU) and Nicolet iS50 (Thermo Scientific) spectrometers on a KBr disc mode. UV−visible absorption spectra were recorded using JASCO V‐730 spectrophotometer at room temperature in 10% DMSO–H_2_O solution. A Hitachi F‐7000FL spectrophotometer was employed to determine emission fluorescence spectra by excitation at their respective absorption maxima. With the use of a Q‐T of Micro mass spectrometer, HRMS measurements were recorded. Cell culture essentials like Dulbecco's Modified Eagle's Medium (DMEM), fetal bovine serum (FBS), MTT, antibiotics, trypsin, and dimethyl sulfoxide (DMSO) were purchased from Hi Media (India). RT‐PCR reagents, including the SYBR Green kit and cDNA synthesis kit, were sourced from TaKaRa (Japan). Chemicals such as DAPI, Rhodamine 123, TRIzol were purchased from Sigma‐Aldrich (India) and cell culture plastics were acquired from Tarson, India.

### 2.2 Synthesis and Characterization

Copper nanoparticles were synthesized according to this previously reported protocol [19]. A 100 mL aqueous solution of 0.1 M Cu(CH_3_COO)_2_·H_2_O was stirred at 900 rpm, and NaOH was added (1:2 molar ratio) until pH 12. The solution turned from blue to black after 4 h at 80°C, indicating precipitate formation. Stirring speed and temperature were maintained throughout. The product was collected, washed thrice with distilled water via centrifugation (8000 rpm, 20 min each), and dried at 100°C for 3 h before characterization.

### 2.3 Drug‐Likeness Prediction

The druggability of the selected compounds (**3i** and **3j**) was evaluated using LRO5, which considers key factors such as topological polar surface area (TPSA), lipophilicity (MiLogP), molecular weight (MW), and the number of hydrogen bond acceptors (nON) and donors (nONH) (Table 1). This drug‐likeness study aims to determine if a drug meets the five criteria outlined in Lipinski's Rule of Five (LRO5) to confirm that it has the necessary physical and chemical characteristics for use as a pharmaceutical, especially for oral use in humans. The compounds identified through MTT assay screening were assessed for adherence to LRO5. This evaluation was conducted using the web application “Molinspiration Cheminformatics” (https://www.molinspiration.com). To input and prepare SMILES structures in the program, only the SMILES representations of the molecules are required, with no need for knowledge about the catalytic area or interaction mechanisms.

**TABLE 1:** Milog P partition coefficient ≤ 5, TPSA ≤ 140 Å2, MW ≤ 500, H‐B A hydrogens acceptor ≤ 10, H‐B D hydrogen donor ≤ 5, nA number of atoms < 70, nV number of violations, nR number of rotatable bonds ≤ 10.

Entry	MW	MiLogP	TPSA	H‐B‐A	H‐B‐D	nA	nV	nR	Volume
**3i**	390.08	2.768	90.92	7	0	24	0	6	377.47
**3j**	402.11	2.624	90.92	7	0	25	0	6	396.99

### 2.4 Density Functional Theory Analysis (DFT)

Molecular electrostatic potential (MEP), was employed to assess the stability and reactivity of the molecule, thereby validating the reactive components for electrophilic and nucleophilic centers. The GaussView 6.0 software was utilized in this investigation to obtain the interactive visualization of the structures of compounds, whereas Gaussian 09 software was employed to conduct the computation. At the level of B3LYP/6‐311G (d,p), geometry optimization was carried out on the questioned compound utilizing the unconstrained DFT approach. MEP and Mulliken charge were then calculated, and the lowest unoccupied molecular orbital (LUMO) and the highest occupied molecular orbital (HOMO) were identified.

### 2.5 Molecular Docking and Molecular Dynamics (MD) Simulation

Molecular docking was performed using AutoDockVina to predict the binding affinity and interaction pattern between the target protein and the selected ligands. Protein structures for BAX and BCL2 were obtained from the Protein Data Bank with PDB ID: 8SRY, 6O0K, respectively and prepared by removing heteroatoms such as water molecules and ions. Ligands were prepared in SDF format, and nonpolar hydrogens were removed from the receptor files with partial charges added to carbon atoms. The docking process utilized the AutoDock Vina engine integrated within PyRx, with the binding site defined as a spherical region encompassing all protein atoms within a 15 Å radius of the active site. Default parameters were used for grid generation and docking calculations. The ligands **3i** was minimized with *E* = 527.49 and *E* = 516.73 for **3j**. Additionally Kollman charges (MK) were added to the BAX and BCL2, −81.973. The grid coordinates for BAX were center_x = 18.0461, center_y = 19.755, center_z = −7.2043, size_x = 49.0859989405, size_y = 40.8910991544, and size_z = 28.9634498405 and for BCL2 center_x = −3.7373, center_y = 1.7969, center_z = −11.3825, size_x = 37.3204466438, size_y = 34.9727012539, and size_z = 42.5011500168. Docking results were analyzed based on binding affinities and conformations, with the best‐scoring poses visualized using Biovia Discovery Studio.

Molecular dynamics (MD) simulations were conducted using GROMACS 2023.2 to evaluate the stability and dynamics of the docked complexes. Protein–ligand complexes were prepared by adding hydrogen atoms, assigning CHARMM force fields, and solvating the system in a cubic water box with TIP3P water molecules. The system was neutralized by adding counter ions. Energy minimization was performed to remove steric clashes, followed by equilibration in two phases: NVT (constant number of particles, volume, and temperature) and NPT (constant number of particles, pressure, and temperature) ensembles. Finally, production MD was run for 100 ns under periodic boundary conditions. Trajectories were analyzed for root mean square deviation (RMSD), root mean square fluctuation (RMSF), hydrogen bonding, solvent‐accessible surface area (SASA), and radius of gyration (Rg) calculations to assess complex stability. Table 2 summarizes this analysis.

**TABLE 2:** IC_50_ values and corresponding selectivity indices of the tested compounds against breast cancer cells (MCF‐7) and human embryonic kidney (HEK‐293) cells.

Compound^a^	**MCF‐7,** **μ** **M** b	**HEK‐293,** **μ** **M** c	Selectivity index
3a	14 ± 1.2	20 ± 2.23	1.42
3b	73 ± 0.7	90 ± 3.12	1.23
3c	3.5 ± 2.5	4.75 ± 1.16	1.34
3d	5 ± 0.3	6.5 ± 2.54	1.3
3e	61 ± 0.9	65.5 ± 2	1.07
3f	52.5 ±0.7	55 ± 3.12	1.04
3g	3.5 ± 1.4	4.25 ± 2.45	1.21
3h	12.5 ± 0.9	15 ± 1.12	1.2
**3i**	**6.5 ± 1.5**	**15 ± 1.54**	**2.30**
**3j**	**4.5 ± 0.1**	**20 ± 2.21**	**4.44**
3k	63.5 ± 1.1	70 ± 3.54	1.10
3l	12.5 ± 0.7	15 ± 1.94	1.2
3m	18 ± 1.4	20 ± 2.56	1.11
3n	20 ± 2.1	25 ± 2.78	1.25
3o	9.5 ± 0.1	12 ± 1.03	1.26
3p	25.5 ± 0.7	27 ± 4.12	1.05

a IC‐50 values are average ± SD of three individual experiments.

b Breast cancer cells.

c HEK human embryonic kidney cells.

### 2.6 Cell Culture Methods and Cytotoxicity Assessment

Toxicological evaluation was carried out on Breast cancer cells (MCF‐7) and Human Embryonic kidney cells (HEK‐293). Cells were procured from the National Centre for Cell Science (NCCS, Pune, India). The cells were grown in a supplementary media prepared by mixing 10% FBS and 1% Antibiotics in DMEM. They were grown in adherent culture flasks (25 mm^2^) and kept in an incubator that maintains humidified conditions like 37°C and 5% CO_2_ throughout. Cells were harvested from the confluent flasks and seeded onto a MTT plate, at a proportion of 1 × 10^4^ cells/well and incubated overnight. Post incubation, the cells were treated with ascending concentrations of the compound, dissolved thoroughly in 0.1% DMSO for a period of 24 h. Then, cells were subjected to MTT (5 mg/ml in PBS) treatment for 4 h in the dark. DMSO was used to solubilize the formazan crystals and Optical density values were recorded at 490 nm by employing a microplate reader (Bio‐Tek, USA). Selectivity Indices (SI) were calculated to assess the compound's differential toxicity between cancerous and normal cells [20]. A higher SI value (ideally SI > 1) indicates greater safety and therapeutic potential, as the compound is more toxic to the cancerous cells than to the healthy cells.

$$\mathrm{Selectivity}\;\mathrm{Index}\left(\mathrm{SI}\right)=\frac{{\mathrm{IC}}_{50}\;\text{of non-cancerous cells}\;\left(\text{HEK-}293\right)}{{\mathrm{IC}}_{50}\;\text{of cancerous cells}\;\left(\text{MCF-7}\right)}$$

### 2.7 Intracellular Reactive Oxygen Species (ROS) Assessment by DCFH‐DA Staining and Flow Cytometry

The DCFH‐DA probe was utilized to measure the cellular shift toward an oxidative state, and while its nonspecific nature prevents the direct quantification of isolated H_2_O_2_, the resulting fluorescence data reliably represents the global cellular oxidative stress burden. MCF‐7 cells seeded in a multiwell plate were subjected to overnight incubation to achieve adherence and desired growth. Subsequently, they were treated with **3i** and **3j** for 24 h. The morrow cells were washed twice with PBS and exposed to a fluorogenic dye, DCFH‐DA for a period of 15 min. EVOS M5000 Imaging System was leveraged to record the ROS production with excitation and emission wavelengths of 530 and 480 nm, respectively [21]. Culture and treatment procedure were followed same as above for quantification of produced ROS. Thereafter, the cells were collected by trypsinization and centrifuged. In the absence of light, cells were administered with 20 μM DCFH‐DA dye for 30 min. CytoFLEX (Beckman Coulter) and CytExpert software was utilized to measure the levels of ROS and analyze the same, respectively [22].

### 2.8 Investigation of Mitochondrial Membrane Potential (MMP) with Rhodamine‐123 Dye

A density of 1 × 10^5^ cells per well was seeded in six‐well plates and incubated overnight under humidified conditions. Cells were treated according to the protocol, washed twice with PBS, and incubated with 5 µg/ml Rhodamine 123 for 20 min. Flow cytometry (CytoFLEX, Beckman Coulter, USA) was used to determine mitochondrial membrane potential (MMP) at an excitation wavelength of 488 nm. Cisplatin served as a positive control in this experiment [23].

### 2.9 AO/EtBr Staining for Apoptosis and DAPI Staining for DNA Fragmentation

Cells underwent identical treatment and washing procedure as outlined previously. Subsequently they were stained and counter stained with Acridine orange and Ethidium bromide for 15 and 10 min, respectively, in the absence of light with a PBS wash in between them. Fluorescence microscope was used to distinguish and record the viability of the cells [24]. DNA fragmentation has been an indicator of apoptosis. Hence, it was necessary to study the fragmentation status of the DNA. Cellular nuclear architecture was imaged through DAPI (4′, 6‐diamidino‐2‐phenylindole) (Sigma‐Aldrich, India) Staining. Aforementioned protocol was followed to culture and treat the cells. Upon washing the cells twice with PBS, they were dark‐incubated with DAPI (1 µg/ml) for 10 min. Fluorescence microscope (EVOS M5000 Imaging System) was utilized to image the nucleus with excitation and emission wavelengths of 358 and 460 nm, respectively. Cisplatin was used as Positive control [25].

### 2.10 Isolation of RNA and RT‐PCR

Following treatment as per the protocol, cells were harvested, and RNA was isolated using TRIzol. RNA concentration was determined using a NanoDrop spectrophotometer (BioSpectrometer, Eppendorf). cDNA synthesis was performed using the PrimeScript RT Reagent Kit (Perfect Real Time). Real‐Time PCR amplification was carried out with specific primers (Table S1) and SYBR Premix Ex Taq II (Tli RNase Plus) master mix. Glyceraldehyde‐3‐phosphate dehydrogenase (GAPDH) was employed for normalization. Gene expression levels were quantified using the 2^−ΔΔ*Ct*^ method, and fold change relative to the control was reported.

### 2.11 Protein Quantification

Treated MCF‐7 cells were collected and lysed to prepare cell lysate. Target protein levels has to be normalized with total protein expression of the cells. Hence, total protein concentration were quantified using BCA assay kit following manufacturer's protocol.

### 2.12 Glutathione Synthase (GSH) Assay

Glutathione being a key antioxidant, plays a major role in guarding the cells from oxidative damage by neutralizing the ROS and free radicals. Thus, glutathione (GSH) was quantified by preparing a reaction mixture of phosphate buffer (pH 7.4, 50 mM), 5,5‐dithiobis‐(2‐nitrobenzoic acid (DTNB) (60 µM) and sample supernatant in the ratio of 3:7:1, respectively. Microplate reader (Bio‐Tek, USA) was used to record the absorbance at 412 nm. Standard curve was used calculate the reduced glutathione and data was expressed in µM/mg of protein.

### 2.13 Lipid Peroxidation (LPO) Assay

Malondialdehyde (MDA), a product of lipid peroxidation and a key indicator of oxidative stress, was quantified. A 1:1 mixture of supernatant and 5% trichloroacetic acid (TCA) was incubated on ice for 10 min. Following the addition of thiobarbituric acid (TBA) (w/v), the reaction mixture was centrifuged at 4°C for 10 min at 5000 rpm. Finally, sample mixture was kept to 95°C water bath for 15 min and allowed to cool down to RT. Absorbance was measured at 535 nm in a ELISA plate reader (Bio‐Tek, USA).

### 2.14 Statistical Analysis

Statistical analyses were performed using GraphPad Prism 8.0 software. All experiments were conducted in triplicate as a minimum, and data are presented as mean ± standard deviation (SD). Group differences were assessed via one‐way analysis of variance (ANOVA) followed by Bonferroni posthoc multiple comparisons. A significance level of *p* < 0.05 was employed for all statistical tests. Statistically significant differences between groups are indicated by asterisks (*) where ***p < 0.001, **p < 0.05, and *p < 0.01.

## 3 Results and Discussion

### 3.1 Structural Characterization of Synthesized Nanoparticles

**FIGURE 2:**
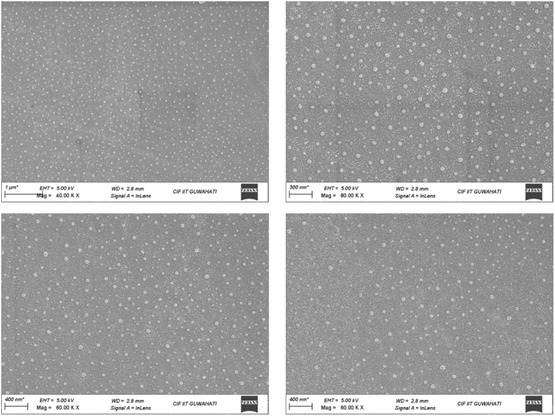
SEM images of CuO nanoparticle.

**FIGURE 3:**
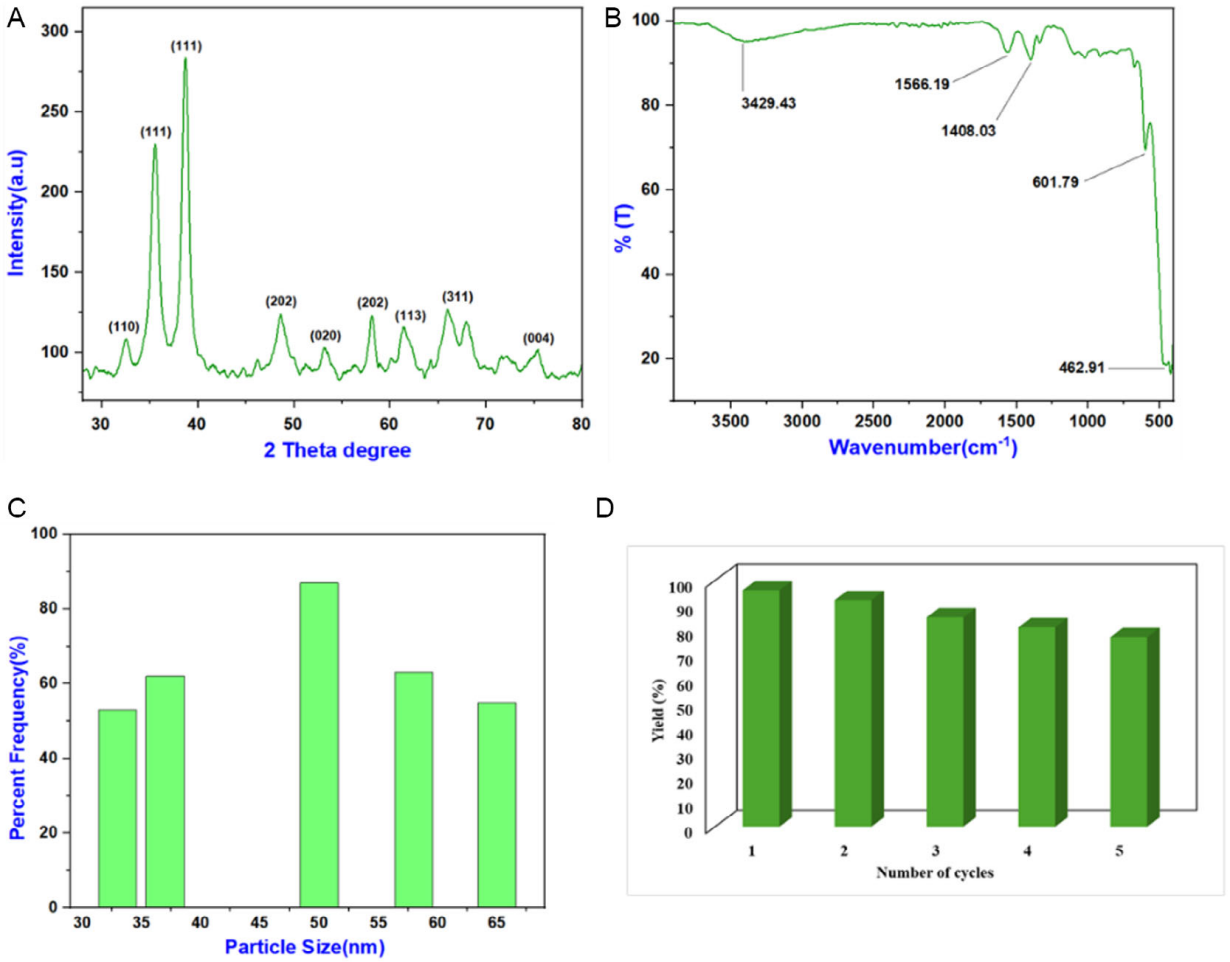
(A) XRD pattern, (B) IR spectrum of synthesized CuO NP, (C) particle size distribution of CuO nanoparticles, and (D) reusability of CuO nanoparticles catalyst in synthesis of triazole derivatives.

The SEM method was employed to gain deeper insight into the structure of copper oxide nanostructures. Monodispersed nanoparticles with a relatively uniform morphology were observed (Figure 2). The average diameter of the nanostructures was determined to be ≈33, 37, 50, 58, and 65 nm based on the distribution in the histogram (Figure 3C). Powder XRD analysis was conducted after calcination to examine the crystal structure and crystallinity of the material, confirming that the CuO nanoparticles exhibited a monoclinic crystal system (Figure 3A). In FT‐IR analysis, peaks at 462.91 and 601.79 cm^−1^ confirmed the formation of Cu—O bonds, further supporting their monoclinic structure (Figure 3B). The catalyst's recyclability was evaluated through multiple reactions. After the reaction, it was separated by filtration using ethyl acetate, washed, and reused. The catalyst remained efficient for up to five cycles (Figure 3B) but gradually declined due to the loss of material during the recovery process.

A series of substituted chalcones were initially reacted with substituted benzyl azide in the presence of copper oxide nanoparticles (CuO NPs) as a catalyst, using ethanol (EtOH) as the solvent at 80°C for 12 h (Table 3, entry 1). This reaction resulted in an 81% yield of the desired cycloaddition product. However, when ethanol was replaced with methanol (MeOH), a decrease in reactivity was observed, leading to a slightly lower yield of 76% (Table 3, entry 2). To further optimize the solvent conditions for this cycloaddition reaction, various polar solvents were tested (Table 3, entry 4 and entry 5). However, these alternative solvents only provided moderate yields compared to ethanol, indicating that ethanol remained a more effective medium for the reaction. Additionally, to examine the impact of temperature, the reaction was performed in ethylene glycol, a high‐boiling‐point solvent, but the yield dropped slightly to 74% (Table 3, entry 8), suggesting that excessively high temperatures did not significantly enhance the reaction efficiency. All reactions were conducted using a copper oxide‐based nanocatalyst to facilitate the transformation. Interestingly, when the reaction was carried out under solvent‐free conditions, a remarkable improvement in efficiency was observed. In the absence of a solvent, the reaction time was significantly reduced to just 7 h, and the yield increased dramatically to 96% (Table 3, entry 9). This substantial enhancement in product formation can be attributed to the greater intermolecular interactions between reactants in the absence of solvent molecules, which may otherwise interfere with the reaction process. Therefore, solvent‐free conditions were identified as the most favorable for maximizing yield and reaction efficiency in this transformation. Additionally, various copper catalysts were tested under solvent‐free conditions, but their efficiency was lower compared to the copper oxide nanocatalyst. CuO NPs demonstrate superior reactivity in organic synthesis. This enhanced performance is attributed to their high surface area to volume ratio, which provides more active sites and promotes efficient interactions with chalcone and azide, resulting in accelerated reaction rates and higher product yield.

**TABLE 3:** Optimization of reaction conditions for the facile synthesis of triazole derivatives by using copper oxide nanocatalyst.^a^

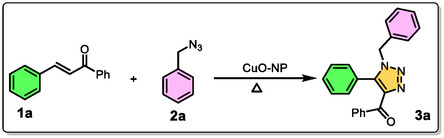				
Entry	Catalyst	Solvent	Time, h	Yield, %^b^
1	CuO‐NP	EtOH	12	81
2	CuO‐NP	MeOH	12	76
3	CuO‐NP	IPA	12	71
4	CuO‐NP	DMF	12	53
5	CuO‐NP	DMSO	12	59
6	CuO‐NP	THF	12	61
7	CuO‐NP	CH_3_CN	12	63
8	CuO‐NP	Ethylene glycol	12	74
9	**CuO‐NP**	**Neat**	**7**	**96**
10	Cu(OTf)_2_	Neat	7	83
11	CuBr_2_	Neat	7	69
12	CuCl_2_	Neat	7	61
13	Cu(OAc)_2_	Neat	7	71

a Reaction was carried out with 3 eq (mmol) of azide **2a** and 1 eq (mmol) of chalcone **1a** in at 75°C for 6 h.

b Isolated yield of **3a**.

Using the optimized reaction conditions, 15 sets of derivatives were synthesized, incorporating electron‐withdrawing, electron‐donating, and heteroatom substituents (Figure 4). The presence of electron‐withdrawing groups enhanced cycloaddition reactivity compared to electron‐donating groups, while the inclusion of a naphthalene moiety reduced productivity due to steric interactions. Under solvent‐free conditions, the intermolecular interaction between chalcone and azide was stronger than in solvent‐mediated reactions, resulting in higher product yields. All synthesized compounds were characterized using ^1^H NMR, ^13^C NMR, IR, HRMS, and UPLC techniques and the characterization data are presented in Figures S1–S75.

**FIGURE 4:**
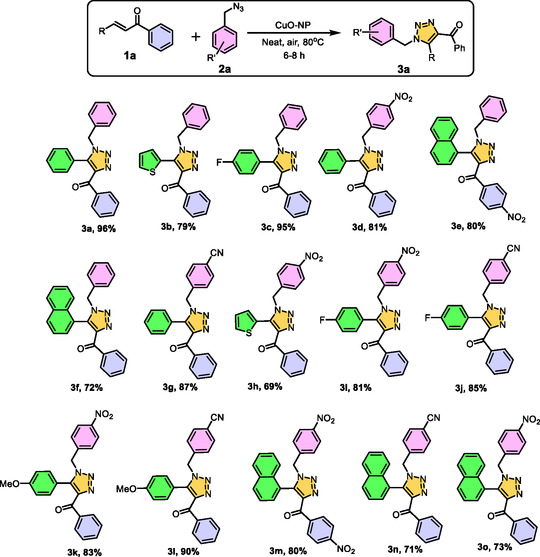
Substrate scope of chalcone and azide in presence of copper oxide nanocatalyst.

### 3.2 Lead Compounds Exhibited Favorable Drug‐Likeness

The molecular weight of both the compounds fell within the recommended limit (MW ≤ 500), which is crucial for effective distribution and absorption. Although larger molecular weights may present difficulties regarding absorption and distribution because of their increased size, **3i** and **3j** did not show notable challenges in this aspect.

### 3.3 Computational Prediction of Electronic Structure and Reactivity via Density Functional Theory (DFT)

**FIGURE 5:**
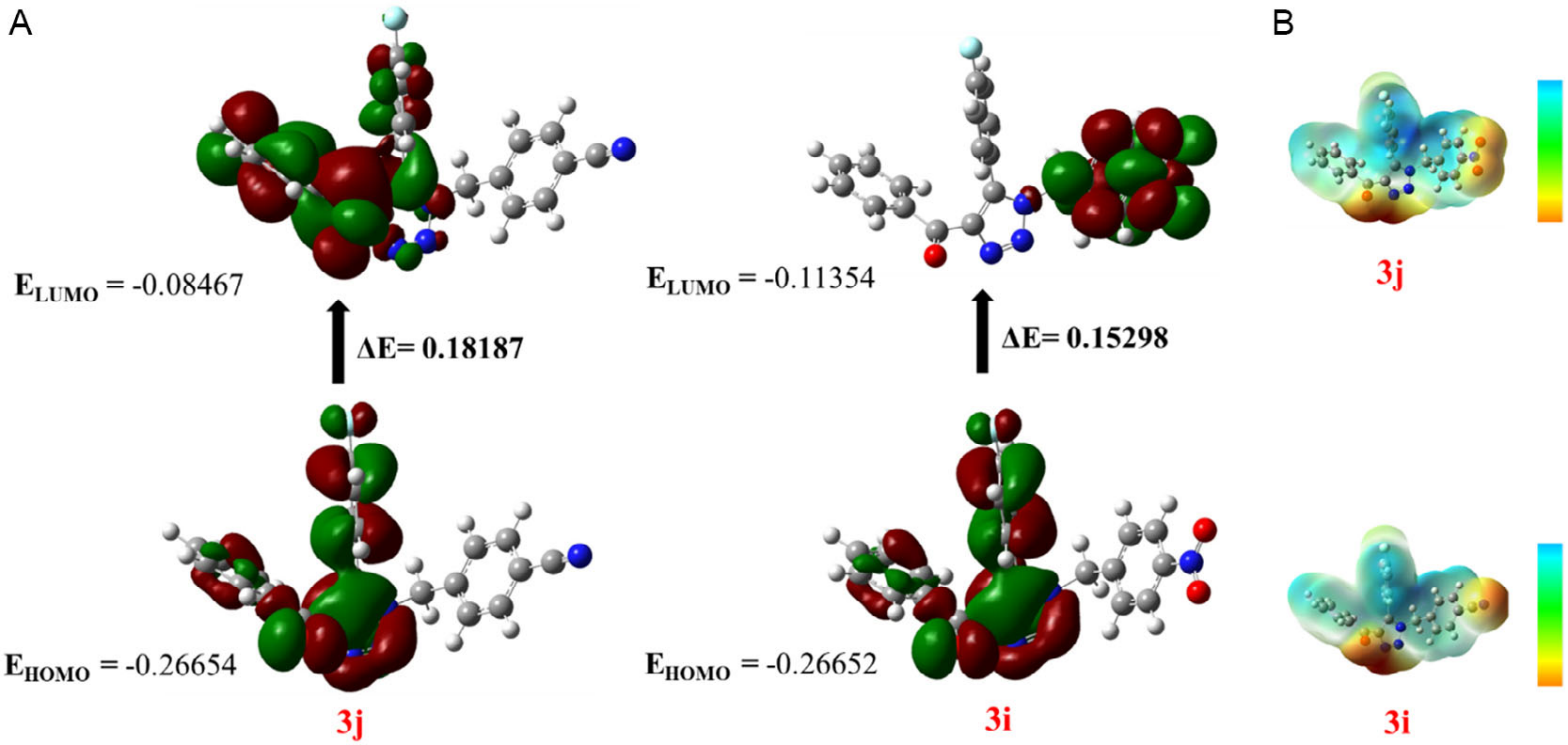
Energy of optimized structures (A) **3i** and **3j**, HOMO and LUMO of synthesized compounds **3i** and **3j**. The MEP diagram (B) of **3i** and **3j** was obtained from DFT calculation.

The electron density calculated using the density funtional theory (DFT) method with different basis sets (including B3LYP and 6‐311G*) and the bandgap value derived from the HOMO–LUMO analysis can indicate the likelihood of the molecule being reactive in biological contexts. The molecular energy of the optimized compounds (compounds **3i** and **3j**) is presented in Table S3. Notably, compounds **3i** (−1392.13011061 a.u.) and **3j** (−1279.83346467 a.u.) exhibit the same molecular energy. An optimized shape is deemed more stable when its ground state energy value is lower. According to DFT, the electron density surrounding a molecule's nucleus is critical in determining its overall energy. Furthermore, total energy comprises five components: correlation energy, electron‐to‐electron coulomb repulsion, kinetic energy, potential energy, and exchange energy attributed to the Pauli Exclusion Principle. The stability of a molecule increases as its energy decreases. The two primary frontier molecular orbitals (FMOs) are the HOMO and the LUMO as illustrated in Figure 5. A key factor in evaluating anti‐cancer capabilities is the energy difference between HOMO and LUMO. Electrons tend to be accepted by the half‐filled LUMO and donated by the fully occupied HOMO. A reduced HOMO–LUMO bandgap, which may allow for high polarizability, could signify favorable characteristics for anti‐diabetic candidates. According to Figure 5. Compound **3i** exhibits the most promising anti‐cancer effects, having the lowest energy band gap value of 0.15298 a.u. On a MEP, different colors represent the positive, negative, and neutral electrostatic potentials on a molecule's surface. This plot serves as a valuable tool for identifying sites of electrophilic and nucleophilic attack, as well as hydrogen‐bonding interactions. The region of minimum MEP, where negative charge density is concentrated in an isolated molecule, aids in predicting and understanding the positioning and directionality of electrophilic activities. The topography of the 3D electrical distribution reveals minor variations in charge density. The MEPs of compounds **3i** and **3j**, with optimized structures, are shown in three dimensions in Figure 5. The blue regions indicating low electron density may serve as active sites for nucleophilic attack, while the red regions representing high electron density are potential sites for electrophilic approach. Significant red clouds are evident around each oxygen atom in the structure, suggesting that they may constitute a crucial part of the hydrophobic docking component, where hydrogen bonds function as the primary interaction mechanism between the ligand and the target protein.

### 3.4 Molecular Docking Reveals Favorable Binding Affinity of Compounds 3i and 3j with BAX and BCL2

**FIGURE 6:**
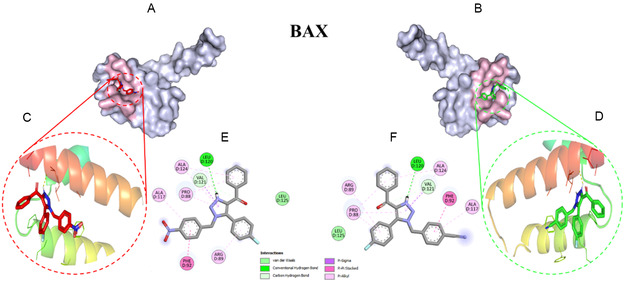
Auto Dock vina docking analysis between compound **3i** (red), **3j** (green) with BAX. (A,B) Surface representation of binding pocked; (C,D) 3D interaction in cartoon representation; and (E,F) 2D representation of the interaction using Discovery Studio.

**FIGURE 7:**
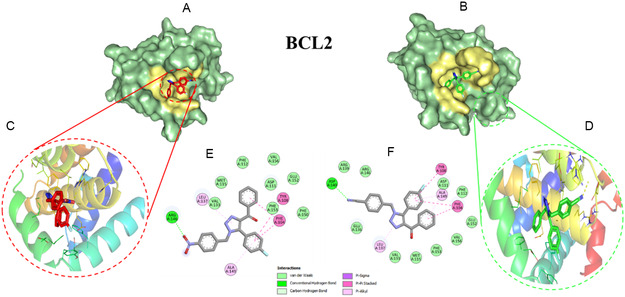
Auto Dock vina docking analysis between compound **3i** (red), **3j** (green) with BCL2. (A,B) Surface representation of binding pocked; (C,D) 3D interaction in cartoon representation; and (E,F) 2D representation of the interaction using Discovery Studio.

The docking score for **3i** and **3j** with BAX was determined to be −7.3 and −7.7 kcal/mol (Figure 6A–F), whereas binding energy of **3i** and **3j** with BCL2 are −6.73 and −7.20 kcal/mol, respectively (Figure 7A–F). The ligands were found to be stably positioned within the active site of the proteins, forming multiple non‐covalent interactions that contribute to its binding stability. The docking analysis of **3i** and **3j** with BAX and BCL2 revealed key interactions contributing to ligand stability and binding affinity. In the BAX complex, a hydrogen bond with Leu120 was identified as a crucial stabilizing interaction, alongside hydrophobic interactions (Table S2) that further enhanced ligand affinity. Additionally, π–π stacking interactions involving Phe92 played a significant role in complex stabilization. Similarly, in the BCL2 complex, hydrogen bonds were observed with Arg146 and Asp140, contributing to ligand stabilization within the binding pocket. Hydrophobic interactions further reinforced binding affinity, while π–π stacking interactions involving Phe104 and Tyr108 played a crucial role in stabilizing the complex. These findings highlight the structural basis for the ligand‐protein interactions, emphasizing the role of hydrogen bonding, hydrophobic interactions, and aromatic stacking in the stabilization of **3i** and **3j** complexes with BAX and BCL2. To further confirm the stability and dynamic behavior of the docked complex, a 100 ns MD simulation was performed, and key parameters such as RMSD, RMSF, Rg, SASA, and hydrogen bonds were analyzed (Table 4).

**TABLE 4:** A comparative evaluation of molecular dynamics simulation parameters‐including RMSD, RMSF, SASA, radius of gyration (Rg), and hydrogen bond interactions for both native and ligand‐bound (**3i** and **3j**) forms of BCL2 and BAX proteins.

Sample	Average. RMSD, nm	Average. RMSF, nm	Average. SASA, nm^2^	Average. Rg, nm^2^	Total. Hb
**Native BCL2**	0.15	0.11	83.09	1.46	—
**3i_BCL2**	0.51	0.11	82.47	1.47	4
**3j_BCL2**	0.27	0.11	82.22	1.47	3
**Native BAX**	0.32	0.19	62.36	1.45	—
**3i_BAX**	6.97	0.28	64.57	1.47	3
**3j_BAX**	6.19	0.34	65.50	1.45	5

### 3.5 Molecular Dynamics Simulation Analysis

#### 3.5.1 Root Mean Square Deviation (RMSD)

##### 3.5.1.1 3i and 3j Complex with BCL2

**FIGURE 8:**
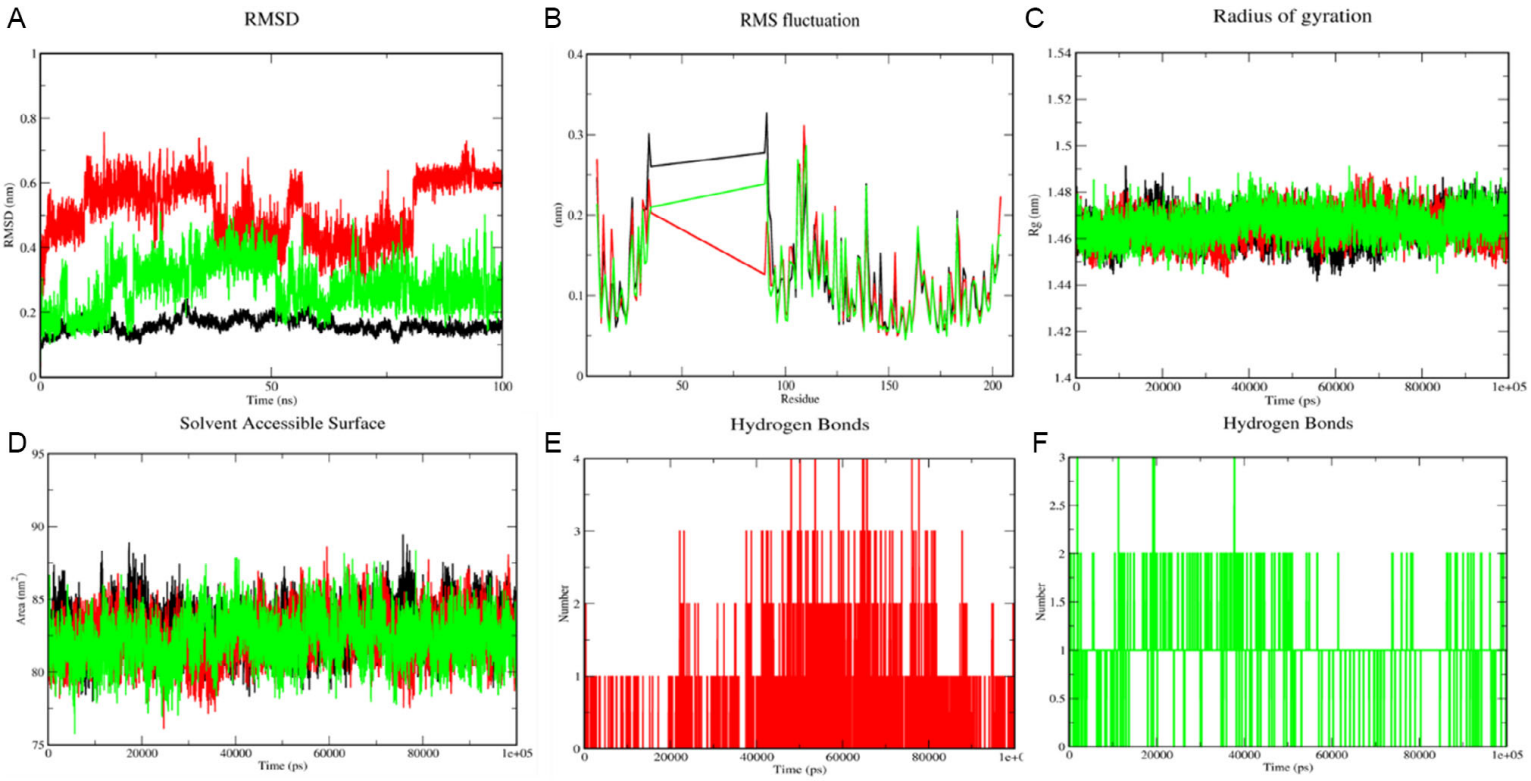
Molecular dynamic simulation analysis for the complex **3i_BCL2** (red), **3j_BCL2** (green), and native BCL2. (A) RMSD, (B) RMSF, (C) Rg, (D) SASA, and (E,F) Hydrogen bond analysis is depicted, respectively.

The RMSD analysis of the backbone atoms of the protein–ligand complexes was conducted to assess their structural stability over the course of the simulation. As shown in Figure 8A, the RMSD of complexes **3i** and **3j** with **BCL2** exhibited initial fluctuations during the first 12 ns, indicating system equilibration, with **3j** displaying lower fluctuations than **3i** by 0.20 nm. After this phase, minimal fluctuations were observed in **3i** and **3j** compounds until 78 ns and 50 ns, respectively. Complex **3i** reached a stable conformation from 80 ns, whereas complex **3j** stabilized earlier at 63 ns, attaining a lower average RMSD (0.27 nm) compared to **3i** (0.51 nm).

##### 3.5.1.2 3i and 3j Complex with BAX

**FIGURE 9:**
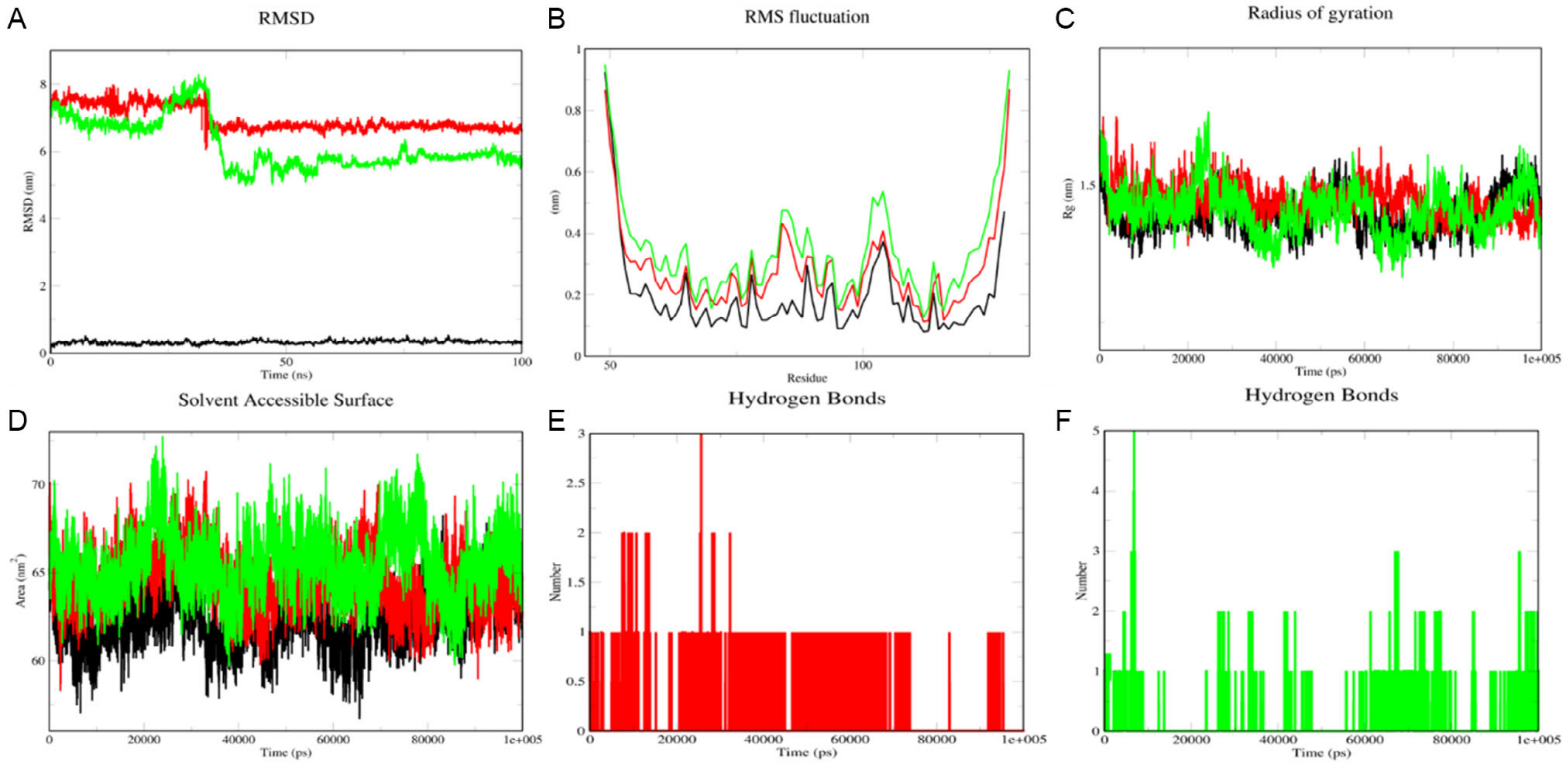
Molecular dynamic simulation analysis for the complex **3i_BAX** (red), **3j_BAX** (green), and native BAX. (A) RMSD, (B) RMSF, (C) Rg, (D) SASA, and (E,F) Hydrogen bond analysis is depicted, respectively.

Similarly, as shown in Figure 9A, the RMSD of complexes **3i** and **3j** with BAX exhibited initial fluctuations during the first ∼35 ns, indicating system equilibration. During this phase, **3j** displayed lower fluctuations than **3i** until ∼25 ns, followed by a gradual increase up to ∼35 ns, reaching a maximum peak of 8.3 nm. After this, minimal fluctuations were observed in the **3j** complex until ∼60 ns, after which it attained a stable conformation through the simulation, whereas **3i** stabilized earlier at ∼35 ns and remained stable throughout. Compound **3j** exhibited a lower average RMSD (6.19 nm) compared to **3i** (6.97 nm), further indicating its relatively stable binding.

#### 3.5.2 Root Mean Square Fluctuation (RMSF)

##### 3.5.2.1 3i and 3j Complex with BCL2

The RMSF analysis was performed to assess the flexibility of individual amino acid residues in the **3i** and **3j** complexes with **BCL2** and **BAX** over the course of the simulation. For the **BCL2** complexes, the RMSF values (Figure 8B) indicated that most residues exhibited low fluctuations (<0.5 nm), particularly those within structured regions of the protein. Slightly higher fluctuations were observed in the loop regions and terminal residues, which are inherently more flexible. Notably, the ligand‐binding residues, Arg146 and Asp140, demonstrated minimal fluctuations (0.11, 0.11 and 0.10 nm, 0.06 nm, respectively), suggesting that the ligand remained stably bound within the active site throughout the simulation.

##### 3.5.2.2 3i and 3j Complex with BAX

Similarly, for the BAX complexes, the RMSF values (Figure 9B) revealed that most residues exhibited low fluctuations (<0.15 nm), with higher flexibility observed in loop regions and terminal residues. The key ligand‐binding residue, Leu120, showed minimal fluctuations (0.20 nm for **3i** and 0.25 nm for **3j**), reinforcing the stability of the ligand–protein interaction. These findings suggest that both **3i** and **3j** maintained stable interactions with **BCL2** and **BAX**, with minimal perturbations in critical binding site residues, further supporting their potential as therapeutic inhibitors.

#### 3.5.3 Radius of Gyration (Rg)

##### 3.5.3.1 3i and 3j Complex with BCL2

The Rg provides insights into the compactness and structural integrity of the protein during the simulation. The Rg values of **3i** and **3j** with BAX complexes remained relatively stable around ∼1.46 nm^2^, indicating that the protein maintained its folded state without significant conformational changes (Figure 8C).

##### 3.5.3.2 3i and 3j Complex with BAX

The Rg values of both the complexes (**3i**, **3j** with BAX) remained relatively stable around ∼1.47 and ∼1.45 nm^2^, indicating that the protein maintained its folded state without significant conformational changes (Figure 9C).

#### 3.5.4 Hydrogen Bond Analysis

##### 3.5.4.1 3i and 3j Complex with BCL2

Hydrogen bonds play a vital role in ligand–protein binding stability. The hydrogen bond analysis revealed that the ligands **3i** and **3j** formed 4 and 3 hydrogen bonds with BCL2 wherein 2 hydrogen bonds were seen throughout the simulation, as depicted in Figure 8E,F. These hydrogen bonds remained consistent over time, suggesting strong ligand retention in the binding pocket.

##### 3.5.4.2 3i and 3j Complex with BAX

The hydrogen bond analysis revealed that the ligands **3i** and **3j** formed 3 and 5 hydrogen bonds with BAX wherein 1 hydrogen bond were seen throughout the simulation, as depicted in Figure 6B. These hydrogen bonds remained consistent over time, suggesting strong ligand retention in the binding pocket (Figure 8E,F).

#### 3.5.5 Solvent Accessible Surface Area (SASA)

##### 3.5.5.1 3i and 3j Complex with BCL2

The SASA of the protein–ligand complex was analyzed to understand changes in solvent exposure. The average SASA value for **3i** and **3j** complexes with **BCL2** was found to be 82.47 and 82.22 nm^2^, indicating that the overall solvent accessibility of the protein remained stable (Figure 8D).

##### 3.5.5.2 3i and 3j Complex with BAX

The average SASA value for **3i** and **3j** complexes with BAX was found to be 64.57 and 65.50 nm^2^, indicating that the overall solvent accessibility of the protein remained stable. A slight fluctuation in SASA was observed during the initial phase of the simulation to ∼65 ns, which correlated with the RMSD stabilization period. However, after equilibration, SASA remained constant, suggesting that the binding of the ligand did not significantly alter the protein's solvent exposure (Figure 9C).

### 3.6 Cytotoxicity and Selectivity Index (SI) Assessment Using MTT Assay

Compounds demonstrating pronounced cancer cell‐specific cytotoxicity while exhibiting minimal toxicity toward normal cells are considered promising candidates for anticancer therapy. In this context, a high SI serves as a critical parameter for advancing compounds to subsequent preclinical evaluations. The assay conducted across a concentration gradient enabled identification of 2 lead candidates, compounds **3i** and **3j**, from 15 synthesized molecules. These compounds exhibited the highest SI values, **2.30** and **4.44,** respectively, therefore selected for further comprehensive investigation (Table 2).

### 3.7 Quantitative and Qualitative Confirmation of Elevated Intracellular ROS Induced by 3i and 3j

**FIGURE 10:**
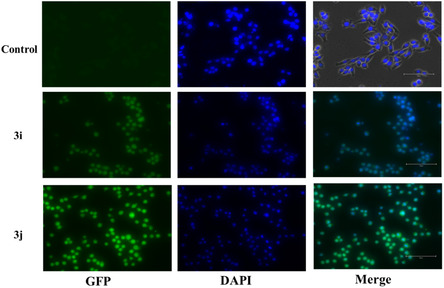
Qualitative analysis of ROS in MCF‐7 treated with **3i** and **3j**. Magnification‐ 20X, Scale Bar‐ 75 µm. The images are representative of three independent experiments.

Reactive oxygen species (ROS) have been widely investigated in relation to numerous human diseases, particularly cancers. These molecules naturally arise as byproducts of diverse cellular activities, such as oxygen metabolism [26]. Excessive ROS accumulation proves detrimental to cancer cells, ultimately triggering their destruction [27, 28]. Recent studies demonstrate that oxidative stress can suppress cancer progression and metastasis, while the GSH and thioredoxin (Trx) antioxidant systems‐regulated transcriptionally by Nrf2‐may contribute to tumor development and resistance to therapy [29]. This strategy involves elevating ROS levels in cancer cells to surpass the threshold required for cell death. This can be achieved either through direct ROS generation via exogenous methods or indirectly by increasing intracellular ROS concentrations through selective inhibition of endogenous antioxidant systems. Numerous studies suggest that the effectiveness of conventional cancer treatments, such as radiotherapy and chemotherapy, is largely attributed to their ability to induce high ROS levels, thereby facilitating cancer cell death [30, 31]. Antioxidants are often regarded as advantageous in both cancer prevention and treatment, as they help neutralize ROS, thereby lowering oxidative stress [32]. 2′, 7′‐dichlorofluorescein diacetate (DCFDA) is a well‐known non‐fluorescent, cell‐permeable compound used to study the ROS production. Upon entering the intra cellular space, they are deacetylated by the cellular esterases to produce membrane‐impermeable 2′, 7′‐dichlorofluorescein (DCFD) which will be trapped inside the cell. This nonfluorescent DCFD, in the presence of ROS can be oxidized by the cellular peroxidases to form a highly fluorescent DCF. Simply, the intensity of the green fluorescence is linearly related to the amount of ROS produced [33]. As shown in Figure 10, **3i** and **3j** had significantly higher levels of ROS induction in MCF‐7 after 24 h of treatment.

**FIGURE 11:**
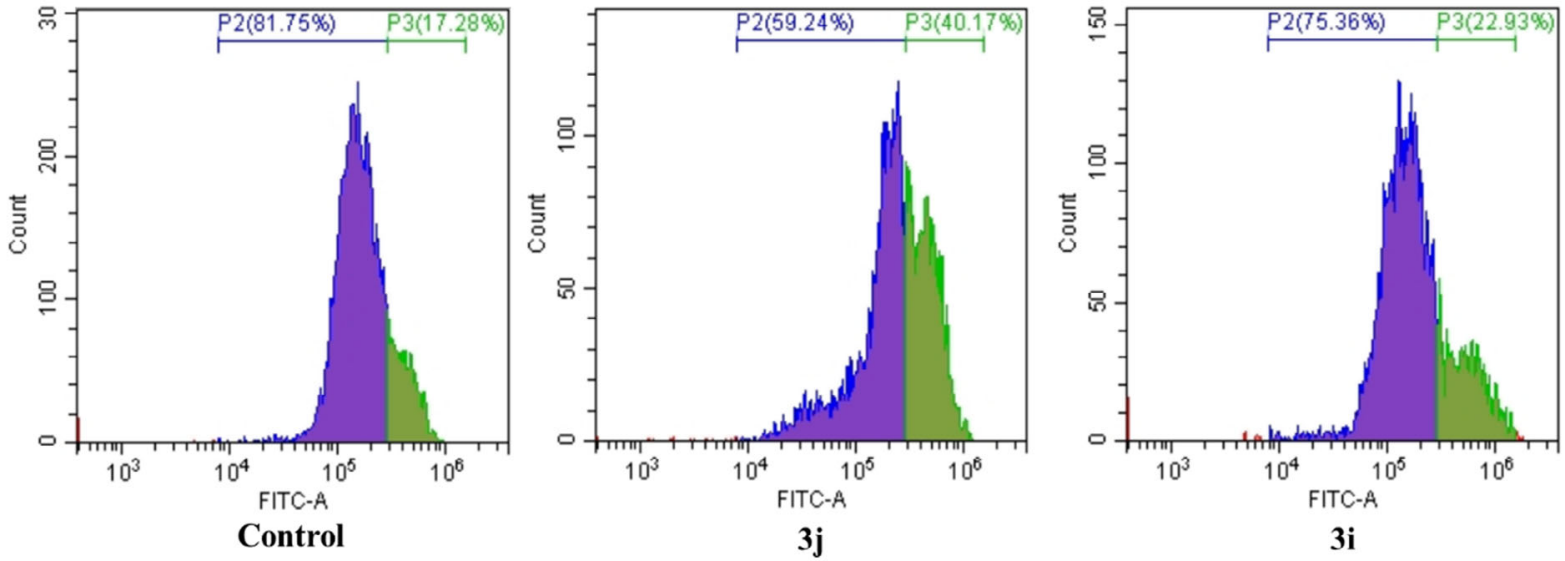
ROS generation in MCF‐7 induced by compounds **3i** and **3j** and quantified by flow cytometry.

The results of this experiment were further validated by quantifying the levels of ROS in flowcytometry. Quantitative flow cytometry (Figure 11) demonstrated that 18% of the cell population displayed basal ROS production. Compound treatment, significantly increased the proportion of ROS producing cells up to 40%, suggesting compound induced cellular lethality through a ROS‐mediated mechanism.

### 3.8 Compounds 3i and 3j Induced Oxidative Stress Leading to Depletion of Intracellular Glutathione (GSH)

**FIGURE 12:**
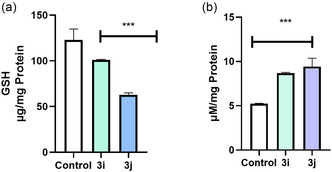
(a) Role of **3i/3j** on GSH depletion in MCF‐7. (b) The role of **3i/3j** on lipid peroxidation. Results are represented as mean ± SD where ***p < 0.001 versus control, **p < 0.01 versus control. Each experiments were repeated minimum three times.

ROS can trigger oxidative stress by interacting with lipids, initiating a feedback cycle through fatty acid peroxidation [34]. This modification disrupts the lipid bilayer of cell membranes and leads to free radical generation. Such oxidative damage poses a significant threat to cellular integrity, as peroxidation of mitochondrial phospholipids may compromise permeability transition pores (PTPs) and destabilize complexes I and III of the respiratory chain, ultimately increasing electron leakage within the mitochondrial intermembrane space. Increase in oxidative stress in cells can be quantified by checking lipid peroxidation inside cells [34]. LPO being a common marker of oxidative damage, is generally elevated in cancer cells due to their rapid growth and proliferation. Elevated levels of LPO primarily compromises membranal integrity by increasing cellular permeability contributing to their damage beyond repair. This mechanism can also lead to significant cellular dysfunction and ultimately trigger cell death [35]. As shown in Figure 12b treatment with lead compounds significantly increased MDA production thus further confirms the oxidative stress generation inside MCF‐7.

GSH plays a crucial role in the cellular antioxidant system. In cancer cells, elevated GSH levels are essential for neutralizing excessive ROS and detoxifying xenobiotics, making it a promising target for cancer treatment [36]. Extensive research has demonstrated that the depletion of intracellular GSH increases cancer cell sensitivity to oxidative stress and chemotherapeutic agents. Reducing GSH levels has been shown to enhance the effectiveness of ROS‐driven therapies, including photodynamic, sonodynamic, and chemodynamic approaches, as well as ferroptosis and conventional chemotherapy [37]. Reduced GSH plays a crucial role in regulating the cellular antioxidant defence mechanism by scavenging ROS and maintaining redox balance [38]. Figure 12a reveal that treatment with compounds **3i** and **3j** resulted in a significant depletion of intracellular GSH levels by ≈17% and 50%, respectively, relative to the control group.

Malignant cells usually have elevated levels of basal ROS, thus heavily depend on the body's antioxidant mechanism like GSH system to neutralize the free radicals. Depletion of basal GSH could lead to ROS accumulation, oxidatively damaging intracellular biomolecules eventually leading to cell death. Crucially, the observed increase in DCFH‐DA fluorescence intensity reflects the overall increase in the oxidative burden and is strongly corroborated by the independent GSH depletion data.

### 3.9 3i and 3j Trigger Intrinsic Apoptosis by Modulating Pro‐ and Anti‐Apoptotic markers and Disrupting Mitochondrial Membrane Potential (MMP)

Cancer cells rely heavily on ATP to sustain the anabolic pathways necessary for their rapid growth and proliferation. Mitochondria fulfill this demand by extracting energy from lipids, amino acids, and glucose through oxidation, transferring the resulting electrons to the electron transport chain (ETC), which subsequently donates them to molecular oxygen [39, 40]. The free energy preserved throughout this process is then harnessed to synthesize ATP [41]. Excessive ROS can compromise MMP by disrupting key elements of the ETC and triggering the mitochondrial permeability transition pore (MPTP). Elevated ROS levels may cause a swift depolarization of the inner mitochondrial membrane, ultimately hindering oxidative phosphorylation [42, 43]. MMP, the electrical potential gradient across the mitochondrial matrix and intermembrane space is critical for a healthy mitochondrial function. Severe and prolonged depletion of MMP leads to mitochondrial dysfunction characterized by the cessation of ATP synthesis and modifications in mitochondrial architecture, frequently initiating apoptosis‐mediated cell death [44].

**FIGURE 13:**
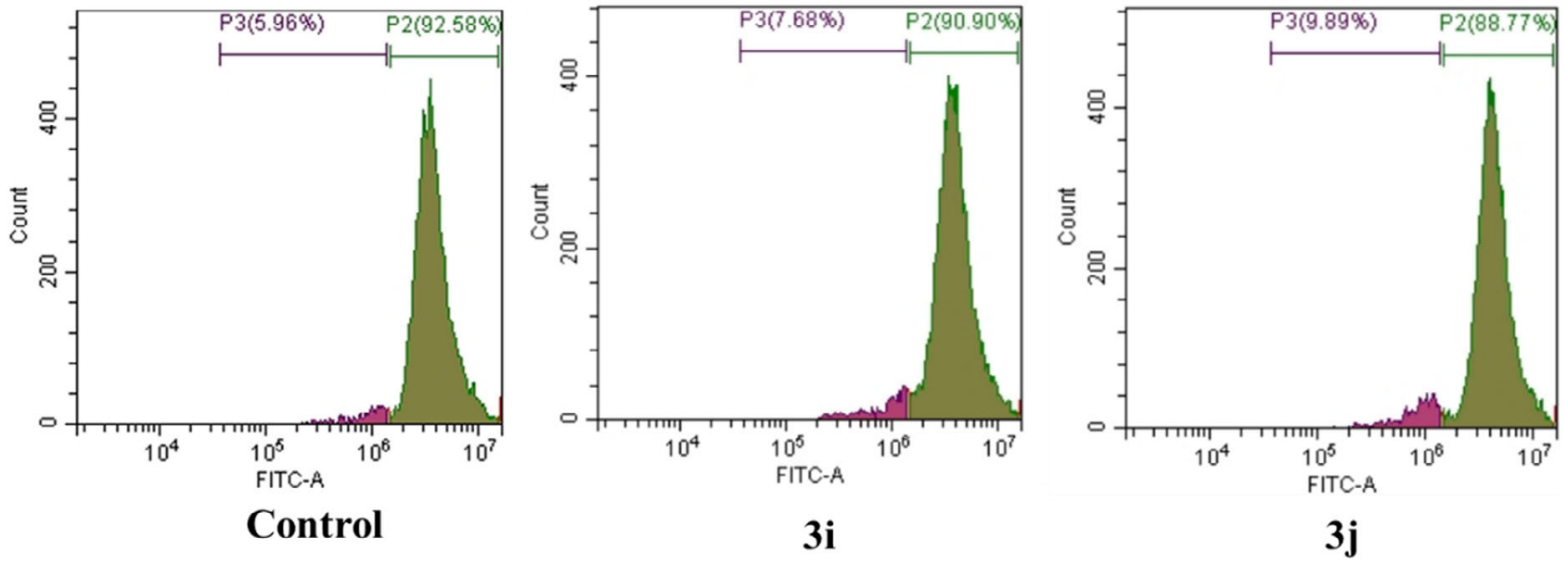
Quantitative analysis of MMP by flow cytometry in MCF‐7 treated with **3i** and **3j**.

The MMP status of the experimental cell populations was quantified using the lipophilic, cationic fluorescent dye, Rhodamine‐123. Paradoxically, healthy cells exhibiting normal MMP levels displayed lower fluorescence intensity due to the self‐quenching properties of the concentrated dye within the polarized mitochondrial matrix. Conversely, damaged or injured cell populations, characterized by a significant loss of MMP, exhibited brighter fluorescence intensity due to reduced dye accumulation and diminished self‐quenching [45]. Figure 13 numerically demonstrates a significant elevation in the proportion of fluorescent cell population, increasing from a baseline of 5% in control to 9% and 10% in the **3i** and **3j** treatment groups, respectively. This observation suggests the compound induced cell death by compromising the MMP.

Normal cells undergo apoptosis when they are damaged or stressed. However, cancer cells find ways to escape this normal apoptotic trigger felicitating their unconditional proliferation. Therefore, a potent anticancer drug should induce apoptosis‐mediated cell death to selectively eliminate neoplastic cells in a controlled manner, thereby minimizing systemic toxicity. However, previous reports also demonstrated that decrease in MMP and increase in oxidative stress triggers intracellular apoptosis induction by activating intrinsic pathway [46].

**FIGURE 14:**
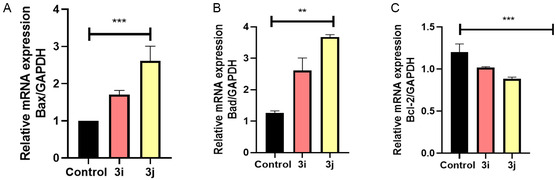
Effect of **3i/3j** on pro and antiapoptotic gene expression. (A,B) The expression of proapoptotic gene. (C) The expression of antiapoptotic gene. Data represent of three independent experiments as mean ± SD. ***p < 0.001 versus Control; **p < 0.05 versus Control.

Apoptosis is a well‐characterized form of regulated cell death (RCD) driven by caspase activation, protein cleavage, and the generation of apoptotic bodies [47]. Morphologically, it is distinguished by cell shrinkage, chromatin condensation, and the fragmentation of cells into apoptotic bodies [48]. Apoptotic signaling is classified into two main pathways: the extrinsic pathway, initiated by cell death receptors, and the intrinsic pathway, which arises from mitochondrial dysfunction [49]. The mitochondrial apoptotic pathway is activated in response to various stress signals, including mitochondrial dysfunction, endoplasmic reticulum (ER) stress, and oxidative damage [50]. The BCL2 protein family, consisting of ≈20 members, includes both proapoptotic and antiapoptotic regulators. Among the proapoptotic factors, BCL2‐associated X protein (BAX) and BCL2 antagonist/killer 1 (BAK1) play a crucial role in mitochondrial outer membrane permeabilization and the formation of the MPTP, thereby controlling pore formation in the outer mitochondrial membrane [51]. Apoptosis initiation is closely linked to ROS activity. Mitochondria act as the primary intracellular source of ROS, which arise from electron leakage in the respiratory ETC [42]. Excess mitochondrial ROS can damage adjacent cellular components, particularly mitochondrial DNA, rendering it susceptible to oxidative injury. This disruption affects the transcription of key proteins essential for ETC functionality, leading to mitochondrial dysfunction, impaired ATP synthesis, and a subsequent increase in ROS production. Elevated oxidative stress also contributes to the opening of the MPTP, ultimately triggering mitochondria‐mediated apoptosis. This effect is evident in the regulation of BCL2 family proteins by ROS, where increased levels of proapoptotic BAX and BAK1, along with reduced expression of antiapoptotic BCL2 and BCL2‐like 1 (BCL2L1/BCL‐XL), have been observed in squamous cell carcinoma cells [52] (Figure 14).

### 3.10 3i and 3j Induced Apoptotic Cell Death via Chromatin Condensation and Nuclear Fragmentation

**FIGURE 15:**
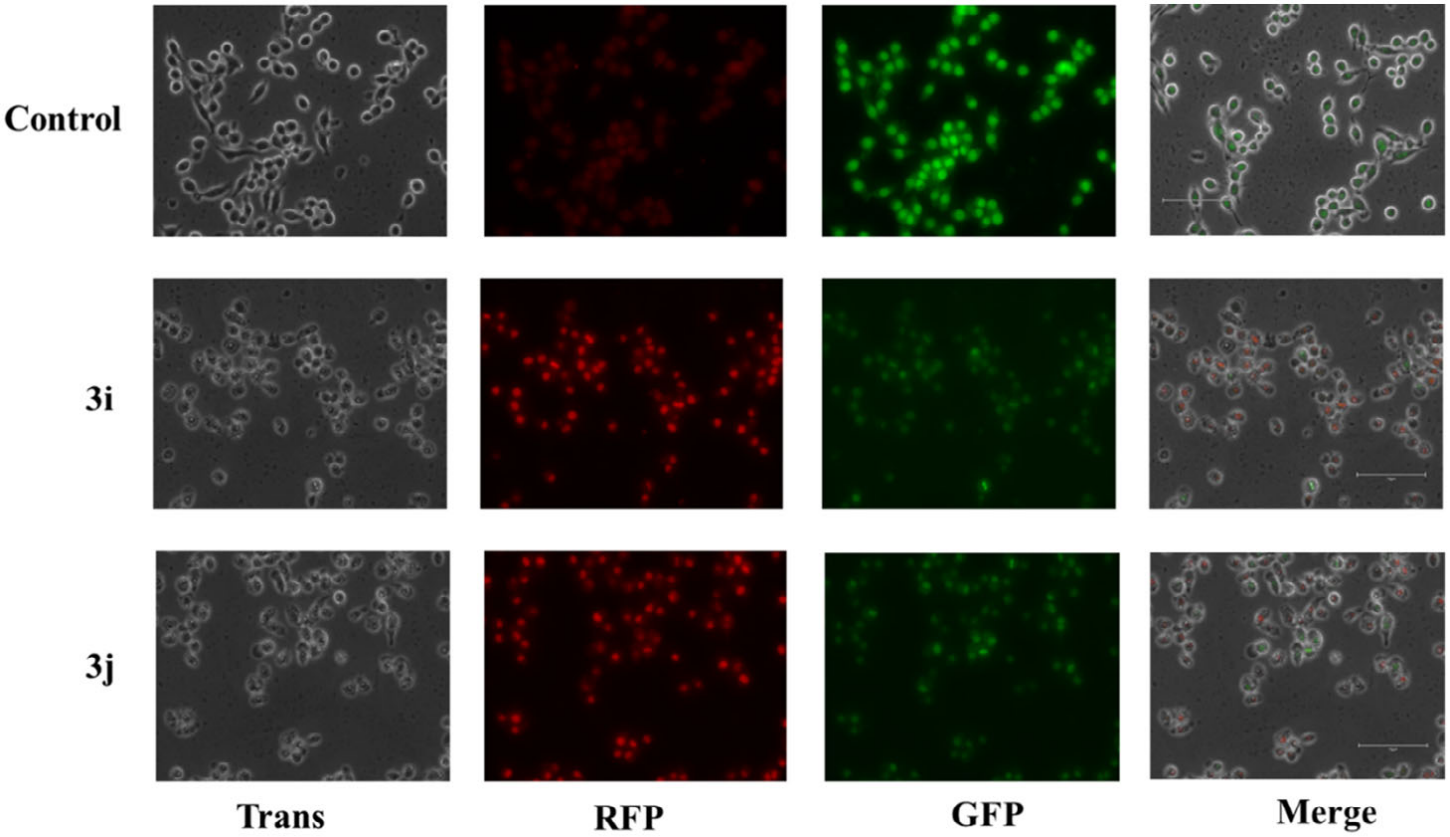
**3i** and **3j** induced cell apoptosis was observed using AO/EtBr staining. The images are representative of three independent experiments.

This study was confirmed using dual staining method AO/EtBr, the entire cell population is stained green by acridine orange (AO), a cell permeable nucleic acid intercalating dye, whereas, ethidium bromide (EB) is a cell impermeable dye staining only the dead cells by passing through damaged membranal layer [53]. Microscopic examination of stained cells (Figure 15) displayed bright green intact nuclei indicative of healthy live cells. In contrast, treated cells exhibited orange to red color stain, indicative of varying stages of apoptosis. While early apoptotic cells typically display greenish‐yellow color, the majority of cells in this study were stained from orange to dark red color, suggesting a predominance of late apoptosis. Further, a subset of cells even displayed distorted morphology, a common characteristic of necrosis. Further transcriptional profiling substantiated the compound driven apoptosis by exposing elevated and alleviated expression of pro‐apoptotic (Bax, Bad) and antiapoptotic genes (BCL2), respectively as shown in Figure 14. Specifically, versus control, **3j** treatment exhibited multiple‐fold differential expression, signifying efficient engagement of intrinsic apoptotic pathways.

**FIGURE 16:**
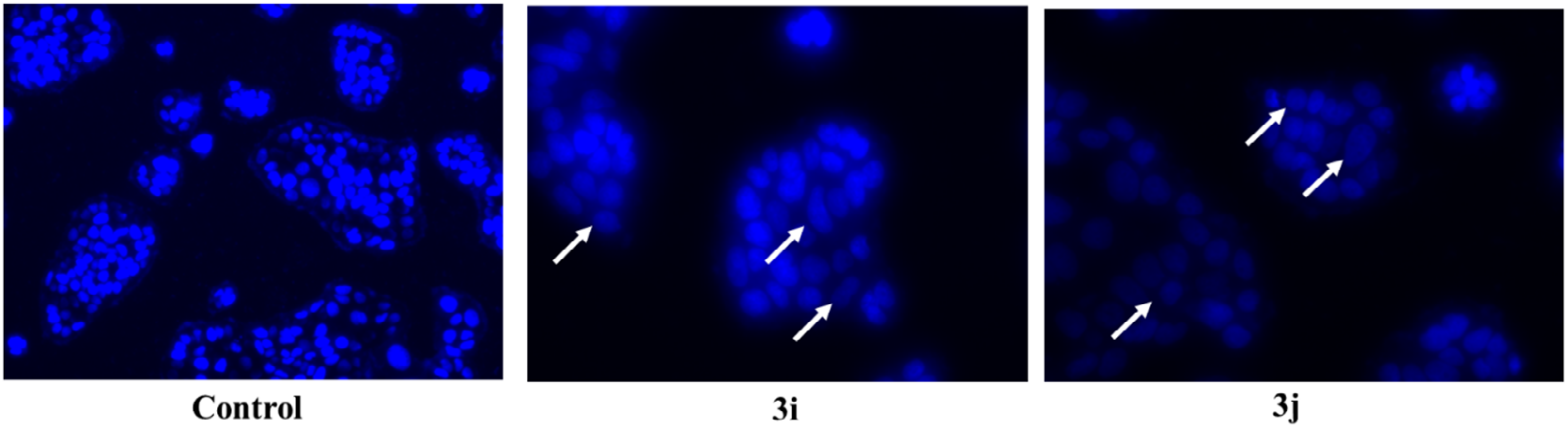
DNA fragmentation induced by **3i/3j** in MCF‐7 cells. The ligand induced DNA fragmentation indicated with white arrow was observed using DAPI staining. Images are representative of three individual experiments. Scale Bar‐150 µm.

This study was further confirmed using DAPI, a cell‐permeable DNA binding dye helps us to visualize the nuclear architectural alterations. Fluorescent images (Figure 16) revealed nuclei exhibiting normal size, shape and structural integrity, expressing optimal fluorescence intensity indicative of baseline DNA content. Relatively, compound treatment has significantly affected the nuclear morphology causing chromatin condensation, nuclear fragmentation and dissolution. Also, the overall fluorescence intensity is notably diminished due to the aforementioned factors (Figure 16).

### 3.11 Lead Compounds 3i and 3j Induced S‐Phase Cell Cycle Arrest

**FIGURE 17:**
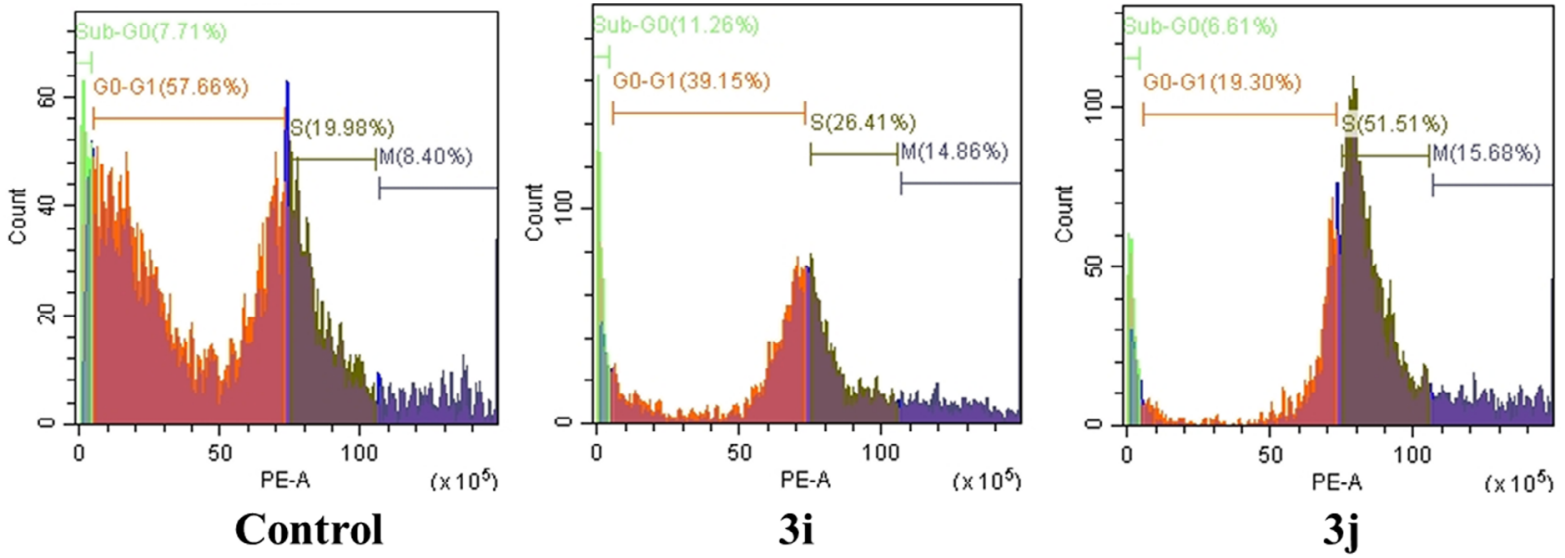
Cell cycle analysis in MCF‐7 treated with **3i/3j**.

Further analysis highlighted that both the lead compounds induced cell cycle arrest in S phase in MCF‐7 as shown in Figure 17. S phase cell cycle arrest in cancer cells plays a pivotal role in apoptosis regulation by initiating DNA damage response pathways, ultimately triggering programmed cell death. When replication forks stall or DNA damage occurs during S phase, checkpoint kinases Chk1 and Chk2 become activated, leading to the phosphorylation and activation of the tumor suppressor p53. Once activated, p53 can directly induce apoptosis or promote the expression of proapoptotic genes to facilitate cell death [54]. Previous studies have been reported that S phase arrest also triggers apoptosis [55] thus further confirm compound induced apoptosis in MCF‐7.

## 4 Conclusion

In summary, majority of the evaluated compounds displayed a favorable profile of tumor‐selective cytotoxicity. Particularly, compounds **3i** and **3j** distinguished themselves by exhibiting the highest therapeutic index. Extensive oncological characterization through in silico and in vitro studies revealed the promising potential for utilizing these compounds as novel antineoplastic therapeutics. In silico studies demonstrated stable interactions with the proapoptotic BAX and antiapoptotic BCL2 proteins, which are consistent with the findings from our in vitro experiments. Notably, **3j** significantly induced intracellular ROS generation, compromised cellular membrane integrity, depleted endogenous antioxidants, eventually contributing to the overall oxidative damage. The lead compounds instigated characteristic alterations in nuclear morphology and initiated a prolonged loss of MMP, resulting in significant mitochondrial dysfunction. Consequently, this cascade of cellular events triggered significant apoptosis by activating intrinsic pathway, which was mediated by the differential expression of the pro and antiapoptotic markers. The substantial accumulation of cells in the S phase demonstrates the compound's efficacy in disrupting DNA synthesis and impeding tumor cell proliferation. These results firmly establish the identified compounds as promising, selective anticancer lead candidates, for further preclinical development to overcome critical limitations in cancer treatment. Subsequent analyses are required to thoroughly assess the therapeutic utility and clinical potential of these compounds.

## Supporting Information

Additional supporting information can be found online in the Supporting Information Section. **Supporting Fig. S1–5:** ^1^H NMR, ^13^C NMR, IR spectra, HRMS and UPLC data of **3a**. **Supporting Fig. S6–10:** ^1^H NMR, ^13^C NMR, IR spectra, HRMS and UPLC data of **3b**. **Supporting Fig. S11–15:** ^1^H NMR, ^13^C NMR, IR spectra, HRMS and UPLC data of **3c**. **Supporting Fig. S15–20:** ^1^H NMR, ^13^C NMR, IR spectra, HRMS and UPLC data of **3d**. **Supporting Fig. S21–25:** ^1^H NMR, ^13^C NMR, IR spectra, HRMS and UPLC data of **3e**. **Supporting Fig. S25–30:** ^1^H NMR, ^13^C NMR, IR spectra, HRMS and UPLC data of **3f**. **Supporting Fig. S31–35:** ^1^H NMR, ^13^C NMR, IR spectra, HRMS and UPLC data of **3g**. **Supporting Fig. S36–40:** ^1^H NMR, ^13^C NMR, IR spectra, HRMS and UPLC data of **3h**. **Supporting Fig. S41–45:** ^1^H NMR, ^13^C NMR, IR spectra, HRMS and UPLC data of **3i**. **Supporting Fig. S46–50:** ^1^H NMR, ^13^C NMR, IR spectra, HRMS and UPLC data of **3j**. **Supporting Fig. S51–55:** ^1^H NMR, ^13^C NMR, IR spectra, HRMS and UPLC data of **3k**. **Supporting Fig. S56–60:** ^1^H NMR, ^13^C NMR, IR spectra, HRMS and UPLC data of **3l**. **Supporting Fig. S61–65:** ^1^H NMR, ^13^C NMR, IR spectra, HRMS and UPLC data of **3m**. **Supporting Fig. S65–70:** ^1^H NMR, ^13^C NMR, IR spectra, HRMS and UPLC data of **3n**. **Supporting Fig. S71–75:** ^1^H NMR, ^13^C NMR, IR spectra, HRMS and UPLC data of **3o**. **Supporting Table S1:** Information on forward and reverse primes. **Supporting Table S2:** Docking analysis of **3i** and **3j**, with BAX and BCL2 (PDB ID: 8SRY, 6O0K). **Supporting Table S3:** Optimized structure for energy and dipole moment of the selected compounds (**3i** and **3j**).

## Author Contributions

A.S. and V.M. have designed and supervised the project. J.M. performed all chemical synthesis, while A.S. and C.P. conducted the whole analytical characterization. T.D and P.B.N. have performed in vitro biological experiments. C.K.K. and I.E.A carried out the in silico study and data interpretation. S.M. and S.P. managed the reference section and performed some of the experiments. All the authors have contributed in preparing the manuscript.

## Conflicts of Interest

The authors declare no conflicts of interest.

## Supporting information

Supplementary Material

## Data Availability

The data that support the findings of this study are available within the manuscript supporting information of this article.
