# Supplementary material for: Integrated In Silico and In Vitro Study of Copper Nanocatalyzed Carbonyl‐Functionalized Triazoles—Inducing S Phase Cell Cycle Arrest and Apoptosis in MCF‐7
Source: ChemistryOpen. 2026 Jan 16;15(1):e202500543. doi: 10.1002/open.202500543 (PMC12810190; doi:10.1002/open.202500543)
Supplement: Supplementary file 1 — Supplementary Material [file OPEN-15-e202500543-s001.pdf]

Supporting Information

Integrated In Silico and In Vitro Study of Copper Nano-catalyzed Carbonyl-Functionalized Triazoles - Inducing S phase Cell Cycle Arrest and Apoptosis in MCF-7

Joydip Mondal [a], Tiasha Dasgupta [b], Chitluri Kiran Kumar [b], Prasanth Babu Nandagopal [b], Sadananda Mal [b], Sourav Paul [b], Aishwarya S [a], Chayan Pandya [a], Isaac Arnold Emerson [b], Venkatraman Manickam*[b] and Akella Sivaramakrishna*[a]

[a] Department of Chemistry, School of Advanced Sciences, Vellore Institute of Technology (VIT), Vellore 632014, Tamil Nadu, India

[b]Department of Bio-Medical Sciences, School of Biosciences and Technology, Vellore Institute of Technology (VIT), Vellore 632014, Tamil Nadu, India

E-mail ID of *Corresponding authors: venkatraman.m@vit.ac.in and asrkrishna@vit.ac.in

Sl. No.	Contents	Page No.
1	Information on forward and reverse prime*r*s	S3
2	General Procedure for the synthesis of (E)-3-phenyl-1-phenylprop-2-en-1-one (1a) and Benzyl azide (**2a**)	S3
3	General Procedure for the synthesis of (1-benzyl-5-phenyl-1H-1,2,3-triazol-4-yl)(phenyl)methanone (**3a**)	S4
4	*In-Silico* study	S4-S6
5	Structural characterization data of **3a-3p**	S7-S14
6	^{1}H NMR, ^{13}C NMR, IR spectra, HRMS and UPLC data of **3a-3o**	S15-S52

Table S1. Information on forward and reverse primers

Gene Name	Forward Primer	Reverse Primer
Bax	CCCGAGAGGTCTTTTTCCGAG	CCAGCCCATGATGGTTCTGAT
Bad	AGGATCCGTGCTGTCTCCTTTG	CAAAACTTCCGATGGGACCAAG
Bcl-2	TTGTGGCCTTCTTTGAGTTCGGTG	GGTGCCGGTTCAGGTACTCAGTCA
GAPDH	CATTGCCCTCAACGACCACTTT	GGTGGTCCAGGGGTCTTACTCC

General synthetic route for (E)-3-phenyl-1-phenyl prop-2-en-1-one ***(1a)*** *and benzyl azide* ***(2a)***

In a 50 mL round bottom flask (RBF), benzaldehyde (100 mg, 1.0 eq, 18.84 mmol) and acetophenone (172 mg, 1.0 eq, 16.64 mmol) were allowed to stir in 20 mL methanol for 10-15 minutes at ambient temperature, further aqueous solution of sodium hydroxide (2 eq) was transferred to the reaction mixture dropwise and allowed to stir for overnight. Completion of the reaction was observed by thin layer chromatography (TLC), followed by the addition of double distilled water resulting in the appearance of pale-yellow precipitate. To remove unreacted NaOH, the precipitate obtained was further filtered and washed with cold water twice. Finally, the desired pure chalcone (**1a**) was obtained by the recrystallization of yellow solid from ethanol solvent.

On the other hand, in an oven-dried 100 mL RBF, at 80 °C in an oil bath, benzyl bromide (100 mg, 1 eq, 11.68 mmol) was refluxed with sodium azide (77 mg, 2 eq, 23.38 mmol) in acetonitrile for 12 h. The progress of the reaction was monitored by TLC. After completion of the reaction, the mixture was allowed to cool at room temperature and further ethyl-acetate (25 mL) and water (25 mL) were added. Thereafter, the reaction mixture was extracted by ethyl acetate (3 × 25 mL) from mixture. Subsequently the separated organic phase was dried through Na_2SO_4 and then all the volatiles of the flask were removed by high vacuum pump. The obtained crude was purified using a silica gel column with a mixture of ethyl acetate-hexane as an eluant (1:9 v/v), which was further used for the synthesis of 1,2,3-trisubstituted triazoles.

General synthetic route for (1-benzyl-5-phenyl-1H-1,2,3-triazol-4-yl)(phenyl)methanone (**3a**)

In an oven-dried 100 mL RBF, (*E*)-3-phenyl-1-phenylprop-2-en-1-one (**1a**) (50 mg, 1 eq, 0.24 mmol) was allowed to reflux with benzyl azide (**2a**) (90 mg, 3 eq, 0.26 mmol) in solvent-free condition for 6 h at 75 °C. Completion of the reaction was observed by TLC. After completion of the reaction, the resulting mixture was allowed to cool at room temperature. Thereafter, the resulting mixture was extracted by ethyl acetate (3 × 25 mL). Later organic phase was dried through Na_2SO_4, concentrated under reduced pressure and purified by silica gel with a mixture of ethyl acetate-hexane as an eluant (4:6 v/v) with 95 % yield.

***In-silico* study**

Table S2- Docking analysis of 3i and 3j, with BAX and BCL2 (PDB ID: 8SRY, 6O0K)

**Target**	**Ligands**	**Hydrogen Bond interactions**	***π–π* stacking interactions**	**Hydrophobic interactions**
BAX (8SRY)	3i	Leu120	Phe92	Thr85, Pro88, Arg89, Glu90, Val91, Phe92, Phe93, Phe116, Ala117, Ser118, Lys119, Leu120, Val121, Leu122, Lys123 Ala124, Leu125, Ser126, Thr127, Lys128
	3j	Leu120	Phe92	Thr85, Ser87, Pro88, Arg89, Glu90, Val91, Phe92, Phe93, Arg94, Phe116, Ala117, Ser118,

				Lys119, Leu120, Val121, Leu122, Lys123 Ala124, Leu125, Ser126, Thr127, Lys128
BCL2 (6O0K)	3i	Arg146	Phe104, Tyr 108	Phe104, Ser105, Arg106, Arg107, Tyr108, Arg109, Arg110, Asp111, Phe112, Ala113, Glu114, Met115, Val133, Val134, Glu135, Glu136, Leu137, Phe138, Arg139, Asp140, Arg146, Ile147, Val148, Ala149, Phe150, Phe151, Glu152, Phe153, Gly154, Gly155, Val156
	3j	Asp140	Phe104, Tyr 108	Phe104, Ser105, Arg106, Arg107, Tyr108, Arg109, Arg110, Asp111, Phe112, Ala113, Glu114, Met115, Ser116, Val133, Val134, Glu135, Glu136, Leu137, Phe138, Arg139,

				Asp140, Gly141, Val142, Asn143, Trp144, Gly145, Arg146, Ile147, Val148, Ala149, Phe150, Phe151, Glu152, Phe153, Gly154, Gly155, Val156

Table S3. Optimized structure for energy and dipole moment of the selected compounds (**3i** and **3j**)

Compound	Structure	Energy (Hartree)	Dipole moment (D)
**3i**	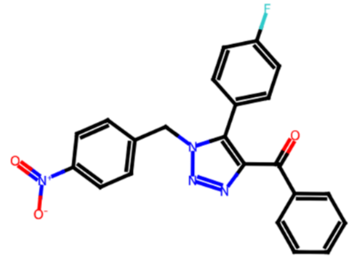	-1392.13011061	7.7386
**3j**	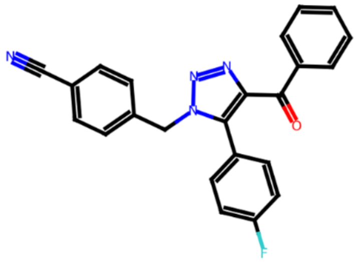	-1279.83346467	7.6111

Structural characterization data of 3a-3o

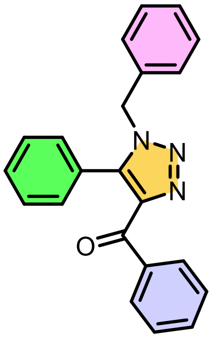

(1-benzyl-5-phenyl-1H-1,2,3-triazol-4-yl)(phenyl)methanone (3a)

White solid (325 mg, mp: 88-90 °C, yield: 96%). **^{1}H NMR** (400 MHz, $CDCl_3$) δ 8.21 (d, *J* = 7.32 Hz, 2H), 7.46 (t, *J* = 14.72 Hz, *J* = 7.36 Hz, 1H), 7.41-7.34 (m, 5H), 7.21-7.17 (m, 5H), 6.98 (m, 2H), 5.39 (s, 2H). **^{13}C NMR** (100 MHz, $CDCl_3$) δ 186.3, 143.8, 141.8, 137.1, 134.6, 133.0, 130.7, 130.0, 129.7, 128.8, 128.7, 128.4, 128.2, 127.6, 126.3, 52.0. **HRMS (ESI)**: calcd. for $C_{22}H_{17}N_3O$ [M+Na]: 362.1270, found: 362.1280. **IR** ν 3062.22, 2914.21, 2852.29, 1650.84, 1441.67, 1203.04, 908.54, 688.03 cm^{-1}.

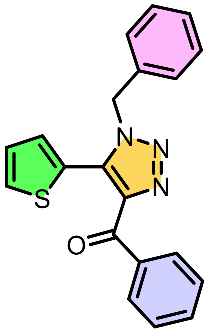

(1-benzyl-5-(thiophen-2-yl)-1H-1,2,3-triazol-4-yl)(phenyl)methanone (3b)

Off white solid (272 mg, mp: 75-78 °C, yield: 79%). **^{1}H NMR** (400 MHz, $CDCl_3$) δ 8.17 (d, *J* = 7.48 Hz, 2H), 7.50-7.48 (m, 2H), 7.40 (t, *J* = 14.92 Hz, *J* = 7.48 Hz, 2H), 7.20 (d, *J* = 21.92 Hz, 3H), 7.07 (dd, *J* = 22.04 Hz, *J* = 2.48 Hz, 4H), 5.53 (s, 2H). **^{13}C NMR** (100 MHz, $CDCl_3$) δ 186.3, 144.3, 136.9, 135.2, 134.6, 133.2, 131.3, 130.7, 129.7, 128.9, 128.5, 128.2, 127.4, 127.4, 124.89, 52.3. **HRMS (ESI)**: calcd. for $C_{20}H_{15}N_3OS$ [M+Na]: 368.0834, found: 368.0850. **IR** ν 3090.16, 2959.52, 2914.21, 2840.96, 1650.85, 1452.99, 903.25, 688.03 cm^{-1}.

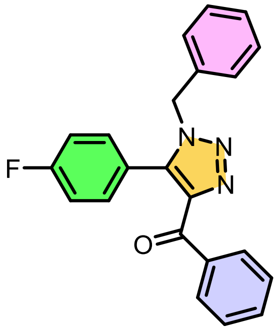

(1-benzyl-5-phenyl-1H-1,2,3-triazol-4-yl)(phenyl)methanone (3c)

White solid (339 mg, mp: 128-130 ºC, yield: 95%).**^{1}H NMR** (400 MHz, $CDCl_3$) δ 8.21 (m, 2H), 7.53-7.49 (m, 1H), 7.49-7.39 (m, 2H), 7.22 (m, 3H), 7.18 (m, 2H), 7.06 (m, 2H), 6.99 (m, 2H), 5.39 (s, 2H). **^{13}C NMR** (100 MHz, $CDCl_3$) δ 185.3, 163.8, 161.3, 142.9, 139.8, 135.9, 133.5, 132.1, 130.9, 130.8, 129.6, 127.9, 127.5, 127.2, 126.5, 121.2, 121.2, 115.0, 114.8, 51.1. **HRMS (ESI)**: calcd. for $C_{22}H_{16}FN_3O$ [M+Na]: 380.1175, found: 380.1181. **IR** ν 2959.52, 2920.25, 2846.25, 1730.14, 1650.85, 1214.37, 710.69 cm^{-1}.

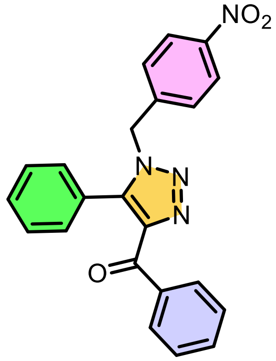

(1-(4-nitrobenzyl)-5-phenyl-1H-1,2,3-triazol-4-yl)(phenyl)methanone (3d)

Off white solid (311 mg, mp: 138-140 °C. yield: 81%). **^{1}H NMR** (400 MHz, $CDCl_3$) δ 8.27 (t, J = 8.52 Hz, J = 7.12 Hz, 2H), 8.16 (m, 2H), 7.61-7.57 (m, 1H), 7.54-7.44 (m, 5H), 7.24 (t, J = 11.4 Hz, J = 2.36 Hz, 4H), 5.58 (s, 2H). **^{13}C NMR** (100 MHz, $CDCl_3$) δ 185.0, 146.9, 140.9, 140.3, 135.8, 132.2, 129.6, 129.3, 128.5, 127.9, 127.5, 127.2, 123.1, 50.1. **HRMS (ESI)**: calcd. for $C_{22}H_{16}N_4O_3$ [M+Na]: 407.1120, found: 407.1110. **IR** ν 3067.50, 2954.23, 1656.89, 1514. 92, 1338.97, 914.58, 688.03 cm^{-1}.

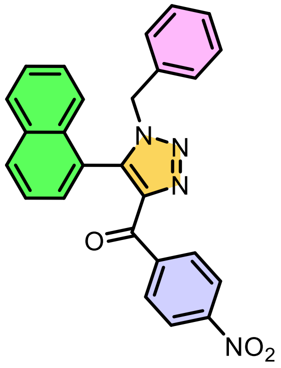

(1-benzyl-5-(naphthalen-1-yl)-1H-1,2,3-triazol-4-yl)(4-nitrophenyl)methanone (3e)

Yellow solid (347 mg, mp: 136-138 °C. yield: 80%). **^{1}H NMR** (400 MHz, $CDCl_3$) δ 8.50 (d, *J* = 8.72 Hz, 2H), 8.28 (d, *J* = 8.68, 2H), 8.06 (d, *J* = 8.24 Hz, 1H), 7.94 (d, *J* = 8.2 Hz, 1H), 7.53 (m, 2H), 7.35 (t, *J* = 15.2 Hz, *J* = 7.96 Hz, 1H), 7.26 (d, *J* = 5.16 Hz, 1H), 7.20-7.09 (m, 4H), 6.85 (d, *J* = 7.44 Hz, 2H), 5.45 (d, *J* = 14.68 Hz, 1H), 5.19 (d, *J* = 14.68 Hz, 1H). **^{13}C NMR** (100 MHz, $CDCl_3$) δ 183.8, 150.0, 144.5, 141.7, 141.0, 133.8, 133.4, 131.6, 131.2, 130.9, 128.8, 128.6, 128.5, 128.3, 128.1, 127.4, 126.6, 125.0, 124.0, 123.5, 123.3, 52.6. **HRMS (ESI):** calcd. for $C_{26}H_{18}N_4O_3$ [M+Na]: 457.1277, found: 457.1263. **IR** ν 2959.52, 2920.25, 2846.25, 1650.85, 1520.96, 1338.97, 908.53, 693.32 cm^{-1}.

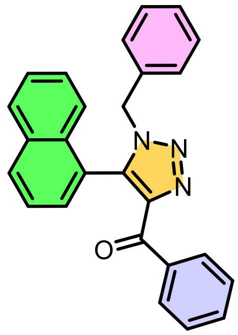

(1-benzyl-5-(naphthalen-1-yl)-1H-1,2,3-triazol-4-yl)(phenyl)methanone (3f)

Yellow solid (280 mg, mp: 125-127 °C. yield: 72%). **^{1}H NMR** (400 MHz, $CDCl_3$) δ 8.23 (d, *J* = 7.68 Hz, 2H), 7.89 (d, *J* = 8.2 Hz, 1H), 7.63 (d, *J* = 8.16 Hz, 1H), 7.45-7.34 (m, 5H), 7.24 (t, *J* = 15.2 Hz, *J* = 7.96 Hz, 1H), 7.13 (d, *J* = 6.68 Hz, 1H), 7.06-6.87 (m, 4H), 6.75 (d, *J* = 7.36 Hz, 2H), 5.34(d, *J* = 14.76 Hz, 1H), 5.13 (d, *J* = 14.76 Hz, 1H). **^{13}C NMR** (100 MHz, $CDCl_3$) δ 185.7, 145.3, 140.2, 136.9, 134.1, 133.4, 132.9, 131.4, 130.6, 130.5, 128.6, 128.5, 128.5, 128.3, 128.2, 128.0, 127.2, 126.4, 125.0, 124.2, 124.0, 52.4. **HRMS (ESI):** calcd. for $C_{26}H_{19}N_3O$ [M+Na]: 412.1426, found: 412.1431. **IR** ν 3060.64, 2860.18, 2231.35, 1685.58, 1597.71, 840.50 cm^{-1}

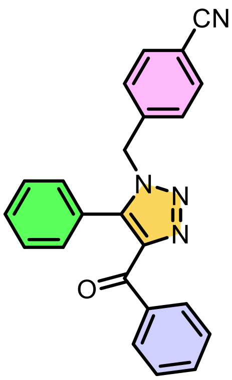

4-((4-benzoyl-5-phenyl-1H-1,2,3-triazol-1-yl)methyl)benzonitrile (3g)

Off white solid (317 mg, mp: 122-124 °C. yield: 87%). ^{1}**H NMR** (400 MHz, $CDCl_3$) δ 8.34 (d, *J* = 7.52, Hz, 2H), 7.52-7.49(m, 3H), 7.41-7.37(m, 5H), 7.18 (t, *J* = 11.32 Hz, *J* = 4.28 Hz, 2H), 7.12 (d, *J* = 8.12 Hz, 2H), 5.45 (s, 2H). ^{13}**C NMR** (100 MHz, $CDCl_3$) δ 186.1, 143.9, 141.9, 139.5, 136.9, 133.2, 132.7, 130.6, 130.3, 129.6, 128.9, 128.3, 128.3, 125.9, 118.1, 112.6, 51.4. **HRMS (ESI):** calcd. for $C_{23}H_{16}N_4O$ [M+Na]: 387.1222, found: 387.1228. **IR** ν 2954.23, 2914.21, 2852.29, 2228.53, 1645.56, 1452.99, 1197.76, 908.53, 693.32 cm^{-1}.

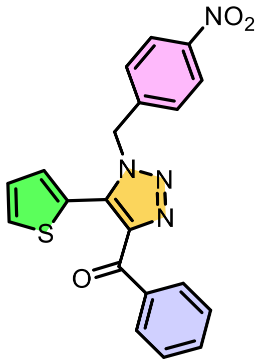

(1-(4-nitrobenzyl)-5-(thiophen-2-yl)-1H-1,2,3-triazol-4-yl)(phenyl)methanone (3h)

Brown solid (269 mg, mp: 102-104 °C. yield: 69%). ^{1}**H NMR** (400 MHz, $CDCl_3$) δ 8.17 (dd, *J* = 22.4 Hz, *J* = 7.56 Hz, 4H), 7.54 (m, 2H), 7.43 (t, *J* = 15.24, *J* = 7.72 Hz, 2H), 7.23 (d, *J* = 8.56 Hz, 2H), 7.13 (d, *J* = 2.88 Hz, 1H), 7.06 (t, *J* = 8.64, *J* = 4 Hz, 1H), 5.64 (s, 2H). ^{13}**C NMR** (100 MHz, $CDCl_3$) δ 186.1, 148.0, 144.5, 141.4, 136.7, 135.3, 133.3, 131.5, 130.7, 130.1, 128.3, 127.7, 124.3, 124.2, 51.5. **HRMS (ESI):** calcd. for $C_{20}H_{14}N_4O_3S$ [M+Na]: 413.0685, found: 413.0688. **IR** ν 3091.53, 2957.67, 2916.47, 2849. 88, 1654.69, 1515.33, 1334.78, 696.34.

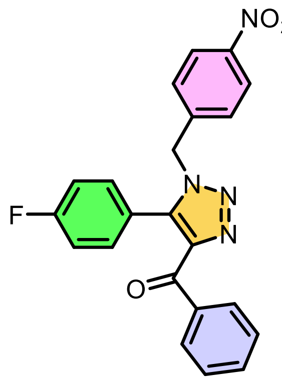

(5-(4-fluorophenyl)-1-(4-nitrobenzyl)-1H-1,2,3-triazol-4-yl)(phenyl)methanone (3i)

Off white solid (325 mg, mp: 138-140 °C. yield: 81%). ^{1}**H NMR** (400 MHz, $CDCl_3$) δ 8.22 (d, *J* = 7.72 Hz, 2H), 8.13 (d, *J* = 8.64, 2H), 7.54 (t, *J* = 14.72, *J* = 7.2 Hz, 1H), 7.43 (t, *J* = 15.36 Hz, *J* = 7.8 Hz, 2H), 7.18 (t, *J* = 14.16 Hz, *J* = 6.32, 4H), 7.08 (t, *J* = 16.96, *J* = 8.4, 2H), 5.50 (s, 2H). ^{13}**C NMR** (100 MHz, $CDCl_3$) δ 186.0, 141.2, 136.7, 133.3, 131.8, 131.7, 130.6, 128.4, 128.3, 124.2, 116.4, 116.2, 51.1. **HRMS (ESI):** calcd. for $C_{22}H_{15}FN_4O_3$ [M+Na]: 425.1026, found: 425.1031. **IR** ν 3070.92, 2952.86, 2916.47, 2849.88, 1649.19, 1515.33, 1345.08, 907.78, 907.78 cm^{-1}.

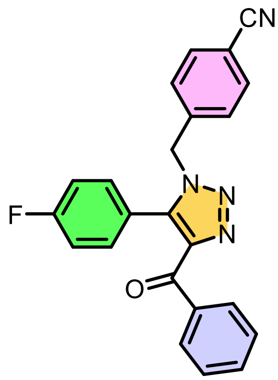

4-((4-benzoyl-5-(4-fluorophenyl)-1H-1,2,3-triazol-1-yl)methyl)benzonitrile (3j)

White solid (325 mg, mp: 140-143 °C. yield: 85%). ^{1}**H NMR** (400 MHz, $CDCl_3$) δ 8.21 (d, *J* = 7.92 Hz, 2H), 7.53 (t, *J* = 15.08 Hz, *J* = 8.08 Hz, 3H), 7.42 (t, *J* = 15.24 Hz, *J* = 7.4 Hz, 2H), 7.15 (m, 2H), 7.11-7.06 (m, 4H), 5.45 (s, 2H). ^{13}**C NMR** (100 MHz, $CDCl_3$) δ 186.1, 165.0, 162.5, 144.0, 141.0, 139.4, 136.7, 133.3, 132.8, 131.8, 131.7, 130.6, 128.3, 128.2, 121.8, 121.8, 118.0, 116.3, 116.1, 112.8, 51.4. **HRMS (ESI):** calcd. for $C_{23}H_{15}FN_4O$ [M+Na]: 405.1128, found: 405.1126. **IR** ν 3066.13, 2957.66, 2911.67, 2849.88, 2231.35, 1644.39, 907.78, 696.34 cm^{-1}.

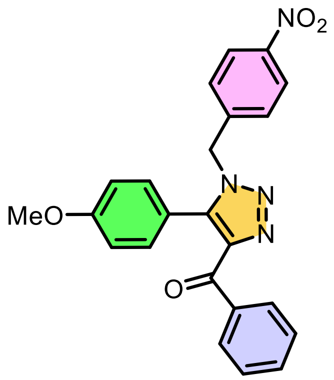

(5-(4-methoxyphenyl)-1-(4-nitrobenzyl)-1H-1,2,3-triazol-4-yl)(phenyl)methanone (3k)

Dark yellow solid (343 mg, mp: 137-140 °C. yield: 83%). **^{1}H NMR** (400 MHz, $CDCl_3$) δ 8.21 (t, *J* = 8.64 Hz, *J* = 7.28 Hz, 2H), 8.15 (d, *J* = 8.68 Hz, 2H), 7.52 (t, *J* = 14.76 Hz, *J* = 7.32 Hz, 1H), 7.43 (t, *J* = 15.32 Hz, *J* = 7.84 Hz, 2H), 7.15 (t, *J* = 8.6 Hz, *J* = 4.28 Hz, 2H), 7.12 (m, 2H), 6.88 (d, *J* = 5.16 Hz, 2H), 5.50 (s, 2H), 3.78 (s, 3H). **^{13}C NMR** (100 MHz, $CDCl_3$) δ 186.2, 161.1, 147.9, 143.7, 141.9, 141.6, 137.0, 133.1, 131.1, 130.6, 128.4, 128.2, 124.1, 117.4, 114.4, 55.4, 51.0. **HRMS (ESI):** calcd. for $C_{23}H_{18}N_4O_4$ [M+H]: 415.1406, found: 415.1431. **IR** ν 2952.86, 2921.96, 2870.48, 1500.23, 1200.91, 1015.56, 815.10 cm^{-1}.

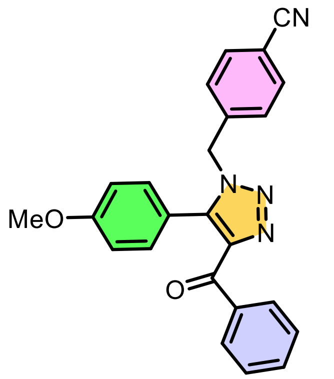

4-((4-benzoyl-5-(4-methoxyphenyl)-1H-1,2,3-triazol-1-yl)methyl)benzonitrile (3l)

Off white solid (354 mg, mp: 123-125 °C. yield: 90%). **^{1}H NMR** (400 MHz, $CDCl_3$) δ 8.23 (d, *J* = 7.52 Hz, 2H), 7.54-7.49 (m, 3H), 7.41 (t, *J* = 15.24 Hz, *J* = 7.72 Hz, 2H), 7.13 (q, *J* = 3.68 Hz, 4H), 6.87 (d, *J* = 8.6 Hz, 2H), 5.45 (s, 2H), 3.78 (s, 3H). **^{13}C NMR** (100 MHz, $CDCl_3$) δ 186.2, 161.1, 143.7, 141.8, 139.8, 137.0, 133.1, 132.7, 131.1, 130.6, 128.2, 118.1, 117.5, 114.4, 112.6, 55.4, 51.2. **HRMS (ESI):** calcd. for $C_{24}H_{18}N_4O_2$ [M+Na]: 417.1328, found: 417.1335. **IR** ν 2957.66, 2926.77, 2849.88, 2226.54, 1721.28, 1489.93, 1227.00, 753.32 cm^{-1}.

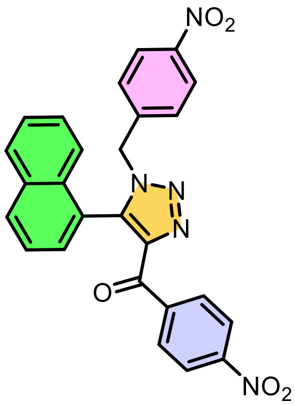

(5-(naphthalen-1-yl)-1-(4-nitrobenzyl)-1H-1,2,3-triazol-4-yl)(4-nitrophenyl)methanone (3m)

yellow solid (383 mg, mp: 125-127 °C. yield: 80%). **^{1}H NMR** (400 MHz, DMSO-*d6*) δ 8.35 (s, 4H), 8.12 (d, *J* = 7.72, 1H), 7.95 (m, 3H), 7.57 (m, 2H), 7.51 (t, *J* = 14.96 Hz, *J* = 7.48 Hz, 1H), 7.34 (t, *J* = 15.2 Hz, *J* = 8.04, 1H), 7.15 (m, 3H), 5.68 (m, 2H). **^{13}C NMR** (100 MHz, DMSO-*d6*) δ 184.7, 150.1, 147.3, 144.5, 142.3, 142.1, 141.1, 133.3, 131.7, 131.1, 129.3, 129.0, 128.8, 127.6, 126.8, 125.7, 124.6, 123.8, 123.8, 123.5, 51.5. **HRMS (ESI):** calcd. for $C_{26}H_{17}N_5O_5$ [M+H]: 480.1308, found: 480.1302. **IR** ν 3189.70, 2926.77, 2226.54, 1721.28, 1448.74, 1231.80, 799.31 cm^{-1}.

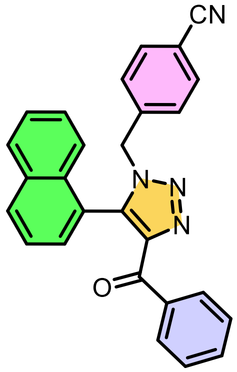

4-((4-benzoyl-5-(naphthalen-1-yl)-1H-1,2,3-triazol-1-yl)methyl)benzonitrile (3n)

Yellow solid (294 mg, mp: 123-125 °C. yield: 71%). **^{1}H NMR** (400 MHz, $CDCl_3$) δ 8.24 (d, *J* = 7.6 Hz, 2H), 7.93 (d, *J* = 8.28 Hz, 1H), 7.84 (d, *J* = 8.2, 1H), 7.51-7.34 (m, 5H), 7.32-7.24 (m, 5H), 6.96 (d, *J* = 8.4 Hz, 1H), 6.83 (d, *J* = 7.88 Hz, 2H), 5.34 (d, *J* = 15.04 Hz, 1H), 5.21 (d, *J* = 15.08, 1H). **^{13}C NMR** (100 MHz, $CDCl_3$) δ 185.5, 145.4, 140.4, 138.9, 136.7, 133.4, 133.1, 132.3, 131.2, 130.8, 130.6, 128.7, 128.7, 128.2, 127.4, 126.7, 125.0, 124.0, 123.6, 118.0, 112.4, 51.8. **HRMS**

(ESI): calcd. for $C_{27}H_{18}N_4O$ [M+H]: 437.1379, found: 480.1384. **IR** ν 3060.64, 2926.77, 2231.35, 1649.19, 1448.74, 1216.70, 907.78, 691.53 cm^{-1}.

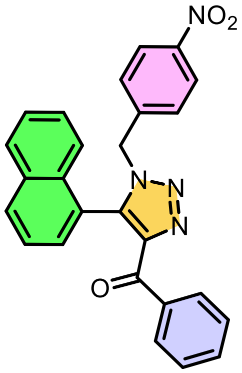

(5-(naphthalen-1-yl)-1-(4-nitrobenzyl)-1H-1,2,3-triazol-4-yl)(phenyl)methanone (3o)

Yellow solid (317 mg, mp: 115-118 °C. yield: 72%). ^{1}**H NMR** (400 MHz, $CDCl_3$) δ 8.24 (d, J = 7.64 Hz, 2H), 7.93 (d, J = 8.28 Hz, 1H), 7.84 (t, J = 17.12 Hz, J = 8.6 Hz, 3H), 7.51-7.36 (m, 6H), 7.25-7.19 (m, 2H), 6.98 (d, J = 8.36 Hz, 1H), 6.96 (d, J = 8.48 Hz, 2H), 5.41 (d, J = 15.12 Hz, 1H), 5.23 (d, J = 15.12 Hz, 1H). ^{13}**C NMR** (100 MHz, $CDCl_3$) δ 185.5, 147.7, 145.4, 140.7, 140.4, 136.7, 133.4, 133.1, 131.2, 130.9, 130.6, 128.9, 128.8, 128.3, 128.2, 127.9, 127.8, 127.4, 127.3, 126.7, 125.1, 124.1, 123.9, 123.7, 123.6, 51.5. **HRMS (ESI):** calcd. for $C_{26}H_{18}N_4O_3$ [M+H]: 457.1277, found: 457.1263. **IR** ν 2911.67, 2860.18, 1690.38, 1494.74, 1206.41, 835.69 cm^{-1}.

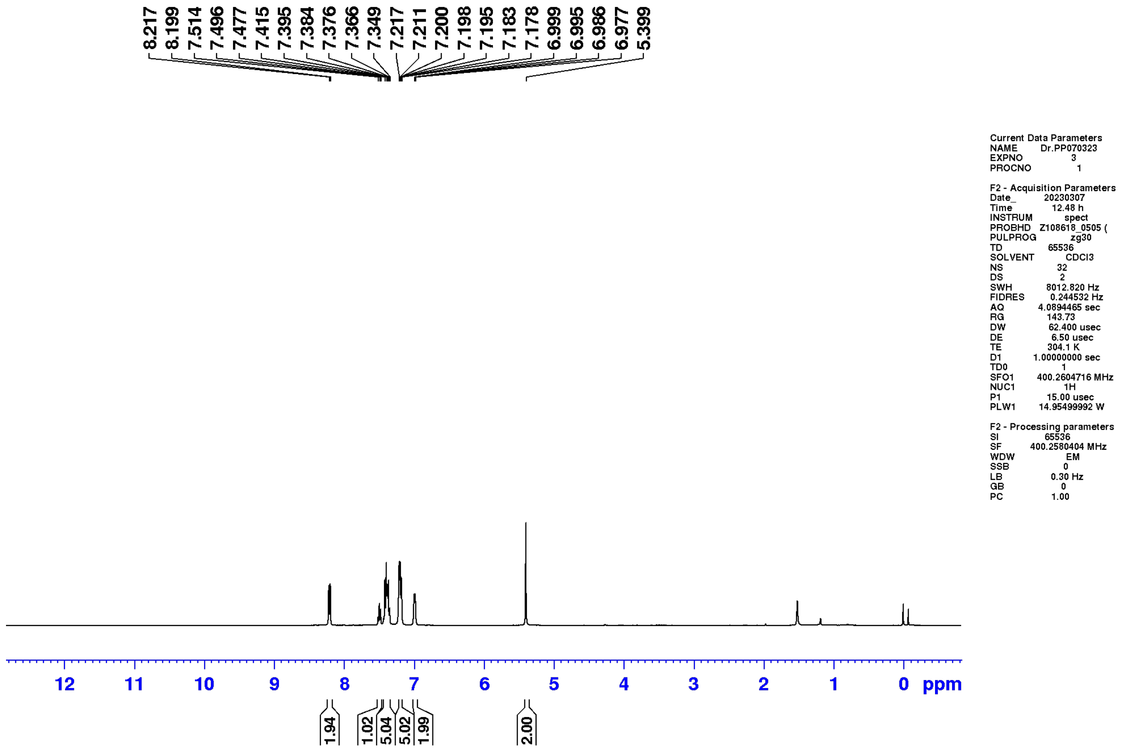

Figure S1. ^{1}H NMR of **3a**

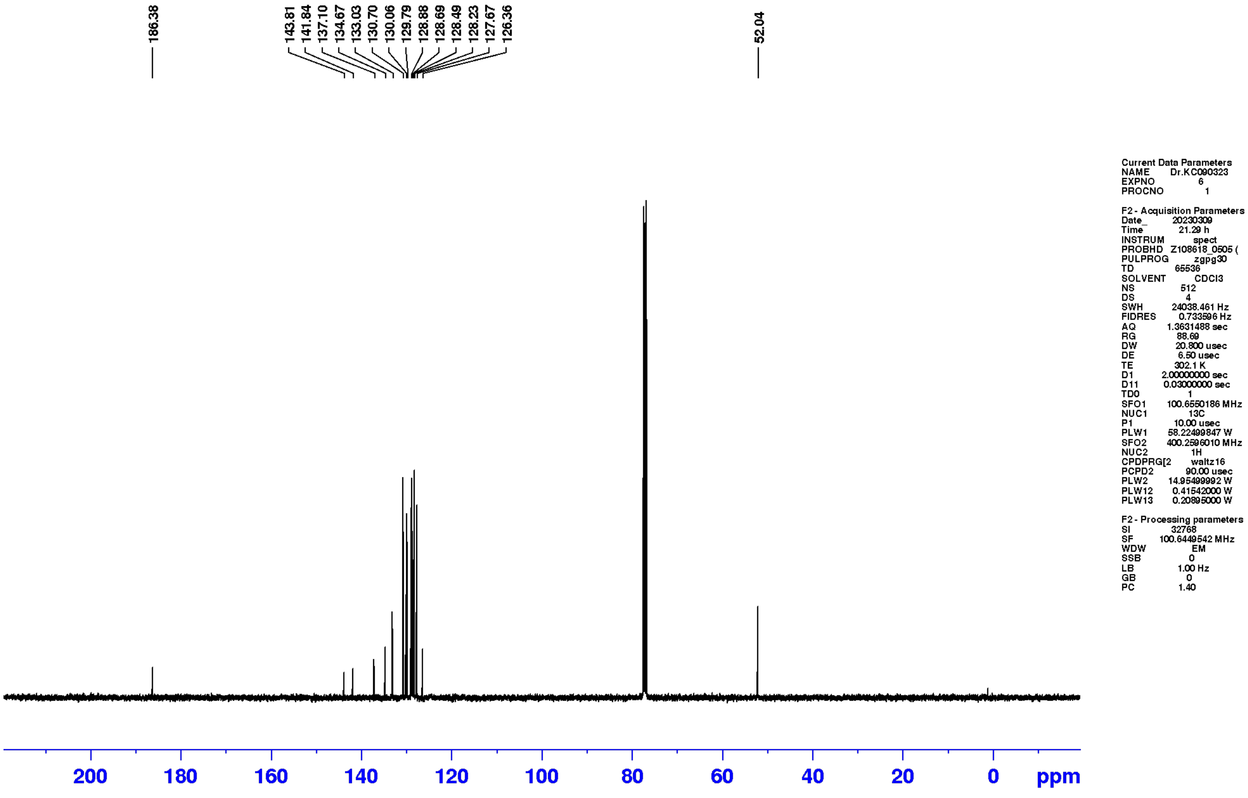

Figure S2. ^{13}C NMR of **3a**

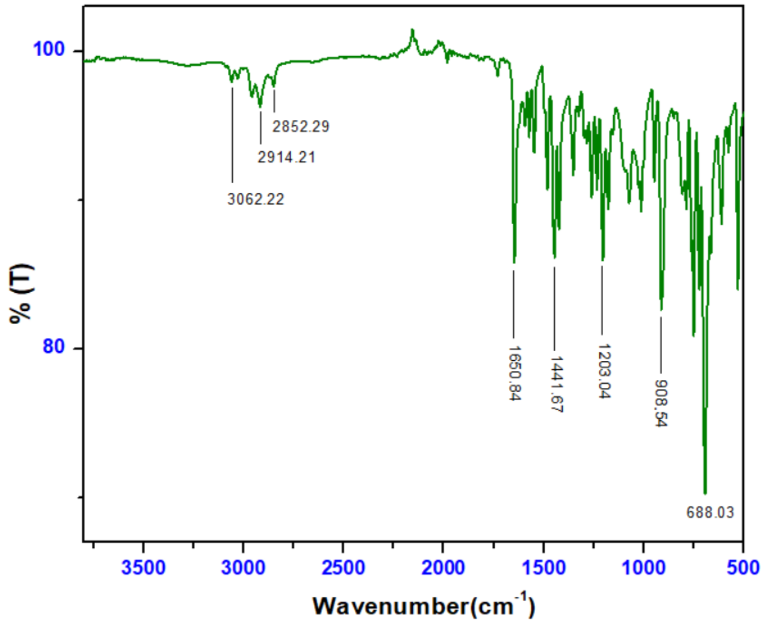

Figure S3. IR spectrum of **3a**

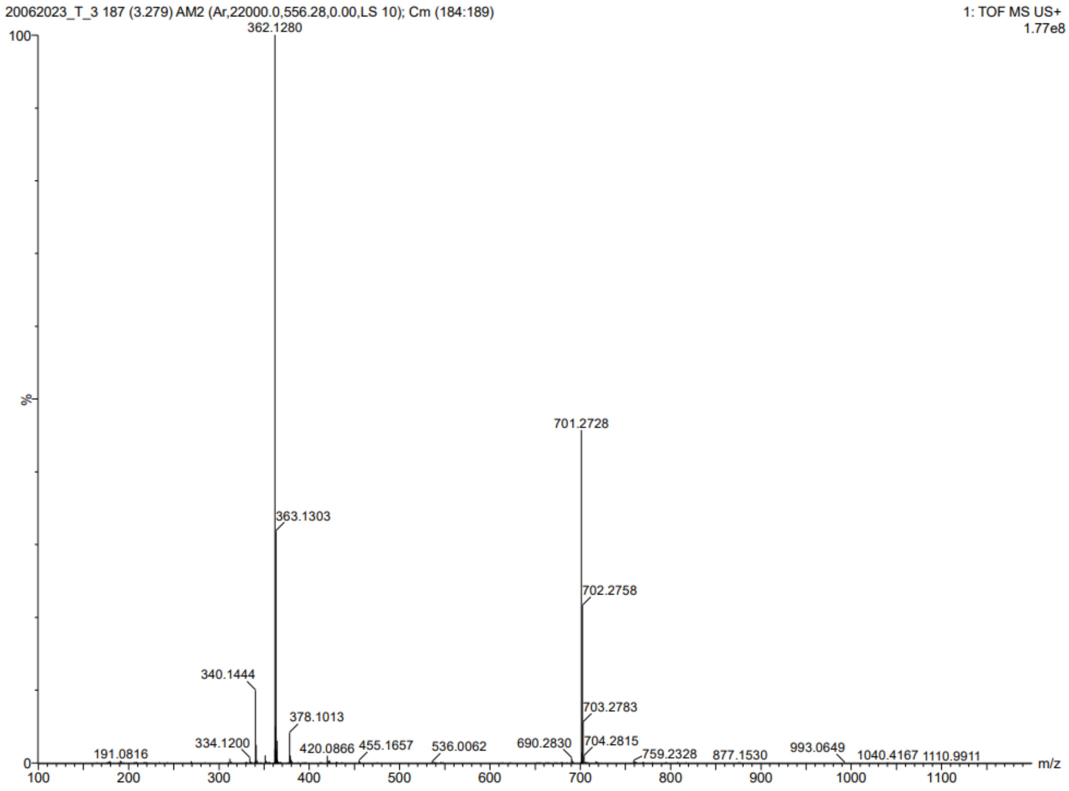

Figure S4. ESI HRMS of **3a**

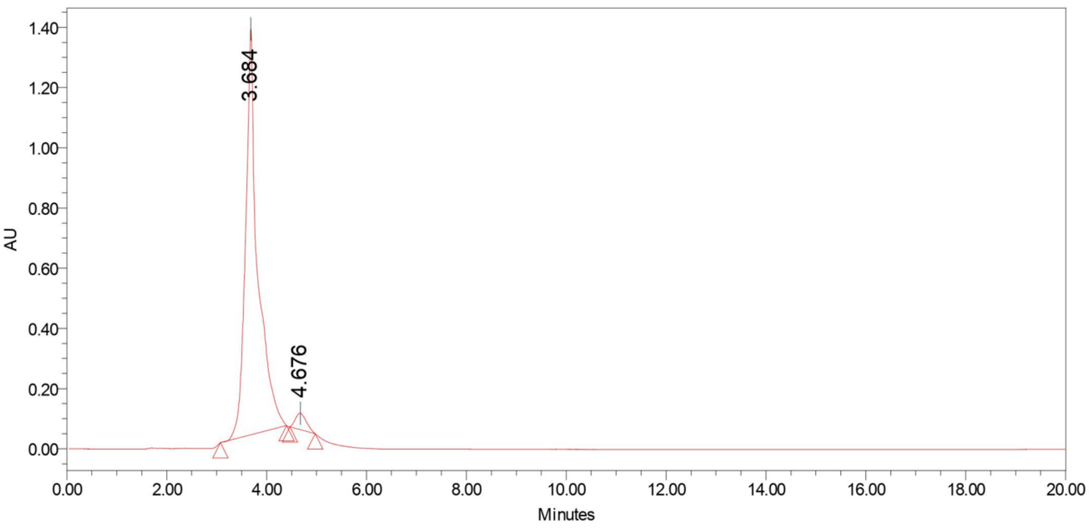

Peak Results

	Name	RT	Area	Height	Amount	Units	% Area
1		3.684	22929802	1348241			96.50
2		4.676	831495	54382			3.50

Figure 1. UPLC of **3a**

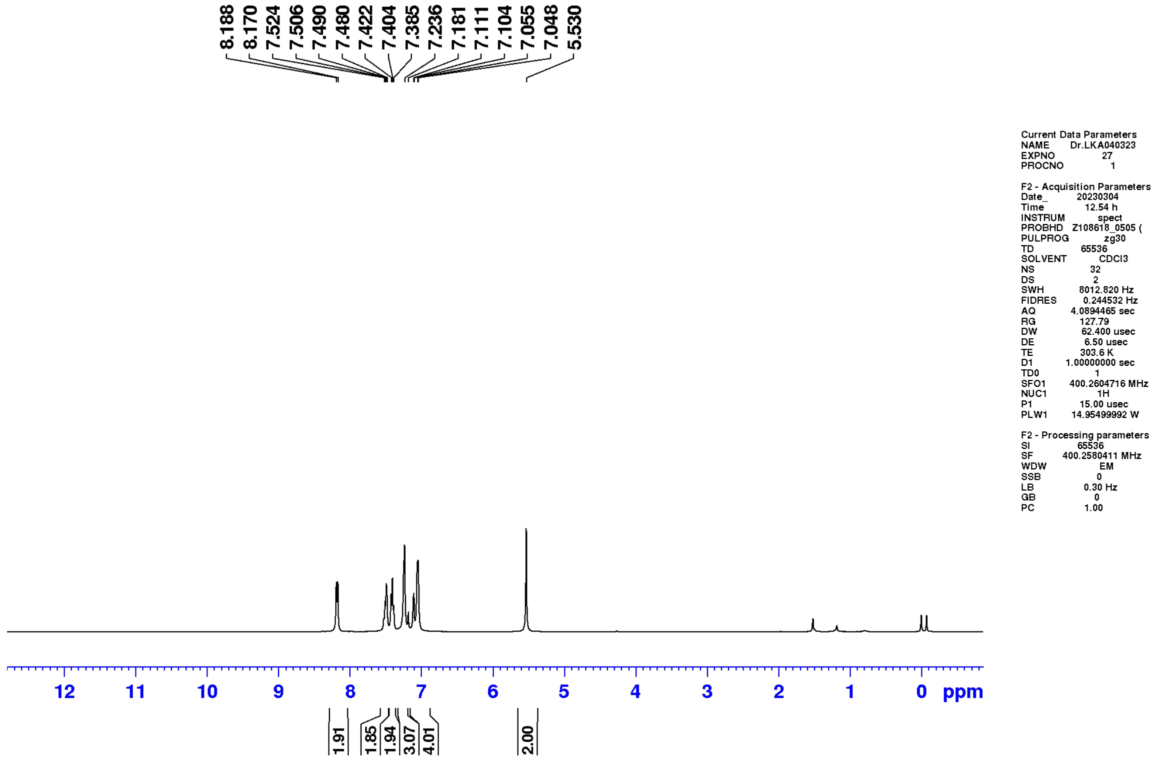

Figure S2. ^{1}H NMR of **3b**

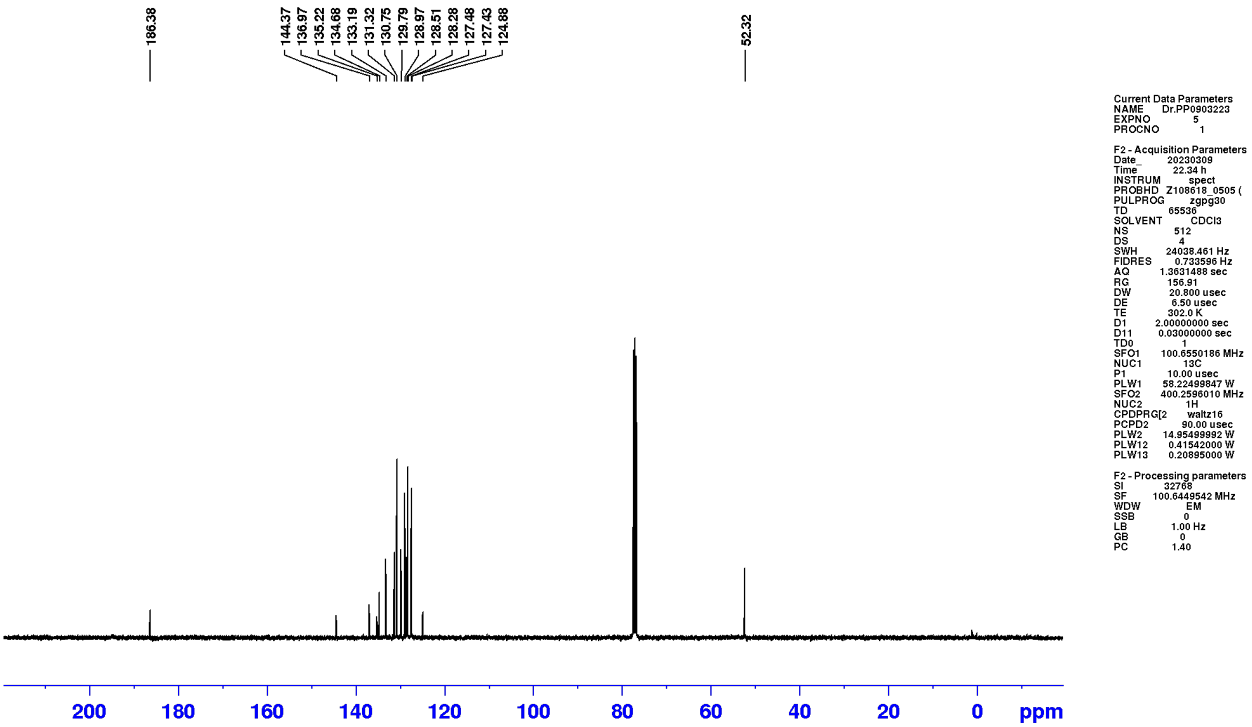

Figure S7. ^{13}H NMR of **3b**

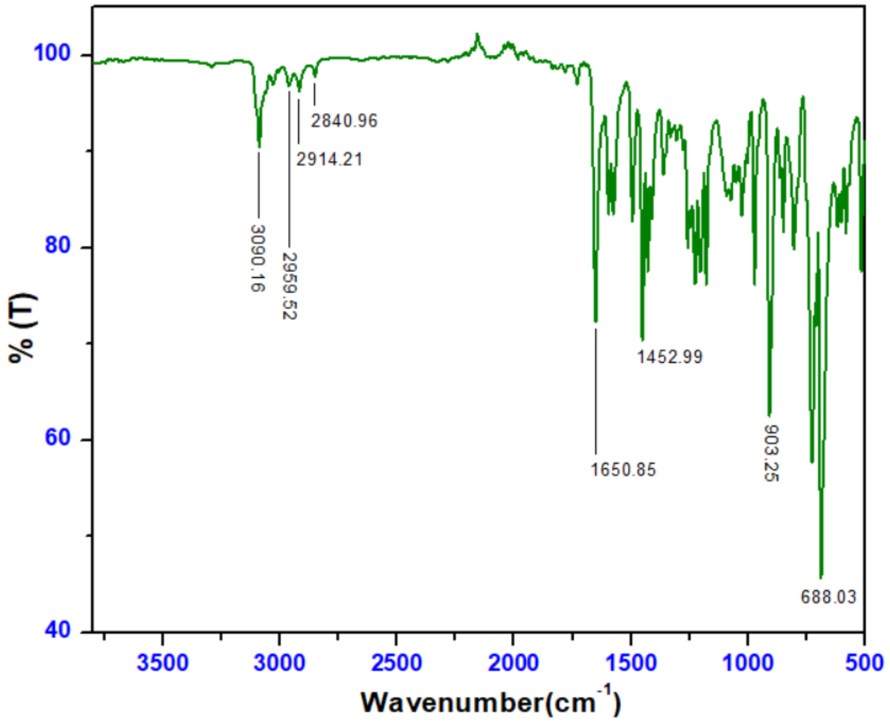

Figure S3. IR spectrum of **3b**

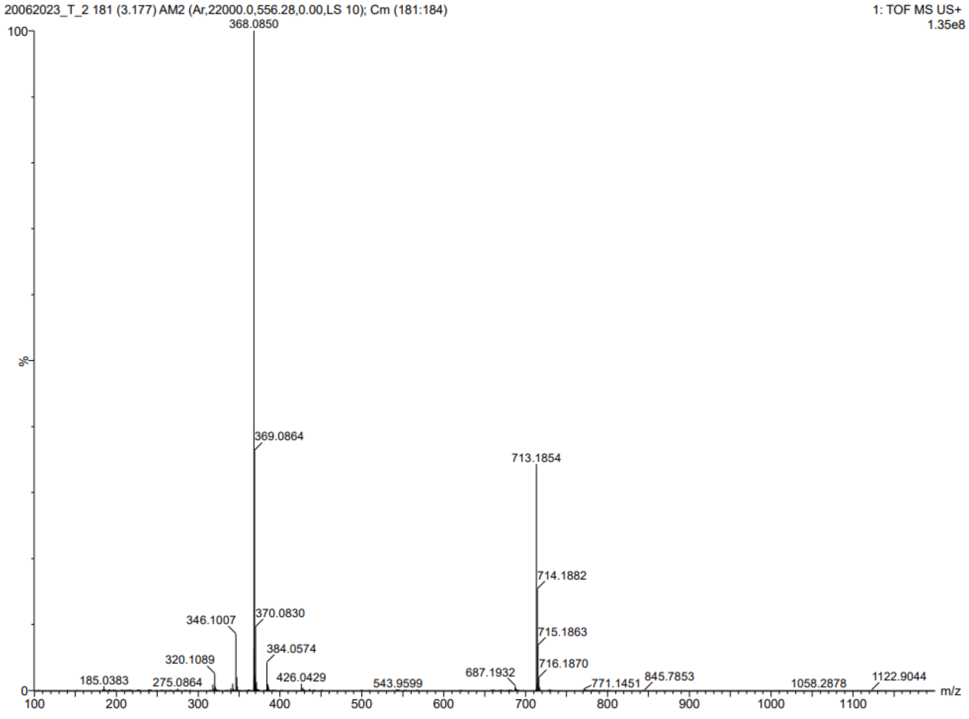

Figure S4. ESI HRMS of **3b**

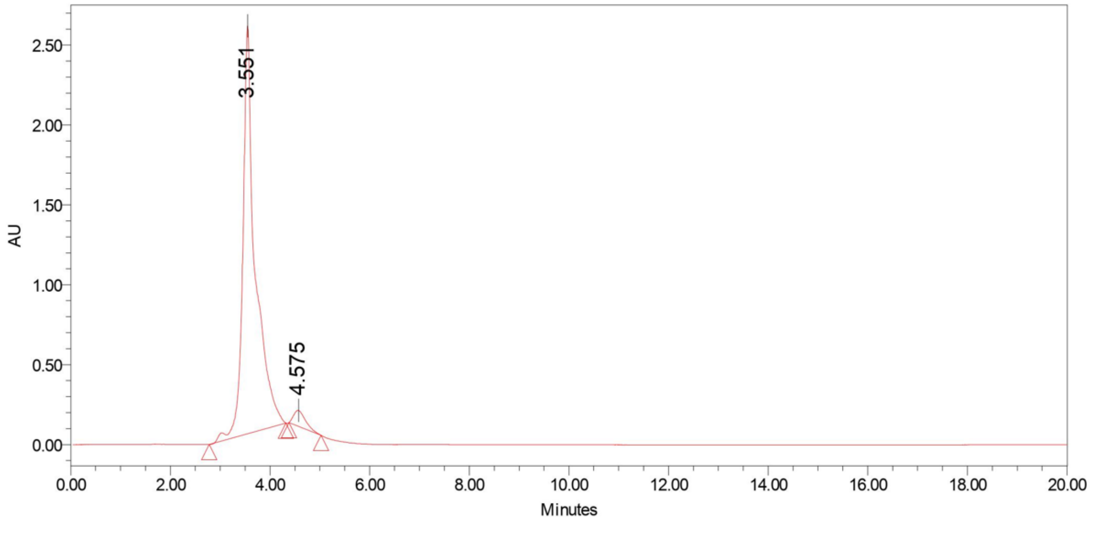

Peak Results

	Name	RT	Area	Height	Amount	Units	% Area
1		3.551	44132564	2550866			96.33
2		4.575	1680056	100337			3.67

Figure S5. UPLC of **3b**

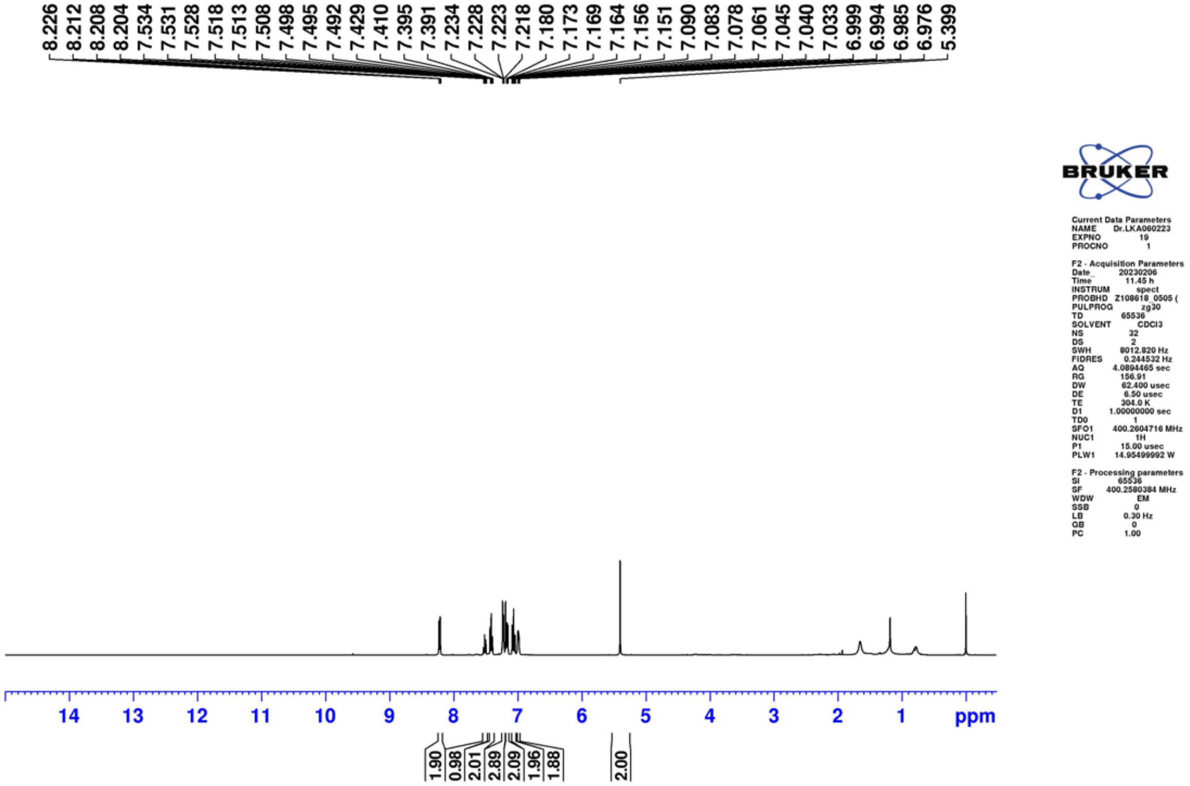

Figure S16. ^{1}H NMR of **3c**

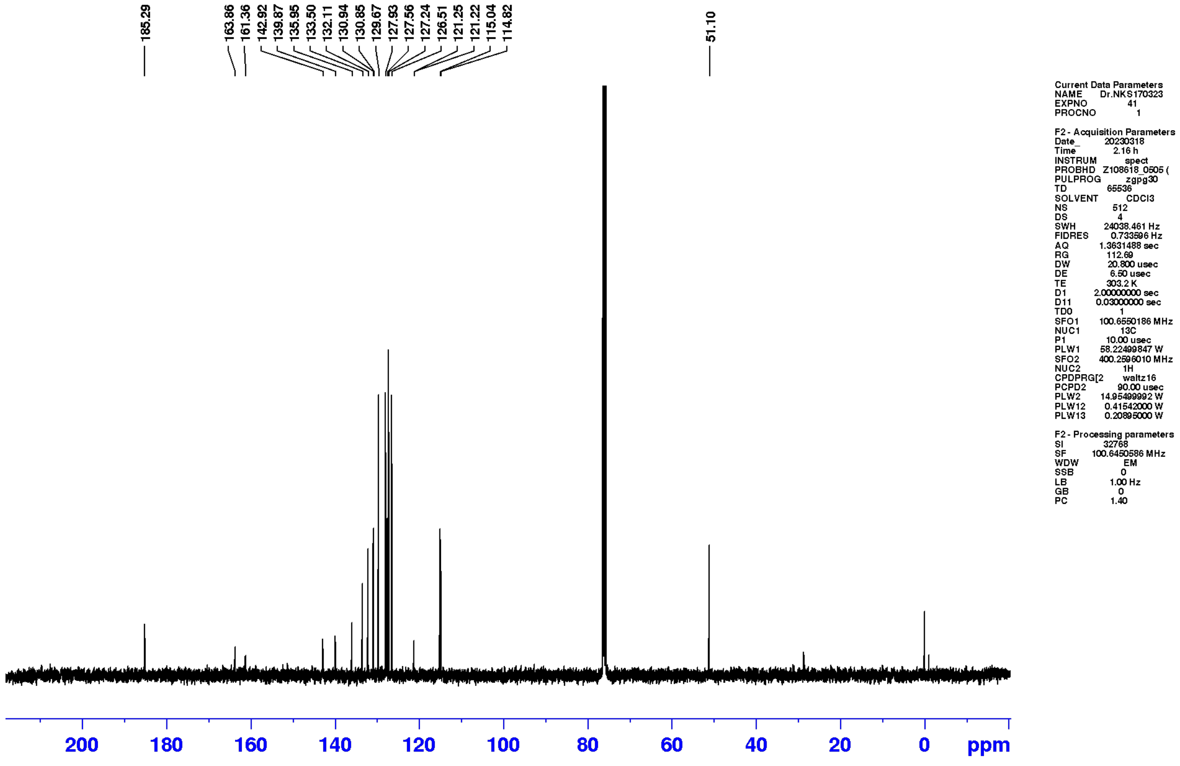

Figure S17. ^{13}H NMR of **3c**

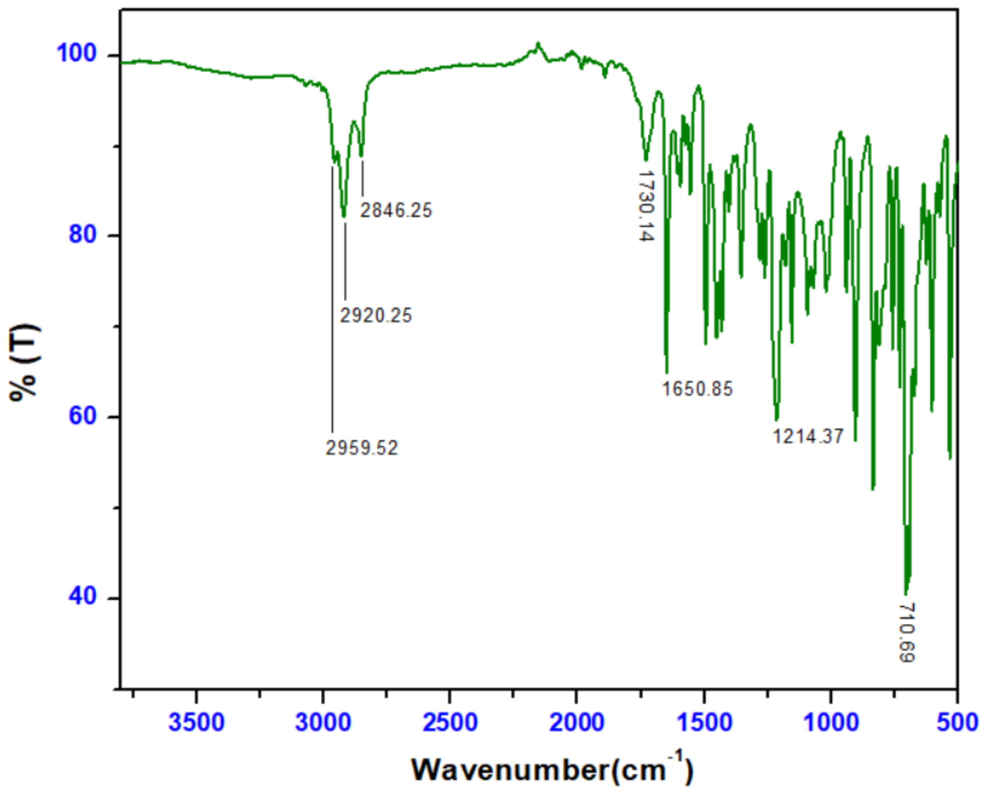

Figure S18. IR spectrum of **3c**

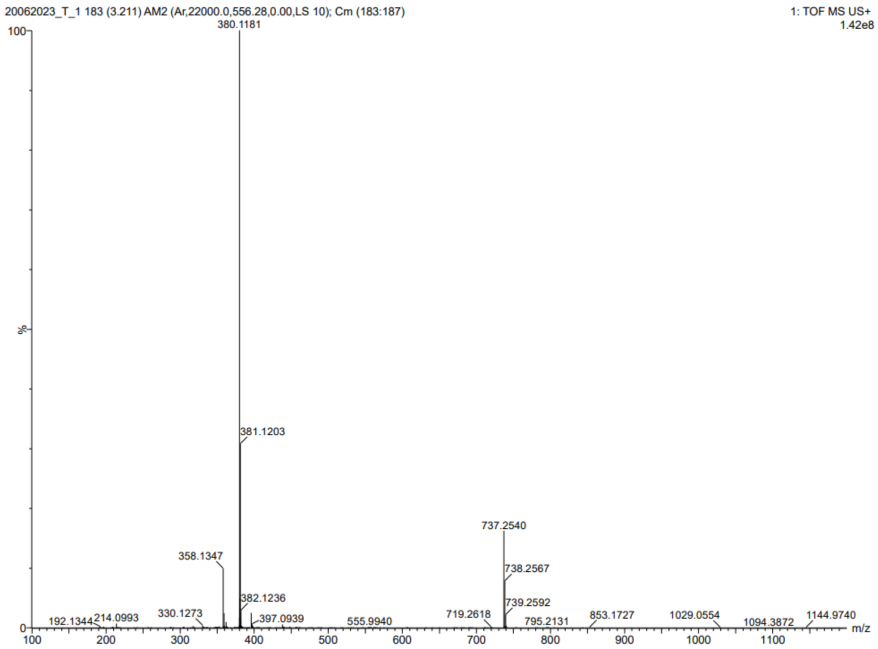

Figure S19. ESI HRMS of **3c**

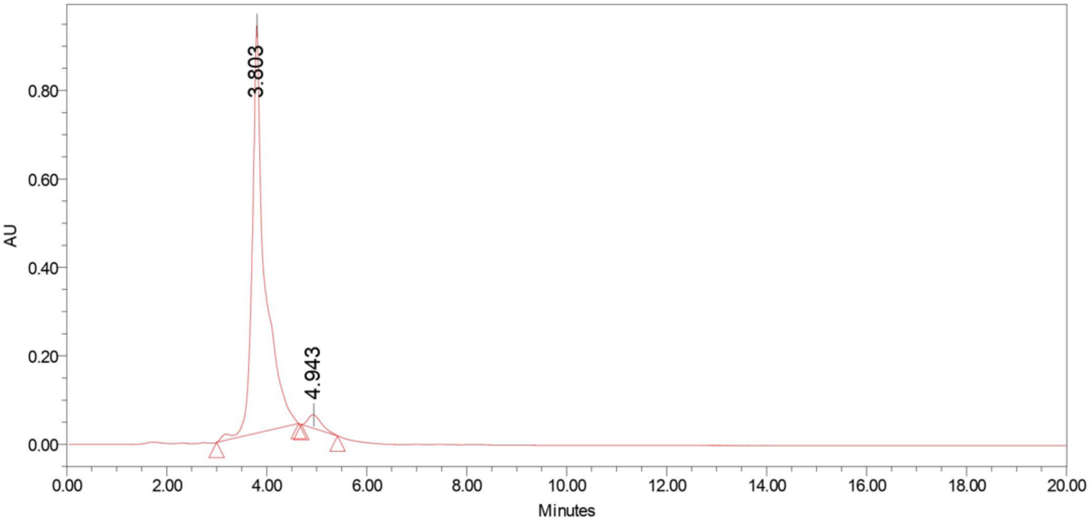

Peak Results

	Name	RT	Area	Height	Amount	Units	% Area
1		3.803	16063696	921891			96.56
2		4.943	572142	30512			3.44

Figure S110. UPLC of **3c**

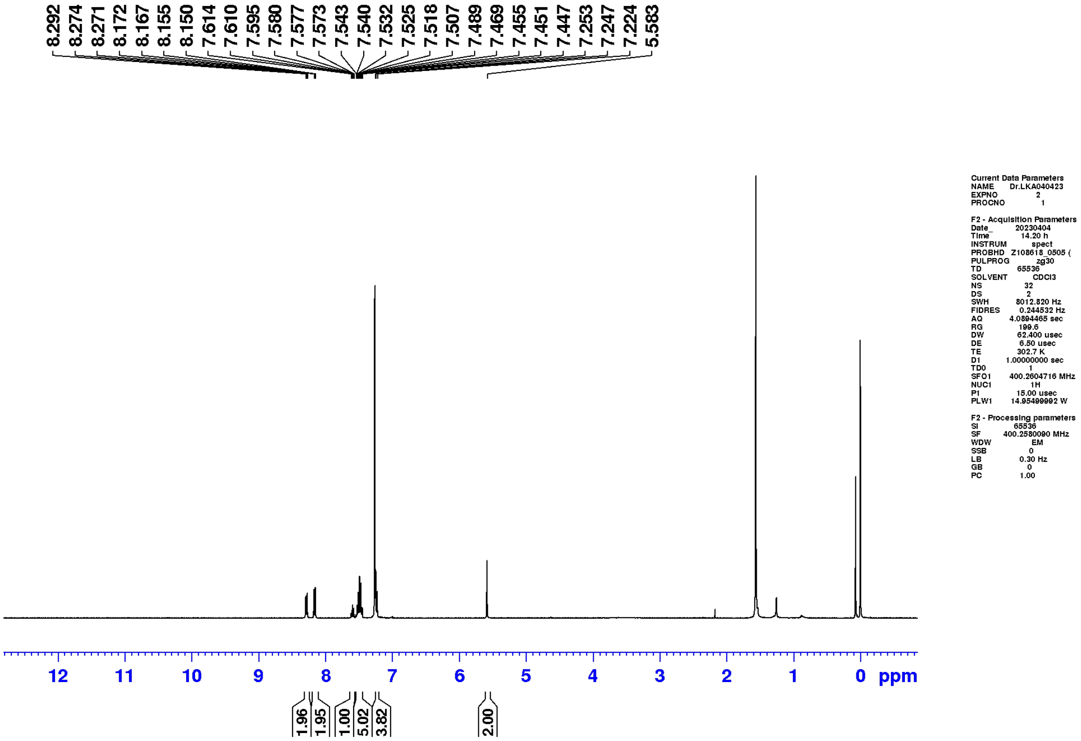

Figure S11. ^{1}H NMR of **3d**

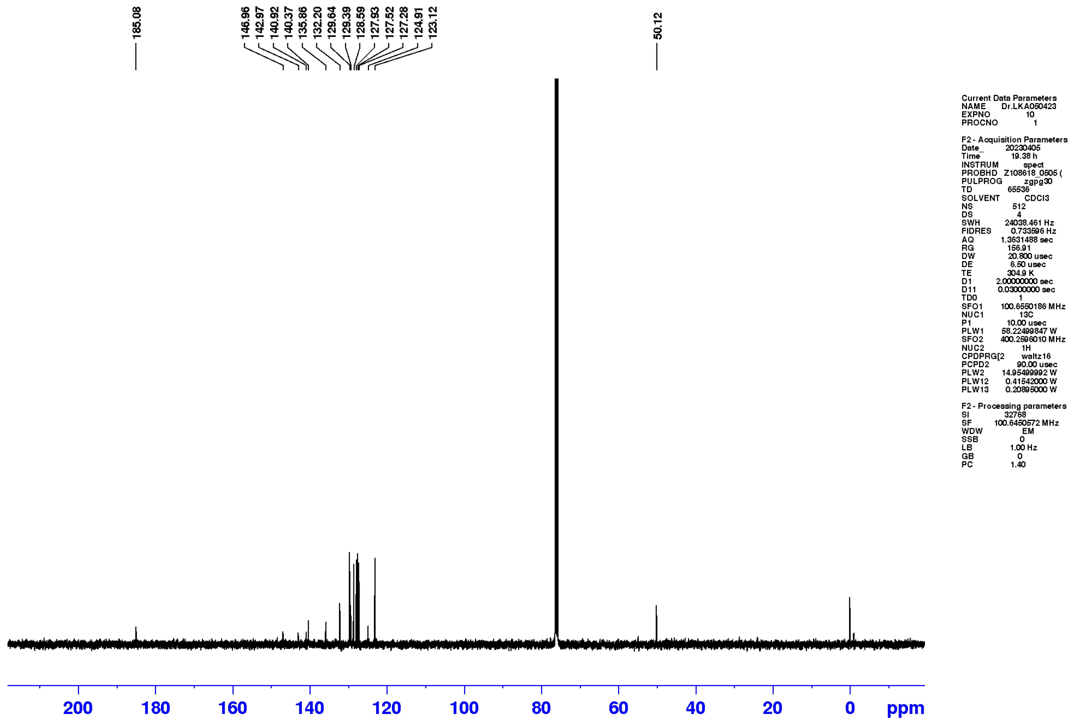

Figure S12. ^{13}H NMR of **3d**

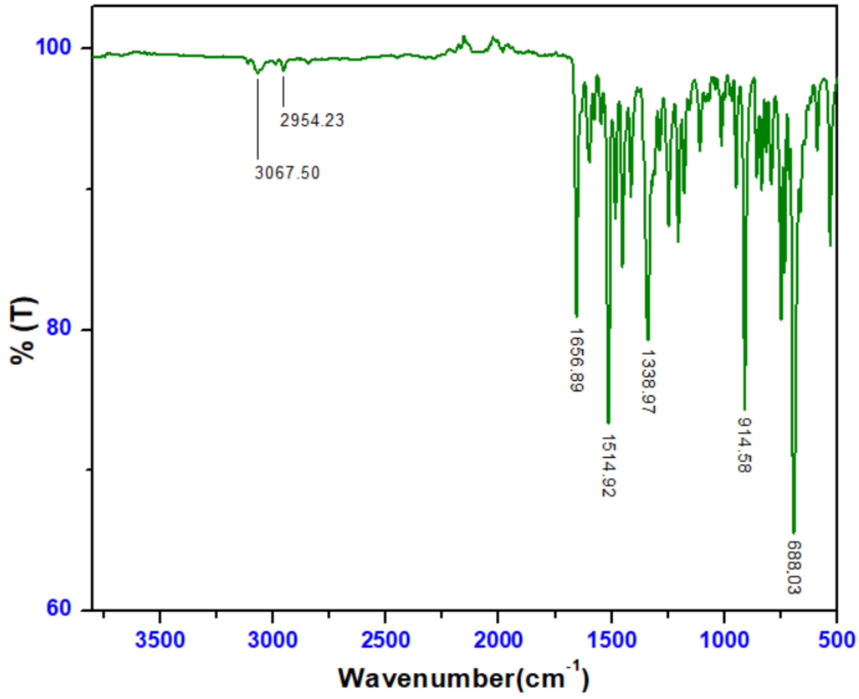

Figure S13. IR spectrum of **3d**

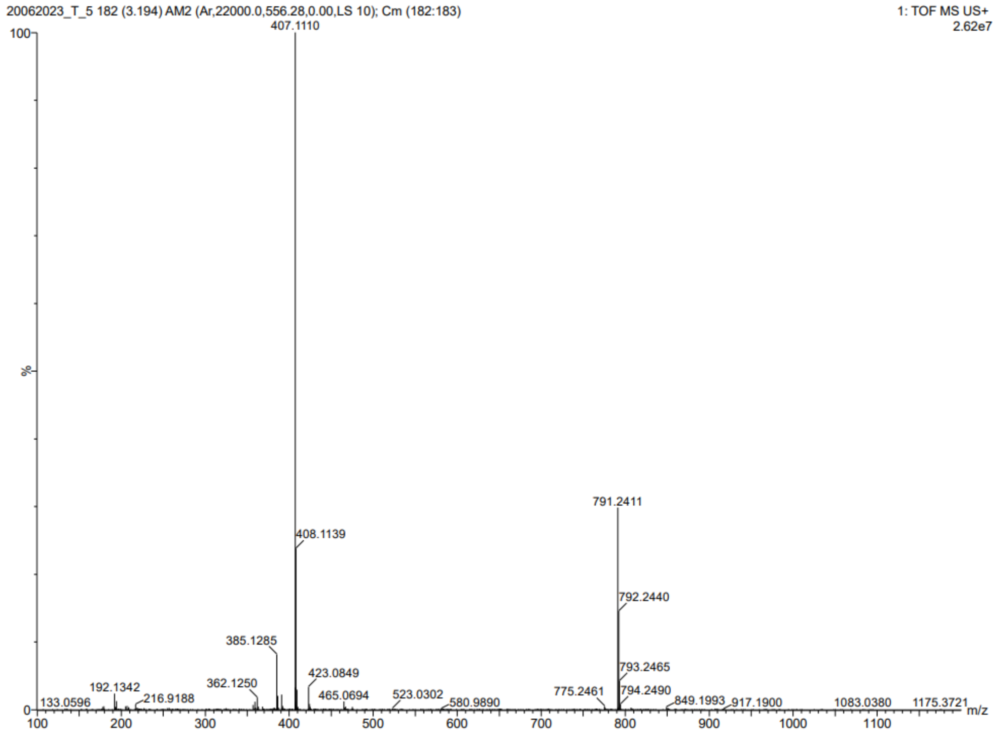

Figure S14. ESI HRMS of **3d**

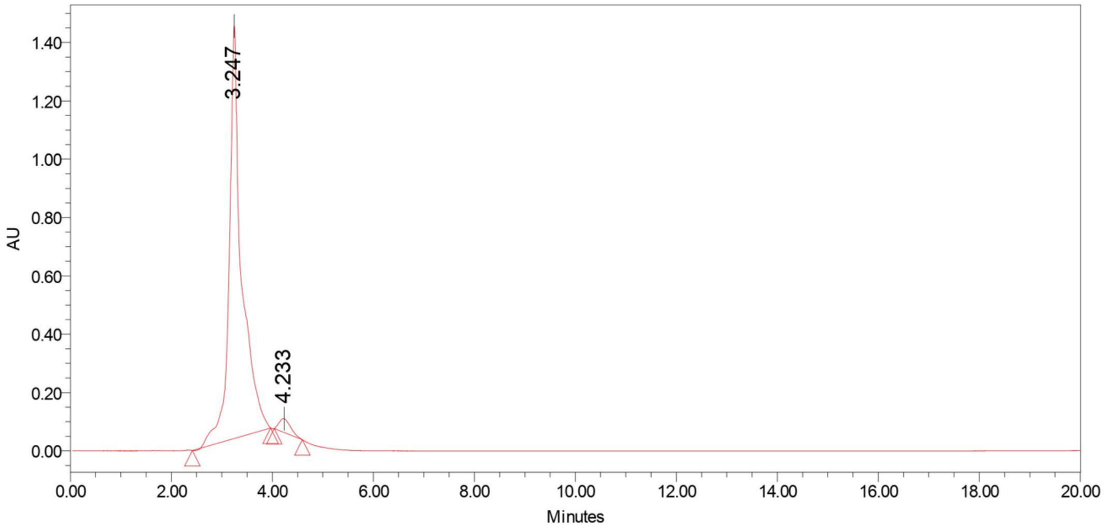

Peak Results

	Name	RT	Area	Height	Amount	Units	% Area
1		3.247	24183813	1412980			97.03
2		4.233	740990	47170			2.97

Figure S15. UPLC of **3d**

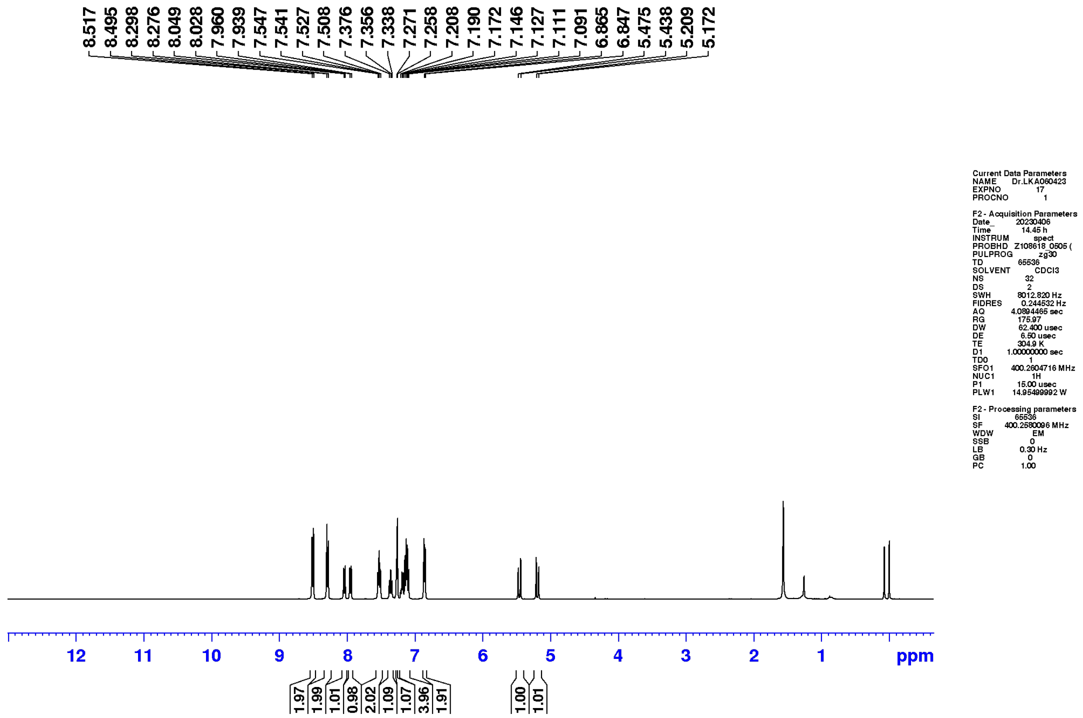

Figure S16. ^{1}H NMR of **3e**

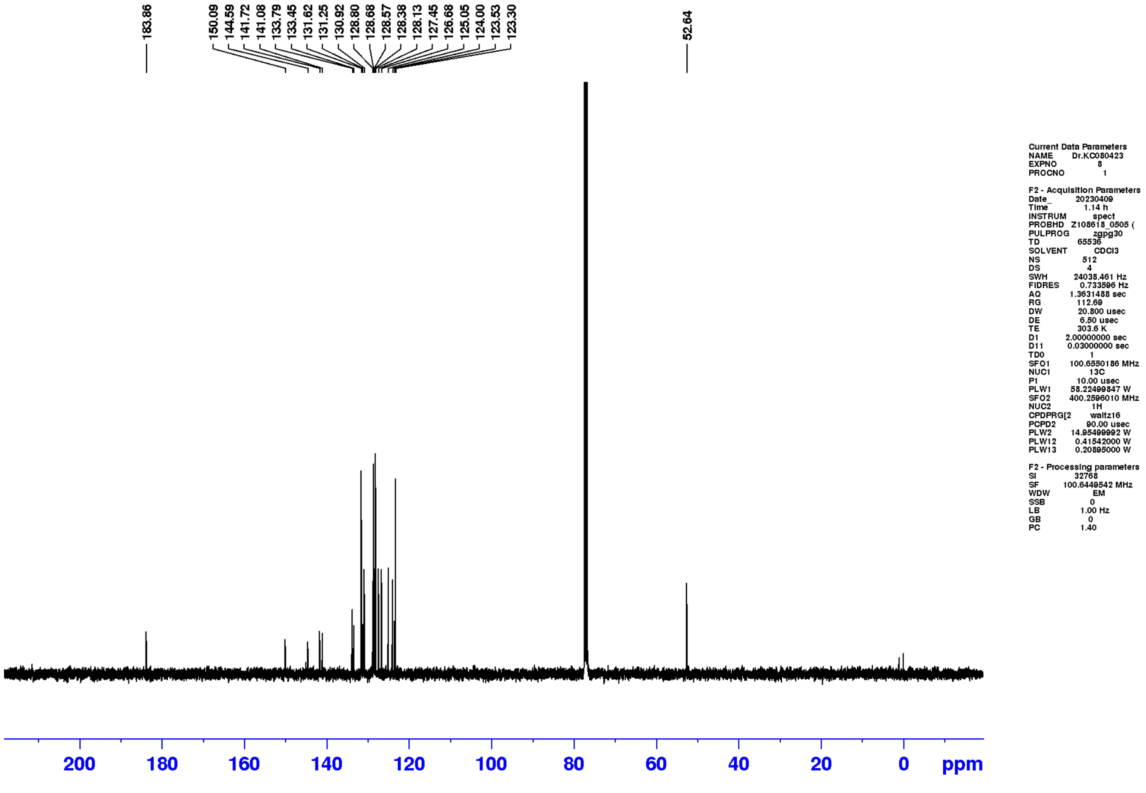

Figure S17. ^{13}C NMR of **3e**

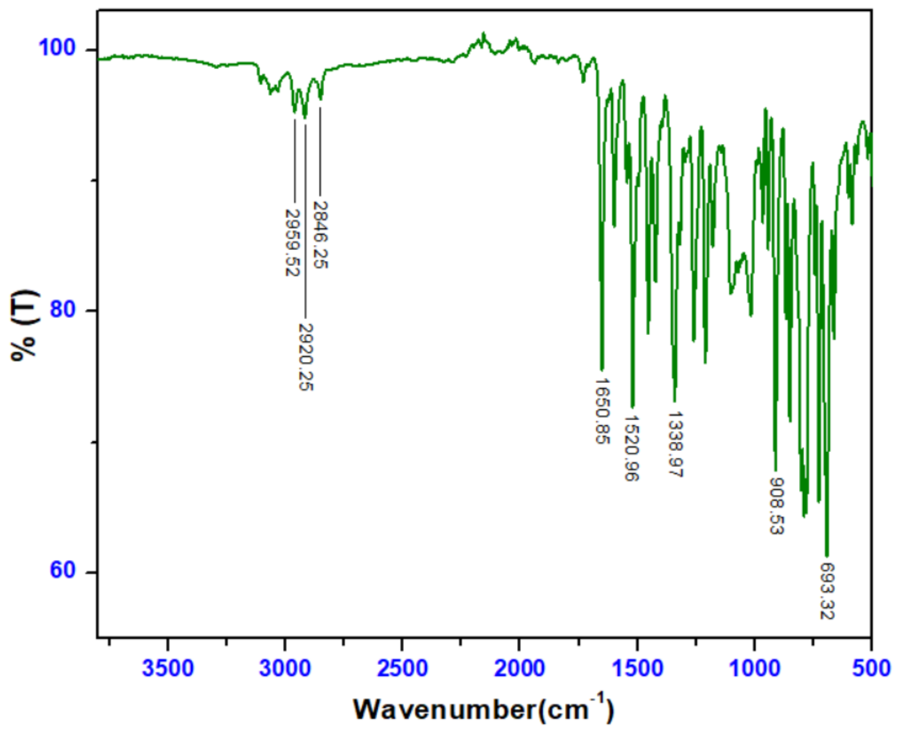

Figure S18. IR spectrum of **3e**

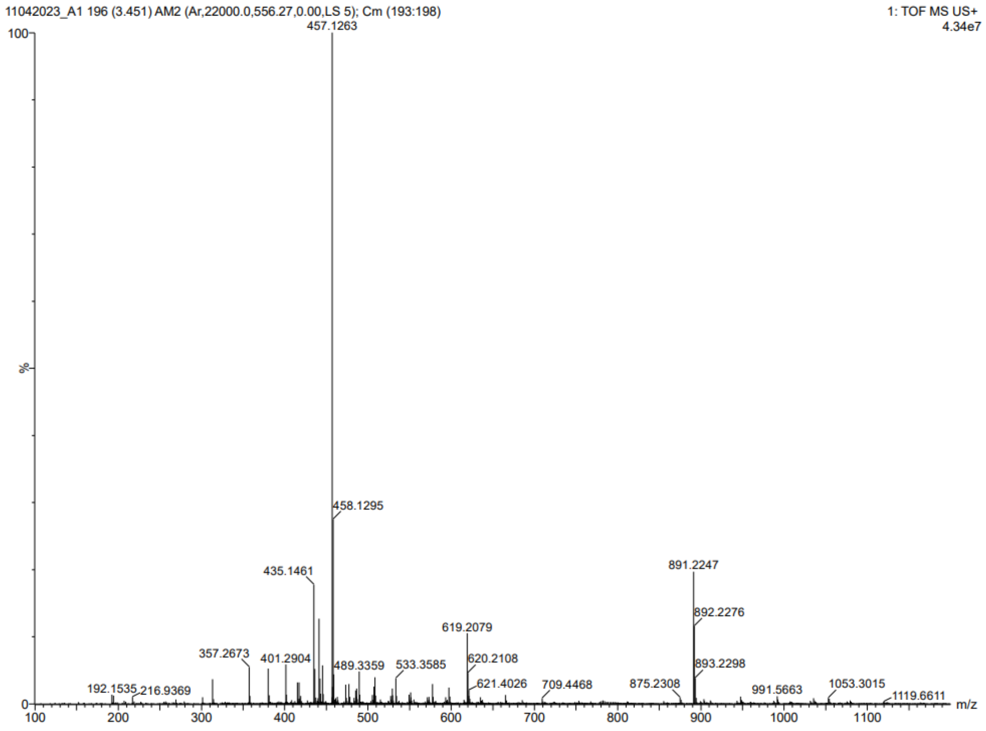

Figure S19. ESI HRMS of **3e**

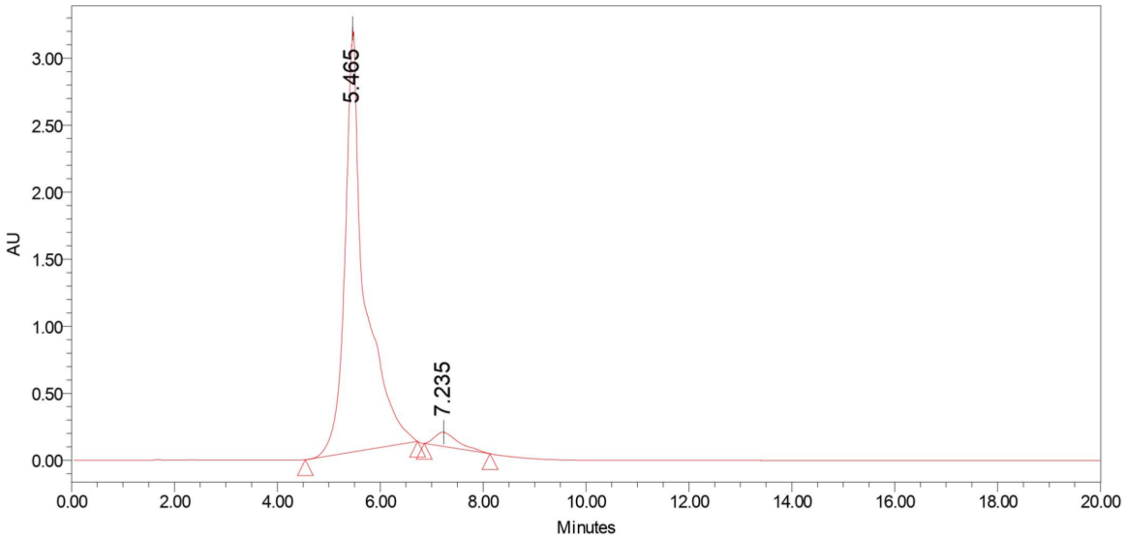

Peak Results

	Name	RT	Area	Height	Amount	Units	% Area
1		5.465	84373473	3166173			96.15
2		7.235	3375297	105439			3.85

Figure S20. UPLC of **3e**

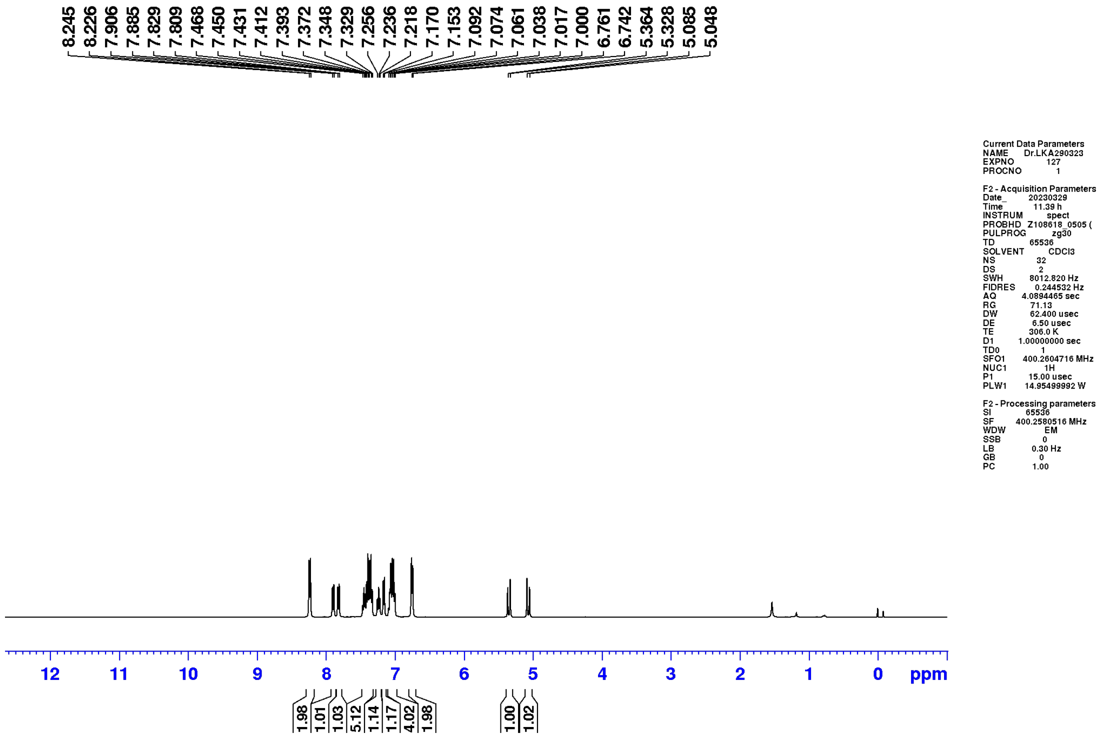

Figure S21. ^{1}H NMR of **3f**

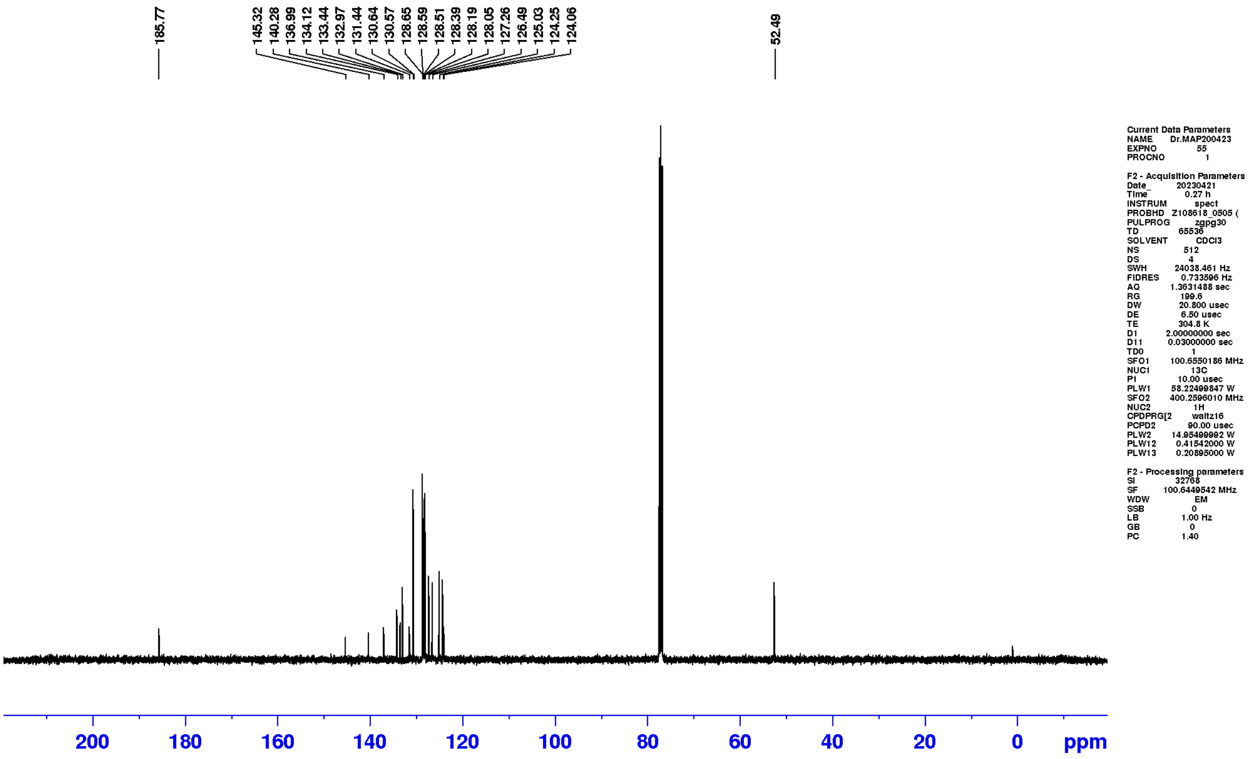

Figure S22. ^{13}H NMR of **3f**

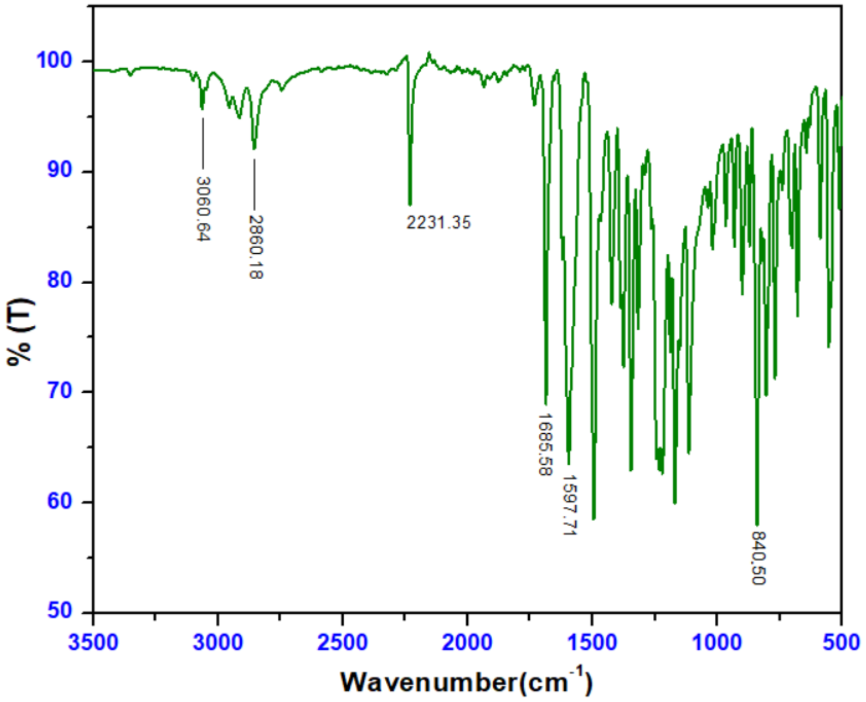

Figure S23. IR spectrum of **3f**

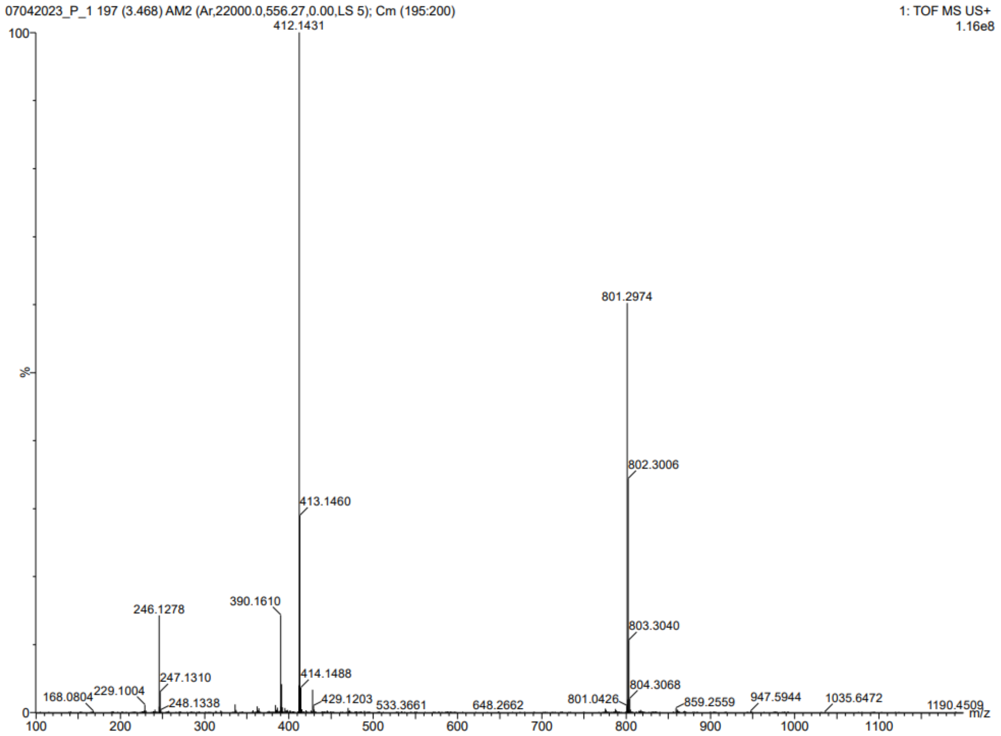

Figure S24. ESI HRMS of **3f**

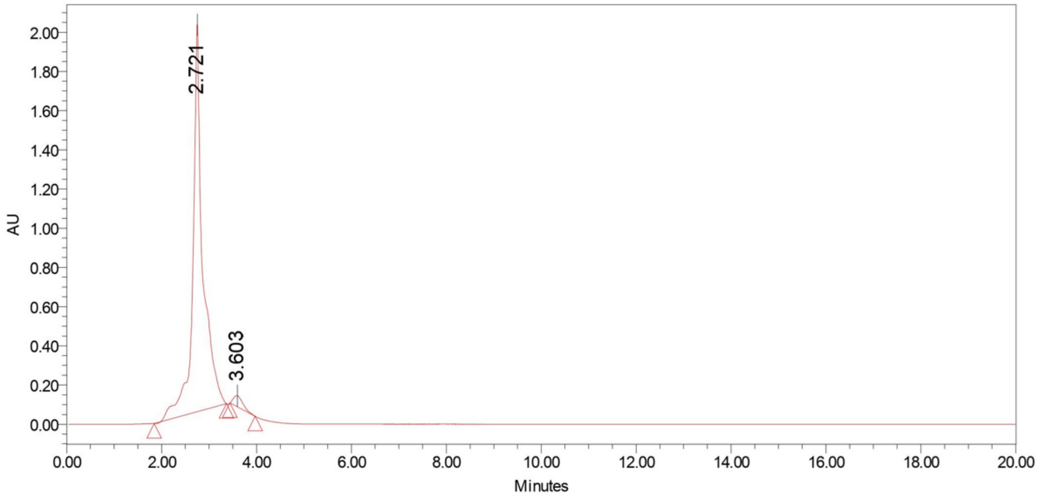

Peak Results

	Name	RT	Area	Height	Amount	Units	% Area
1		2.721	28846427	1974554			97.36
2		3.603	781268	56916			2.64

Figure S25. UPLC of **3f**

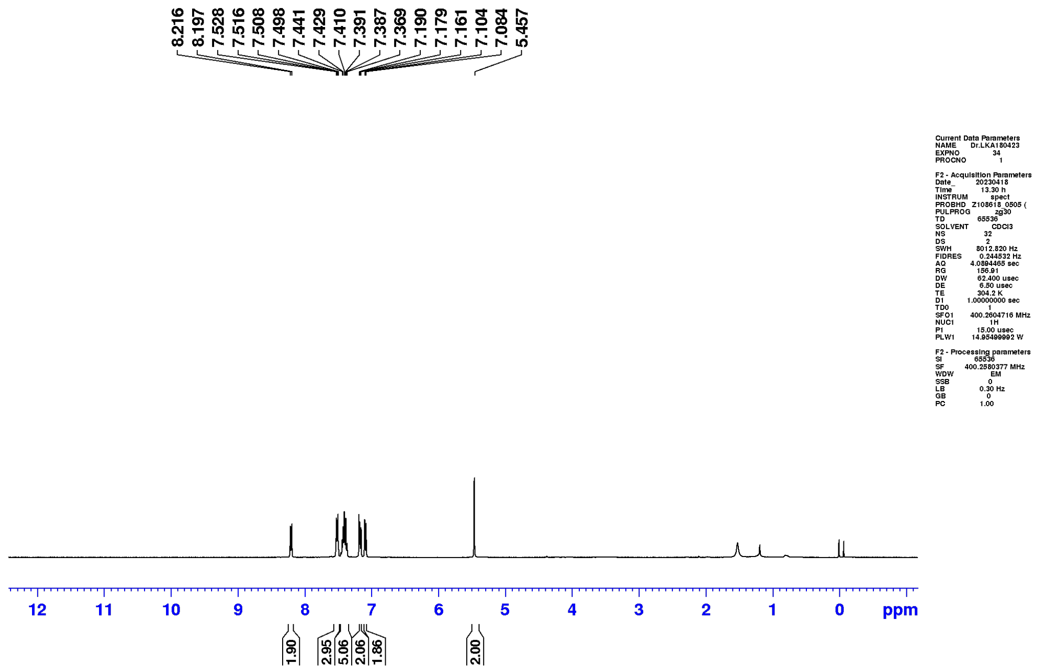

Figure S26. ^{1}H NMR of **3g**

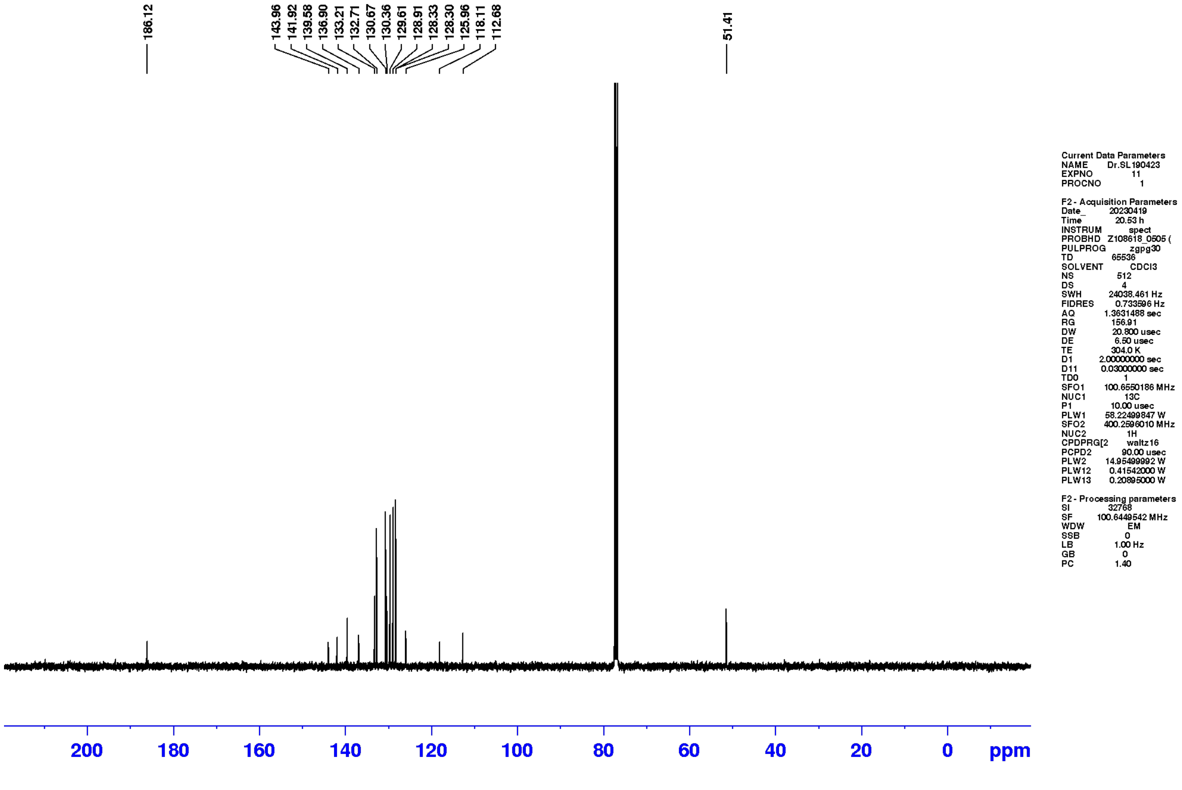

Figure S27. ^{13}H NMR of **3g**

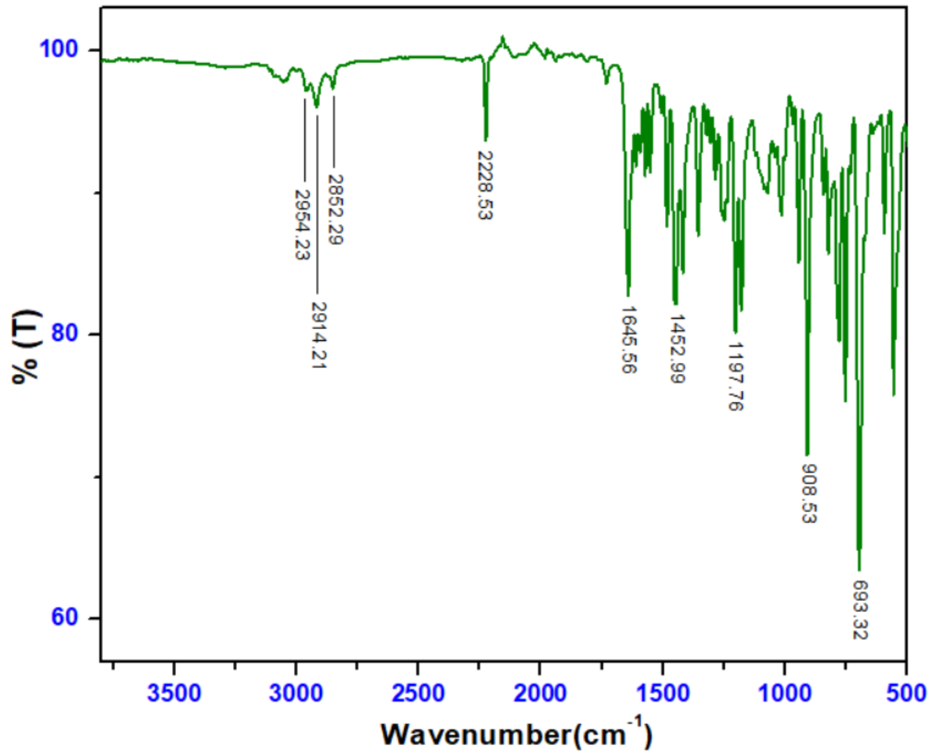

Figure S28. IR spectrum of **3g**

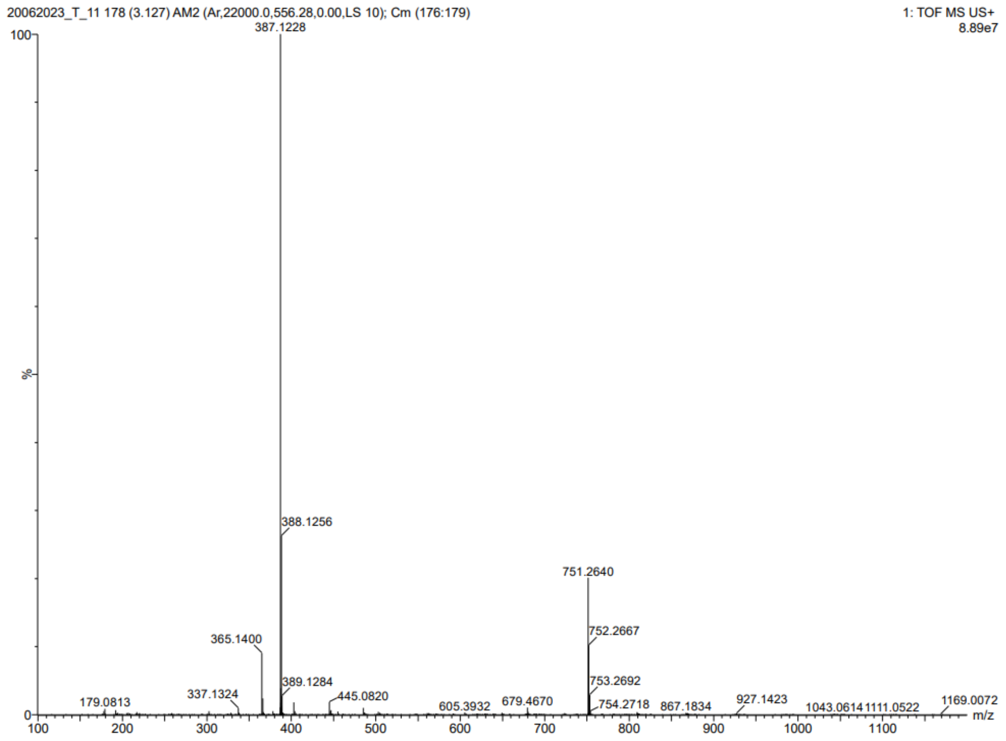

Figure S29. ESI HRMS of **3g**

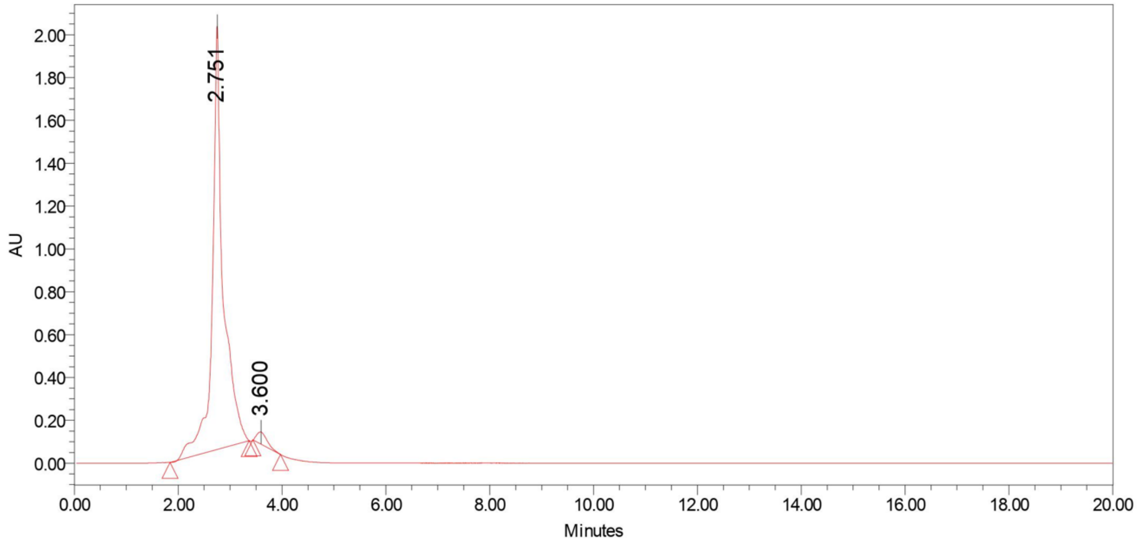

Peak Results

	Name	RT	Area	Height	Amount	Units	% Area
1		2.751	28846407	1974555			97.36
2		3.600	781168	56910			2.64

Figure S30. UPLC of **3g**

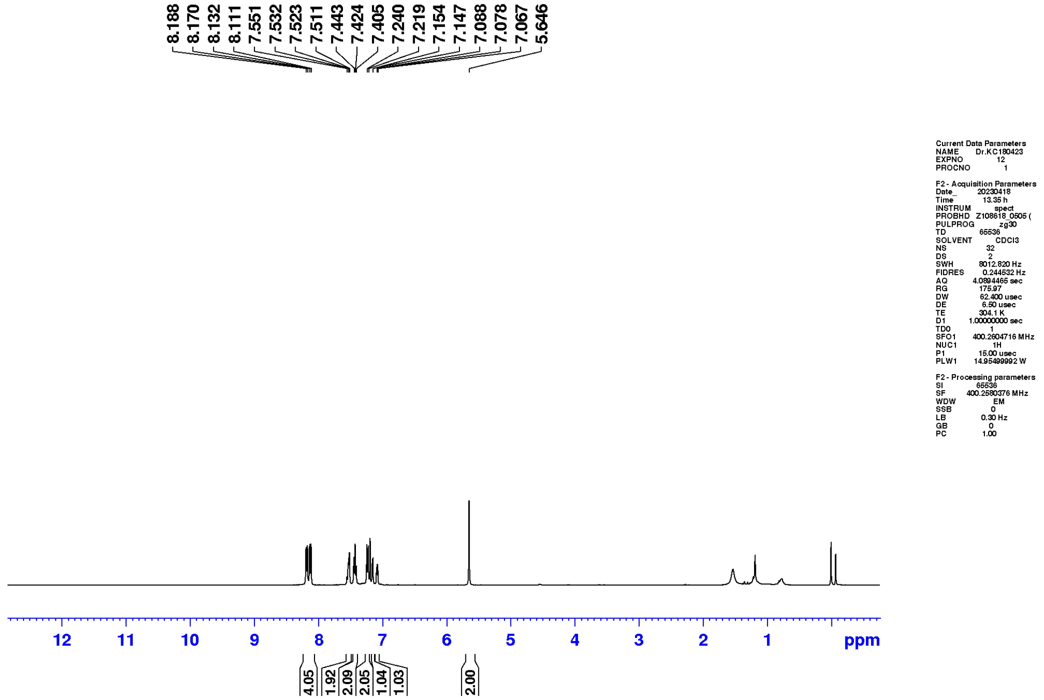

Figure S31. ^{1}H NMR of **3h**

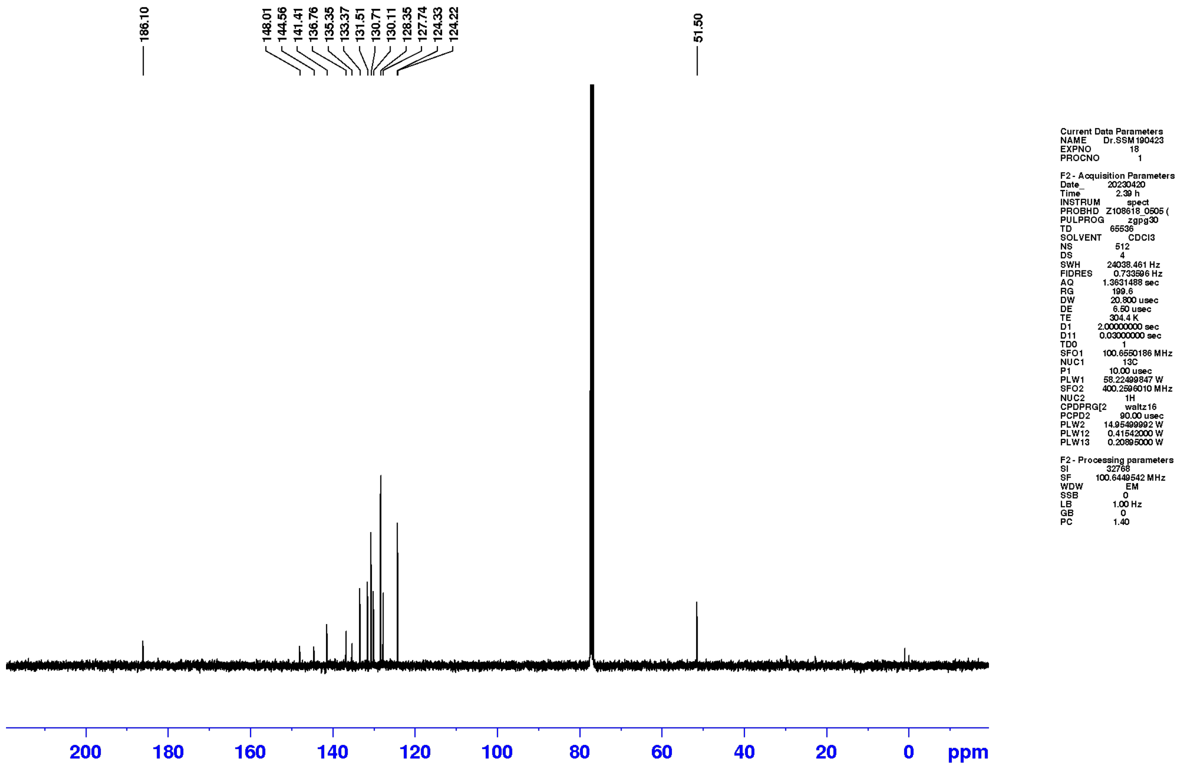

Figure S32. ^{13}H NMR of **3h**

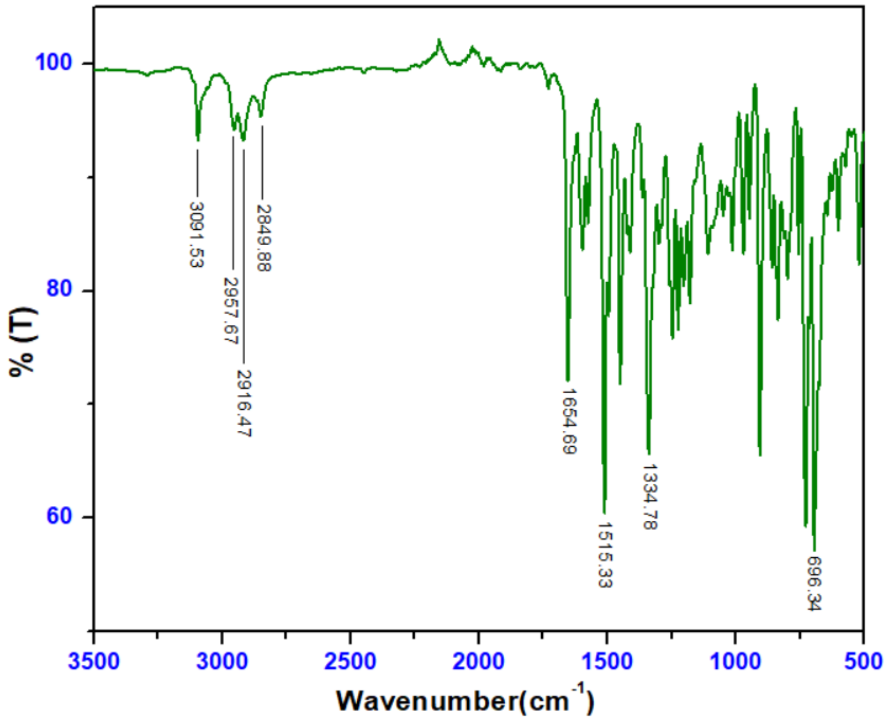

Figure S33. IR spectrum of **3h**

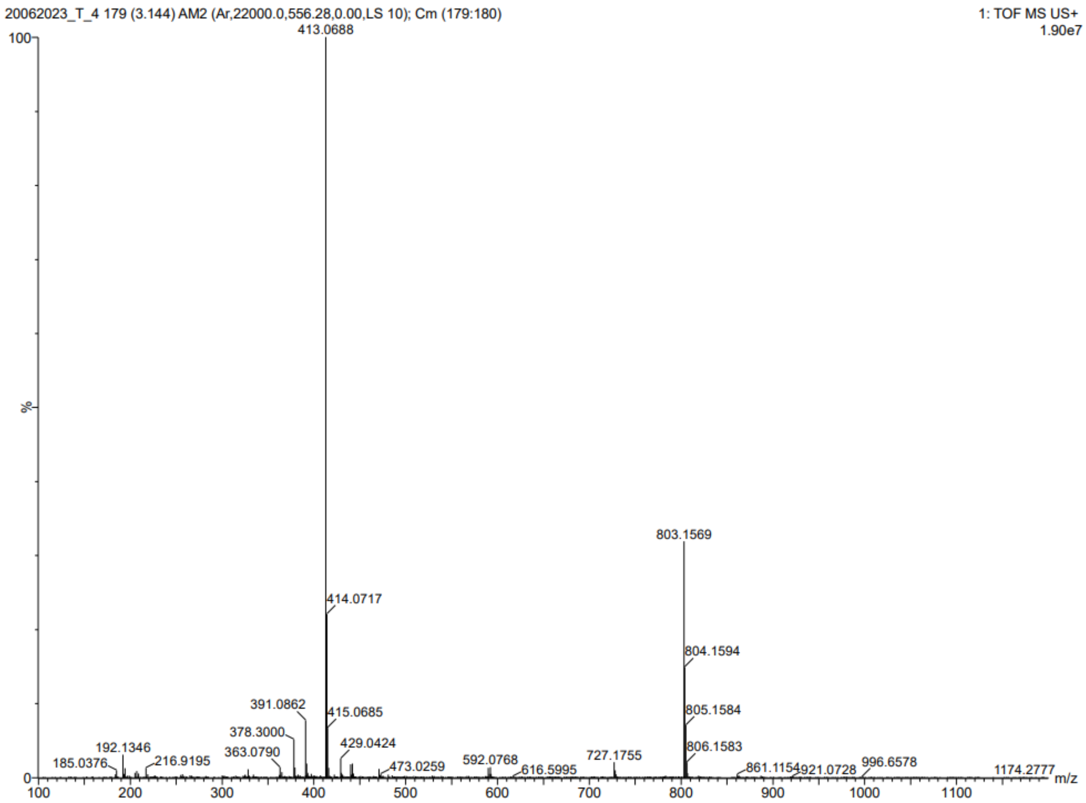

Figure S34. ESI HRMS of **3h**

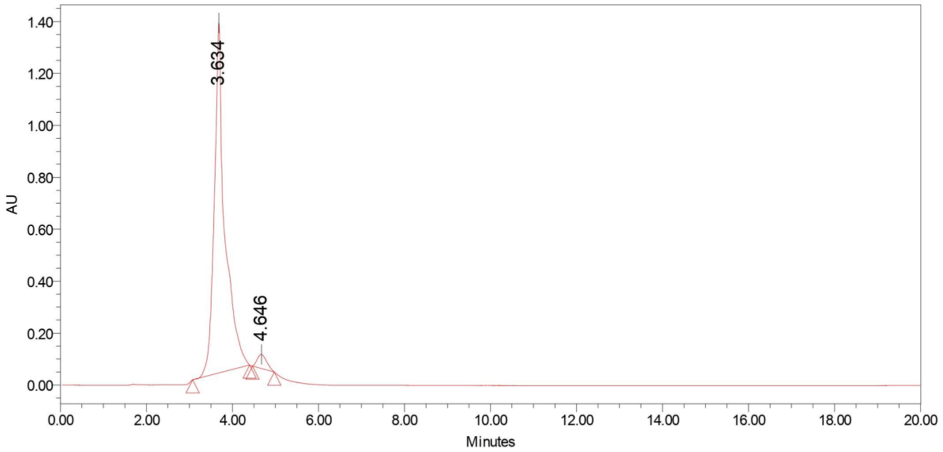

Peak Results

	Name	RT	Area	Height	Amount	Units	% Area
1		3.634	22829892	1348281			95.50
2		4.646	831495	54384			4.50

Figure S35. UPLC of **3h**

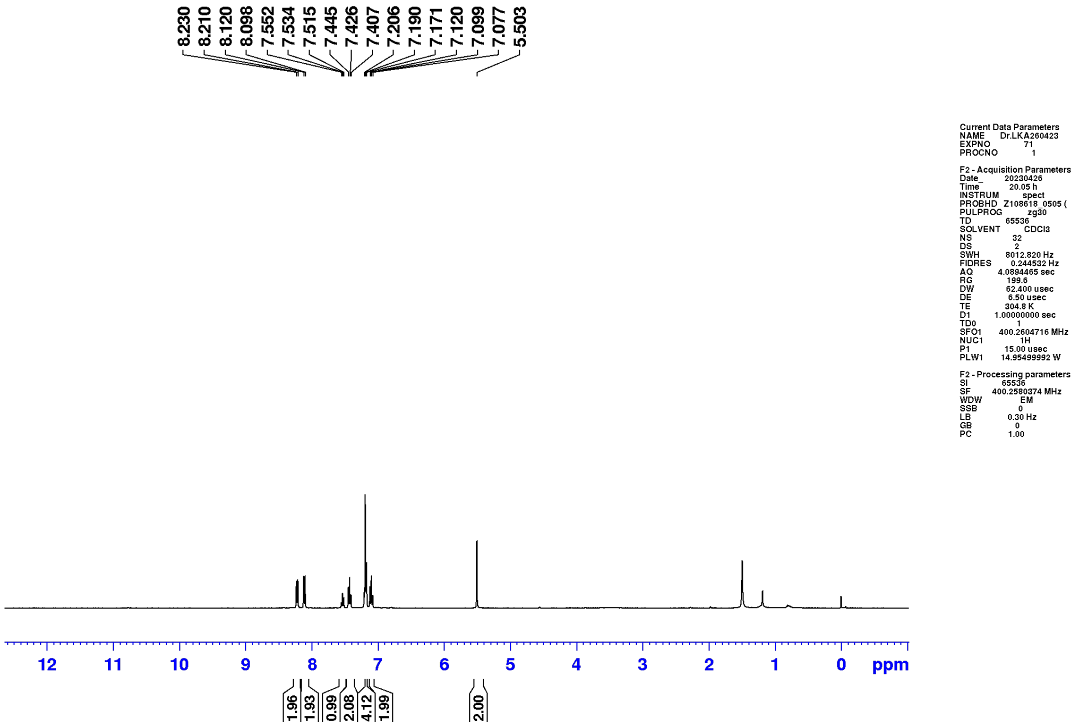

Figure S36. ^{1}H NMR of **3i**

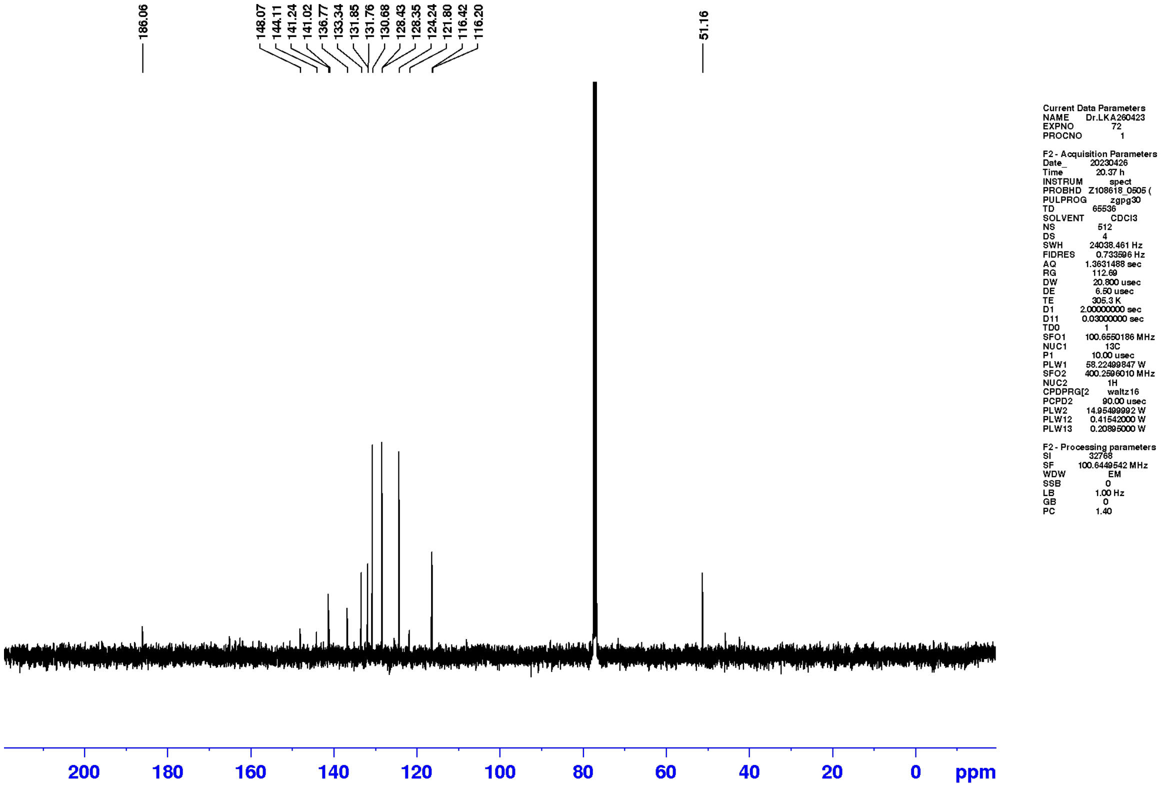

Figure S37. ^{13}H NMR of **3i**

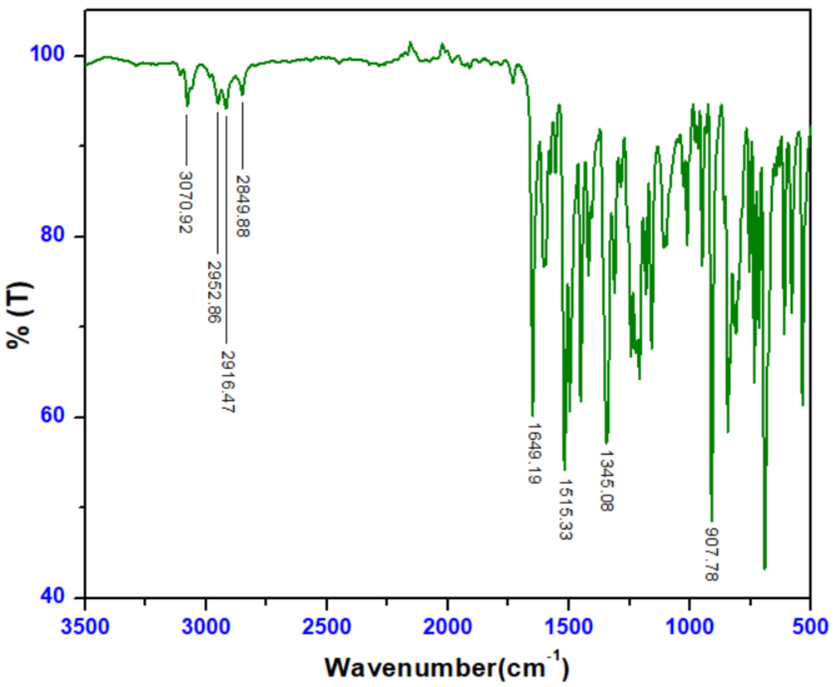

Figure S38. IR spectrum of **3i**

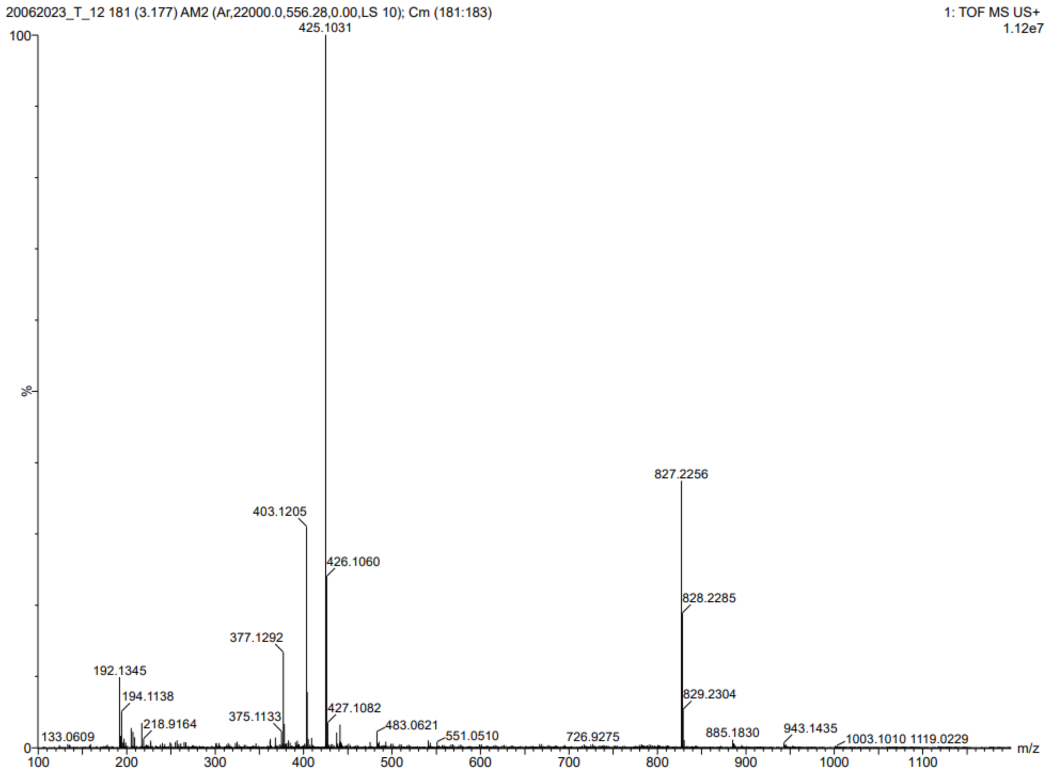

Figure S39. ESI HRMS of **3i**

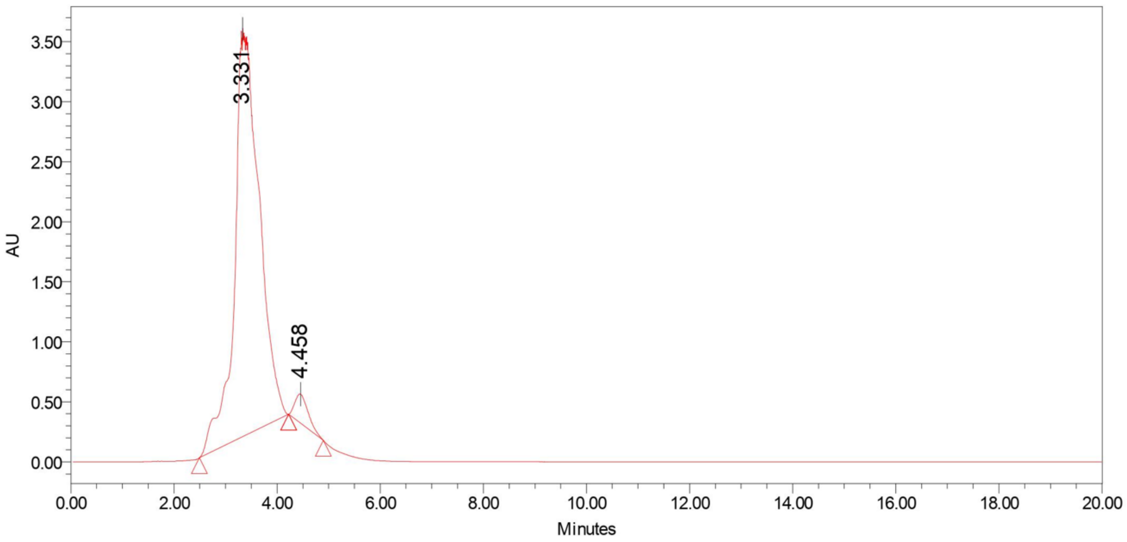

Peak Results

	Name	RT	Area	Height	Amount	Units	% Area
1		3.331	112650836	3394031			96.30
2		4.458	4328256	241783			3.70

Figure S40. UPLC of **3i**

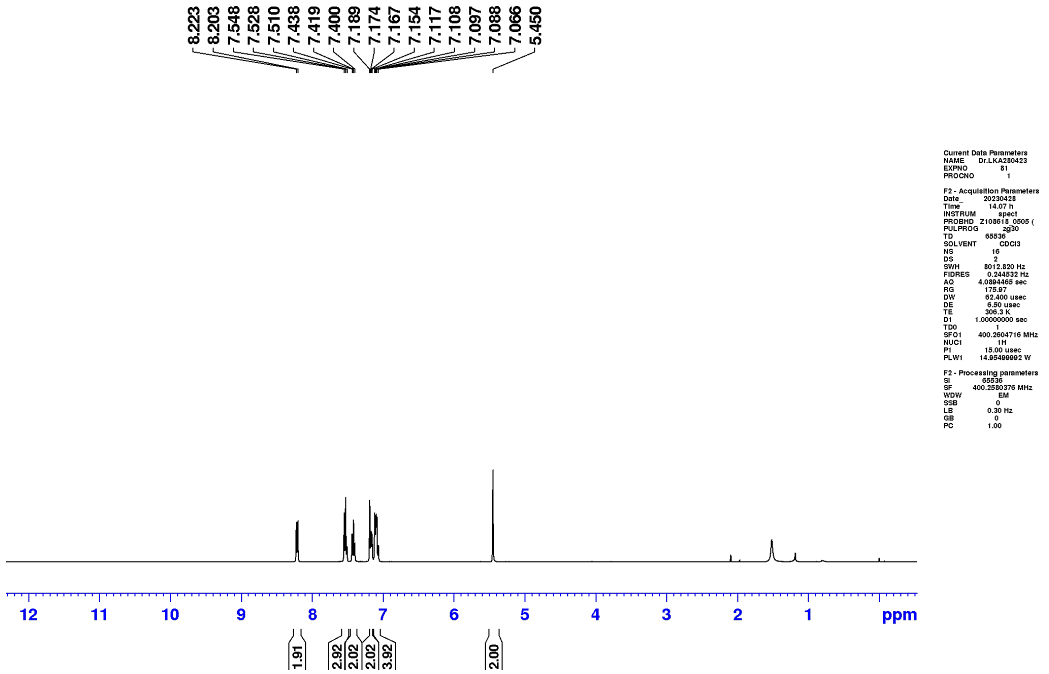

Figure S41. ^{1}H NMR of **3j**

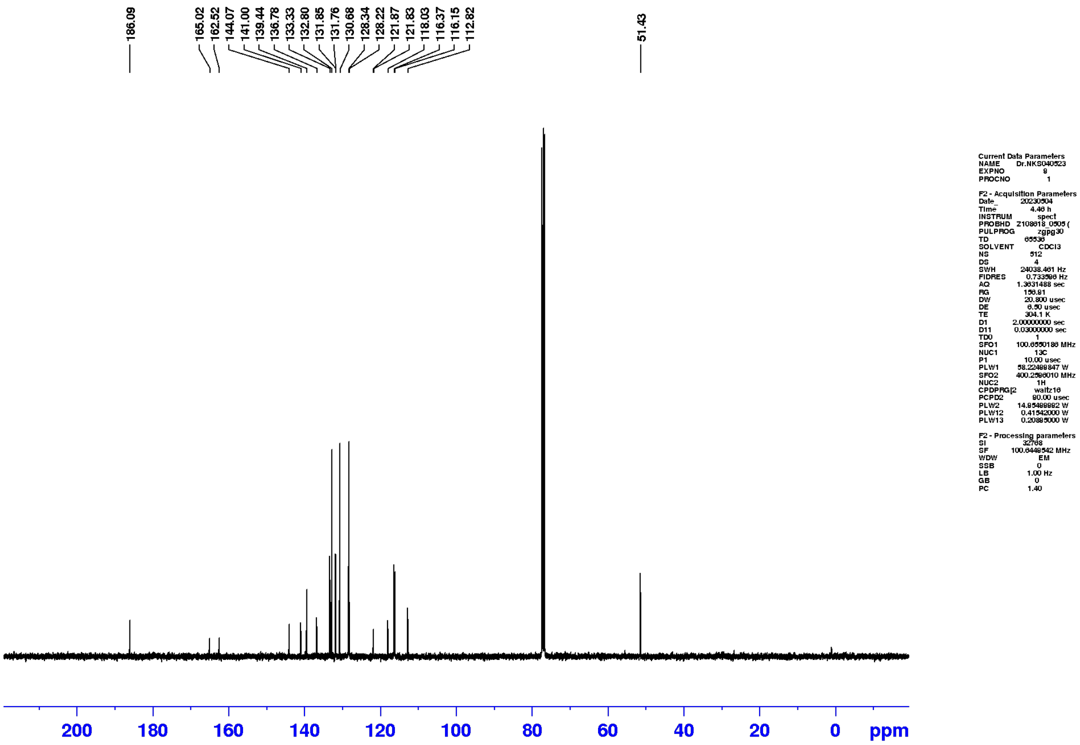

Figure S42. ^{13}H NMR of **3j**

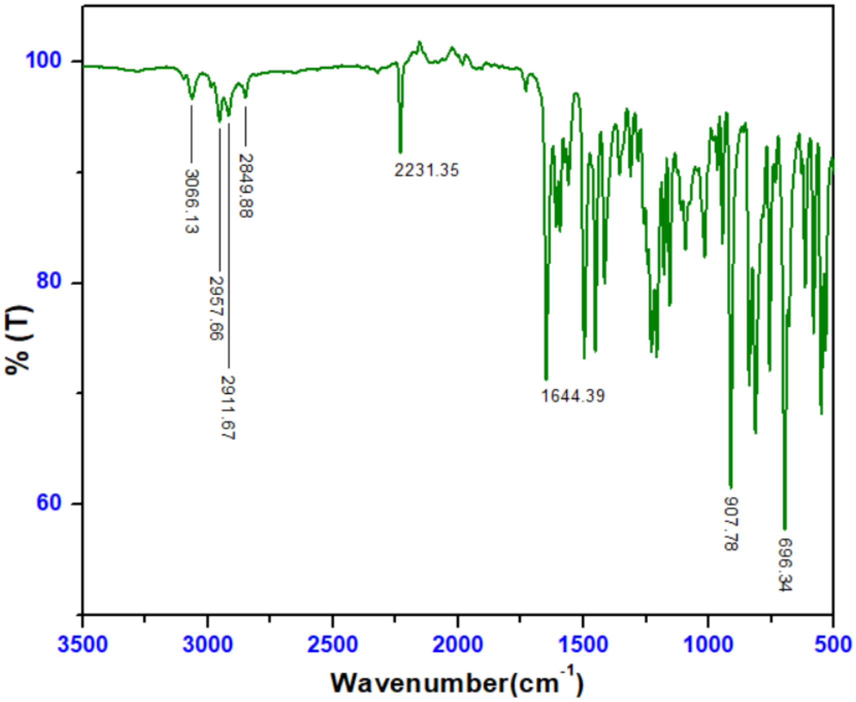

Figure S43. IR spectrum of **3j**

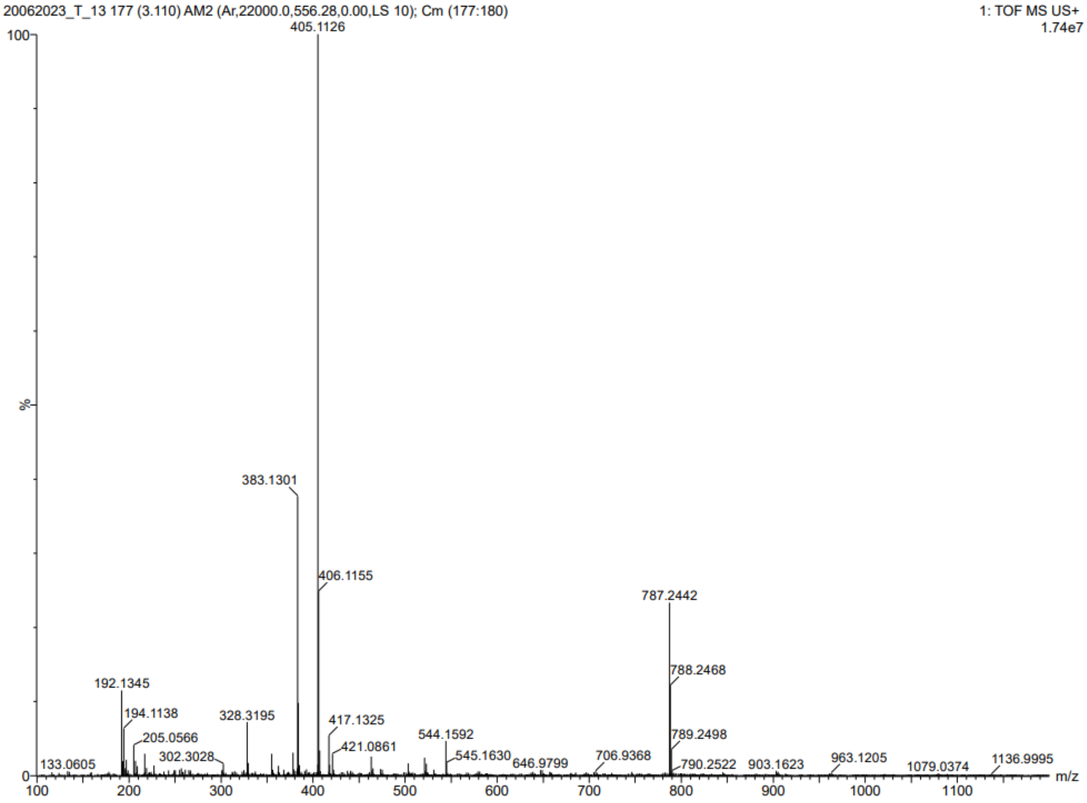

Figure S44. ESI HRMS of **3j**

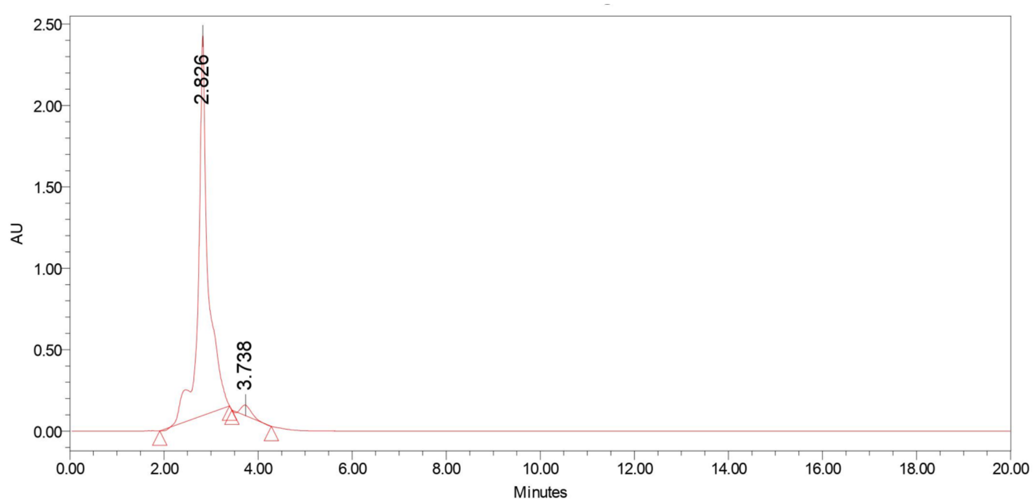

Peak Results

	Name	RT	Area	Height	Amount	Units	% Area
1		2.826	33925769	2330301			97.09
2		3.738	1015625	64452			2.91

Figure S45. UPLC of **3j**

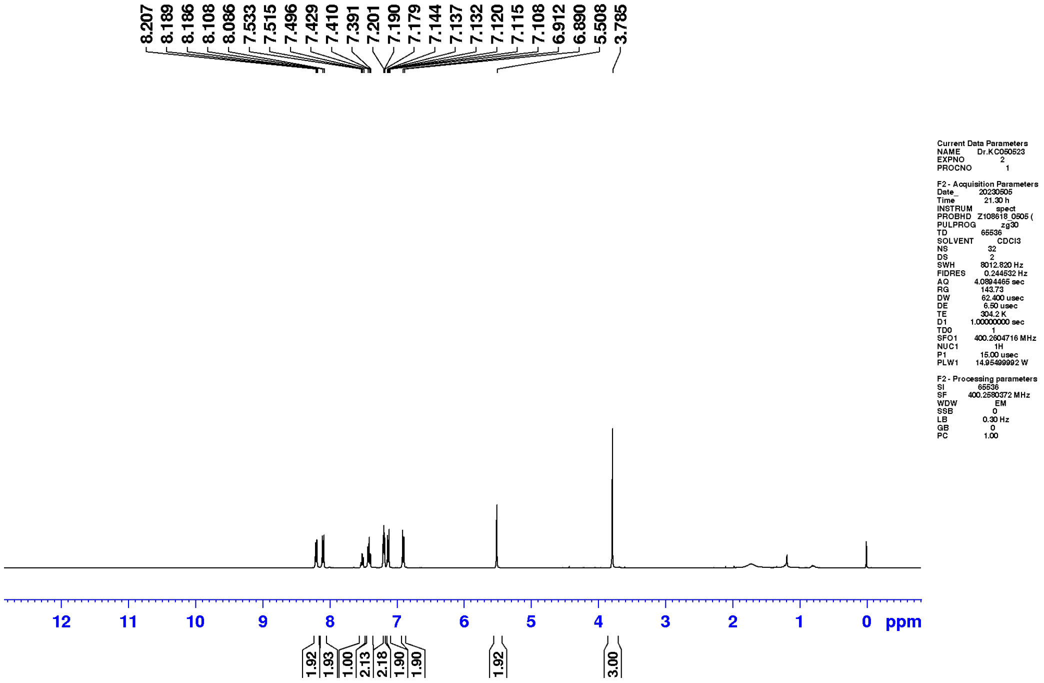

Figure S46. ^{1}H NMR of **3k**

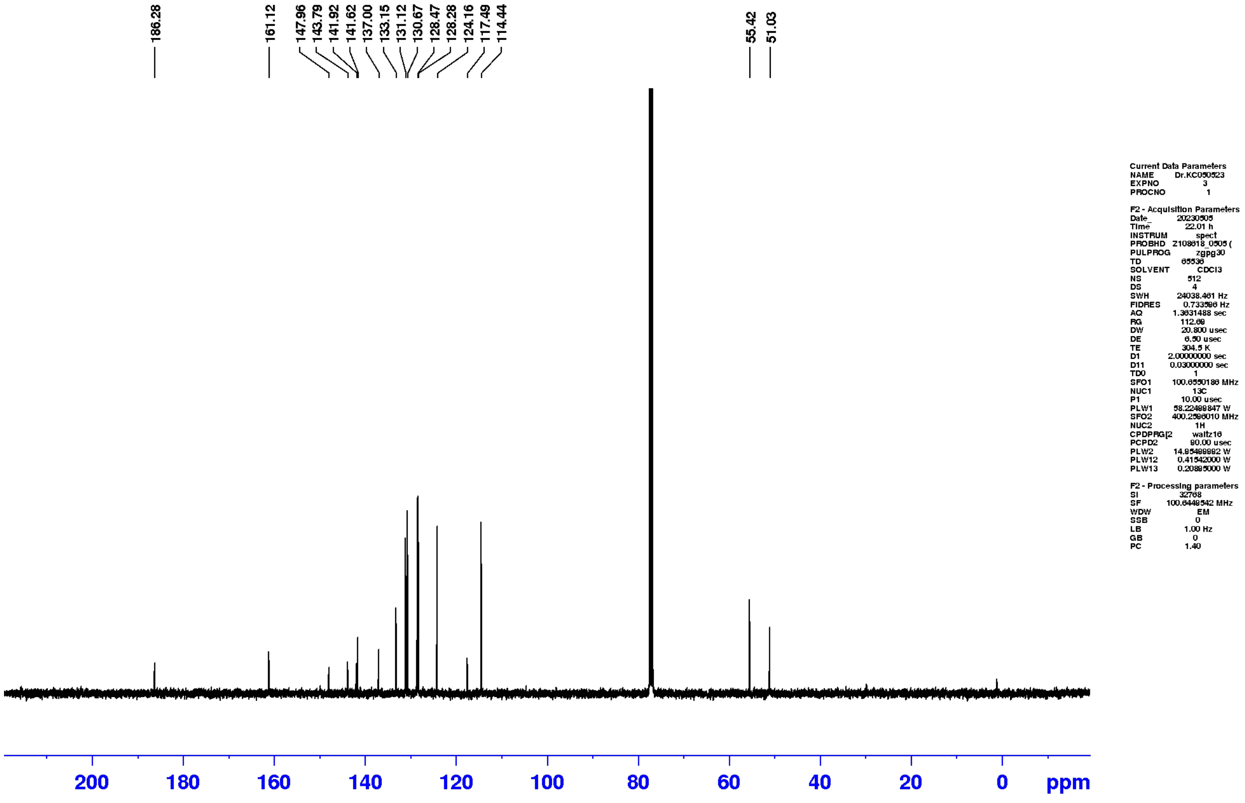

Figure S47. ^{13}H NMR of **3k**

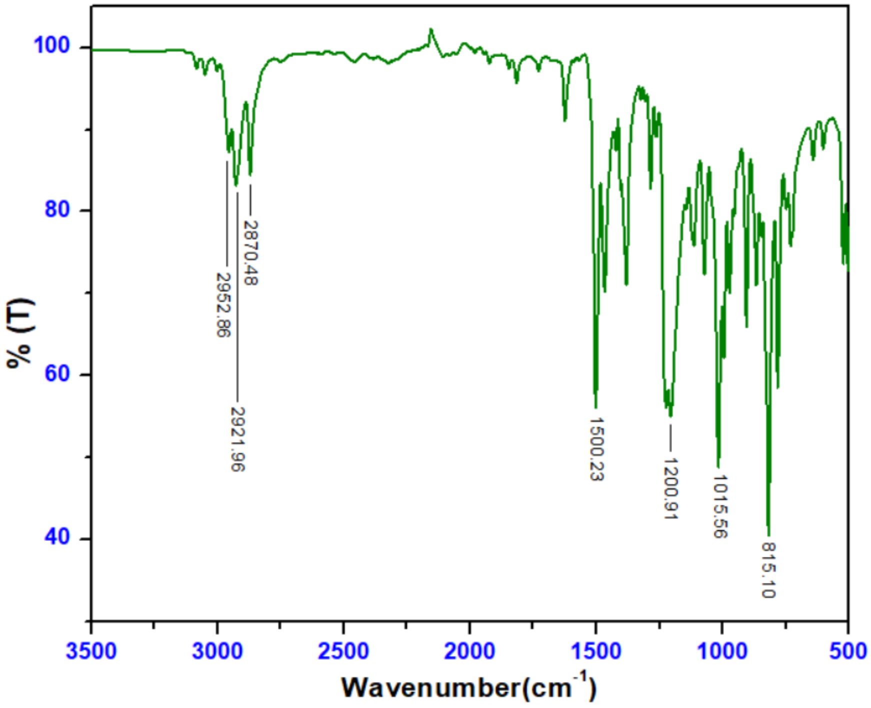

Figure S48. IR spectrum of **3k**

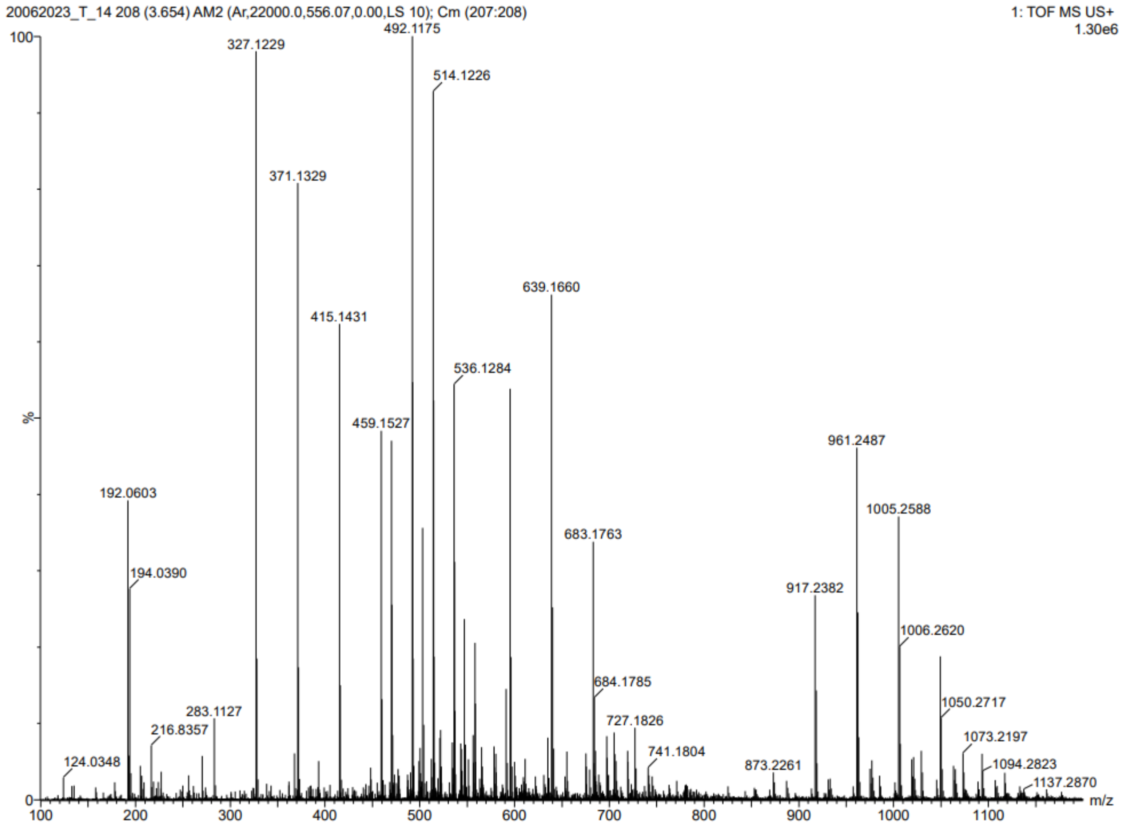

Figure S49. ESI HRMS of **3k**

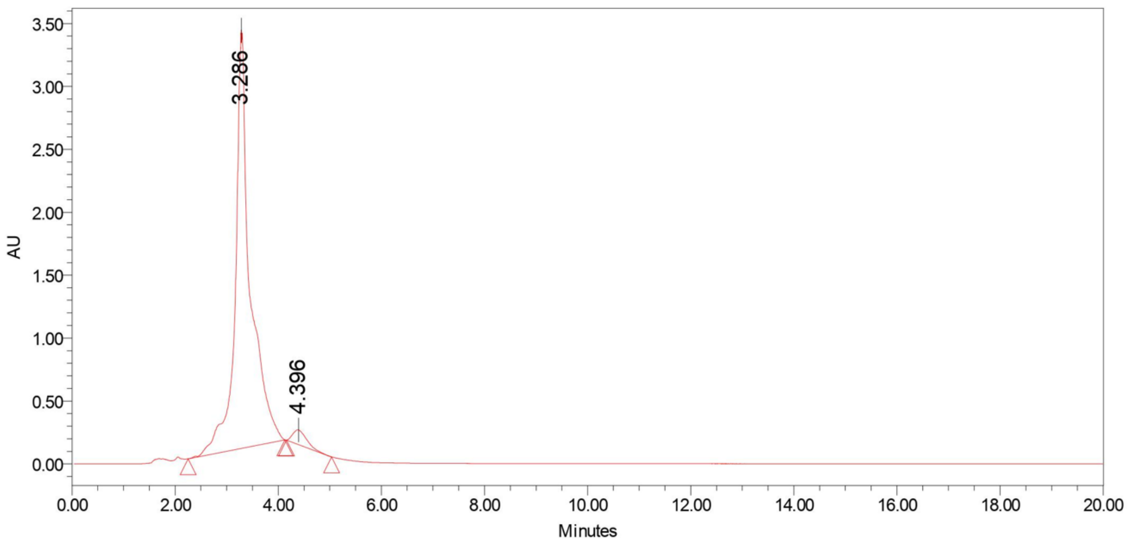

Peak Results

	Name	RT	Area	Height	Amount	Units	% Area
1		3.286	66760912	3320991			96.85
2		4.396	2171570	116126			3.15

Figure S50. UPLC of **3k**

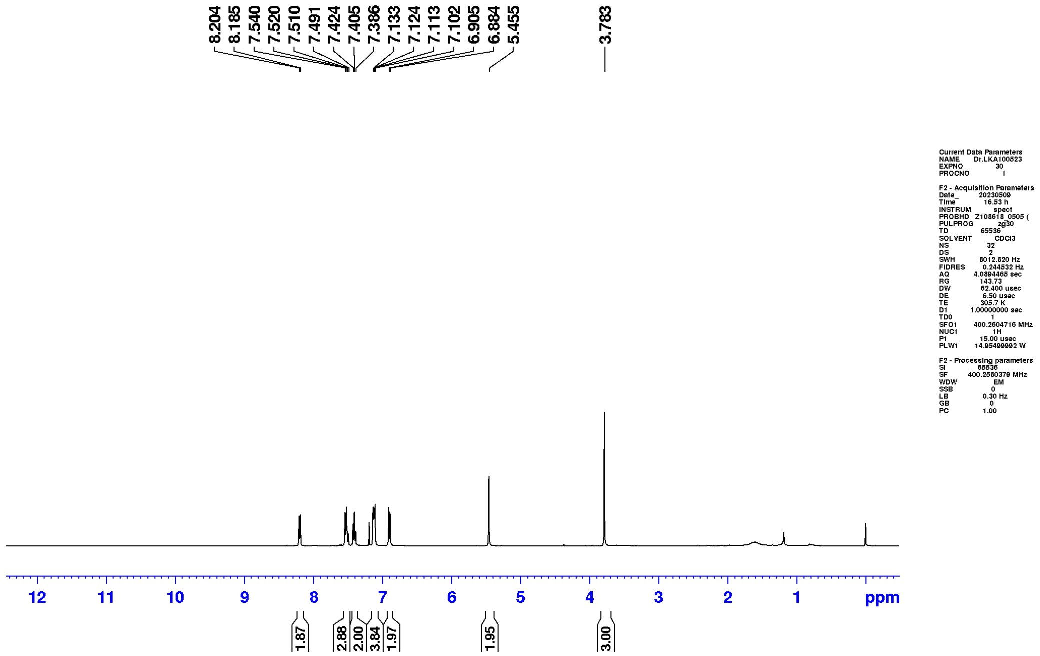

Figure S51. ^{1}H NMR of **3l**

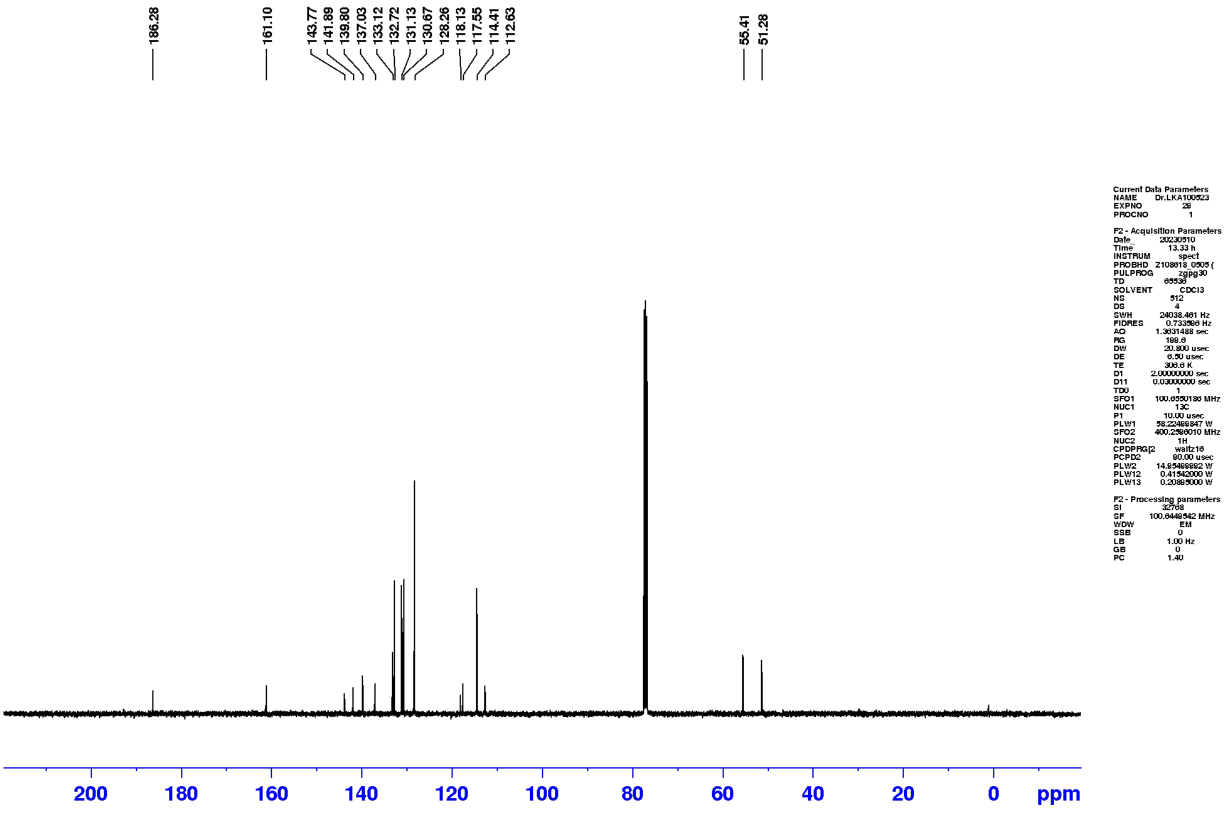

Figure S52. ^{13}H NMR of **3l**

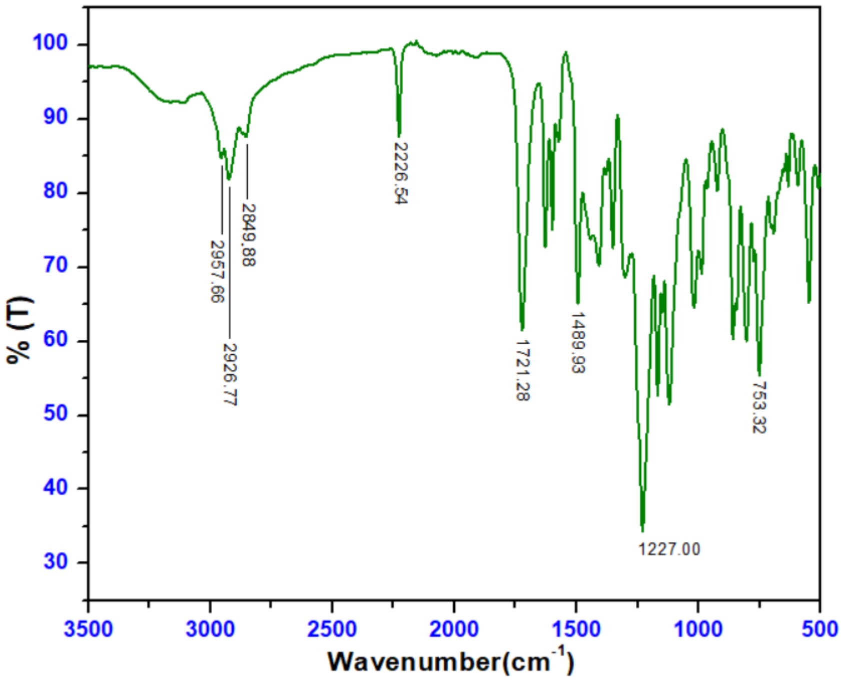

Figure S53. IR spectrum of **3l**

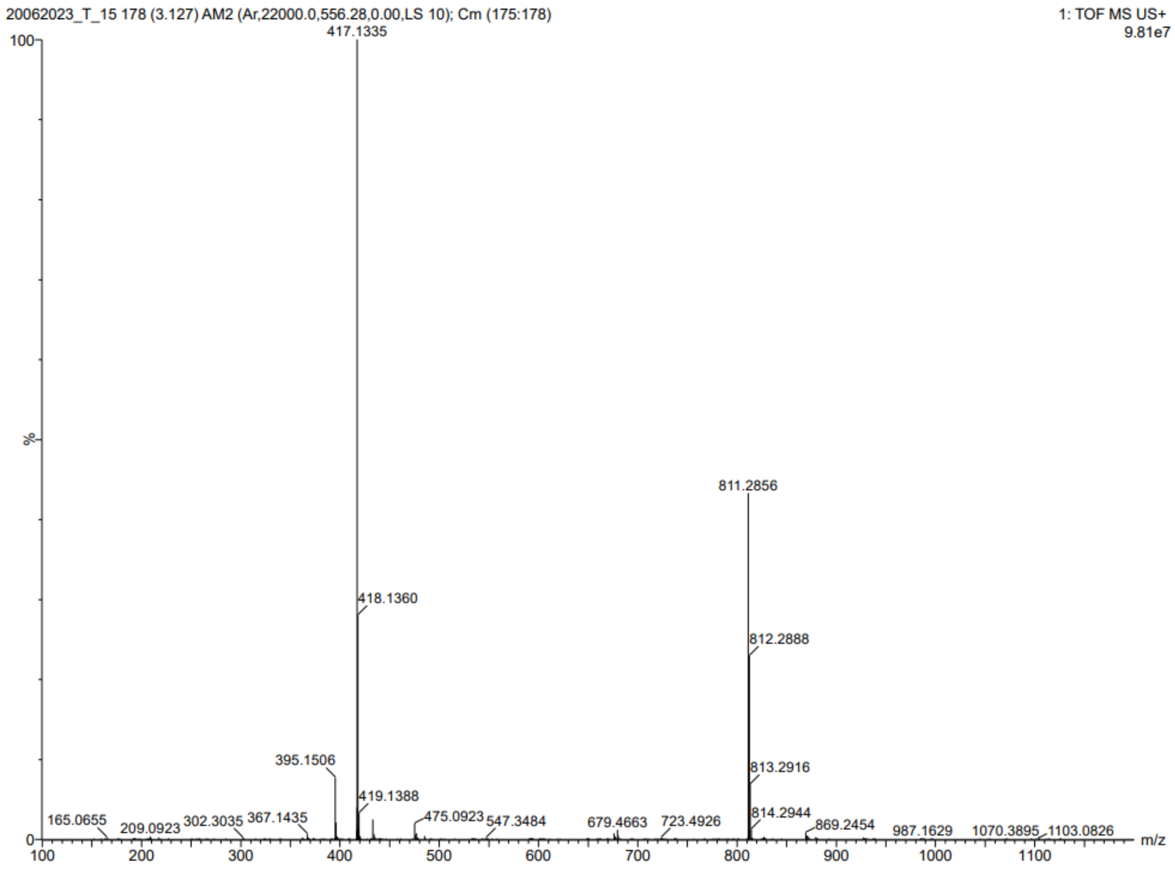

Figure S54. ESI HRMS of **3l**

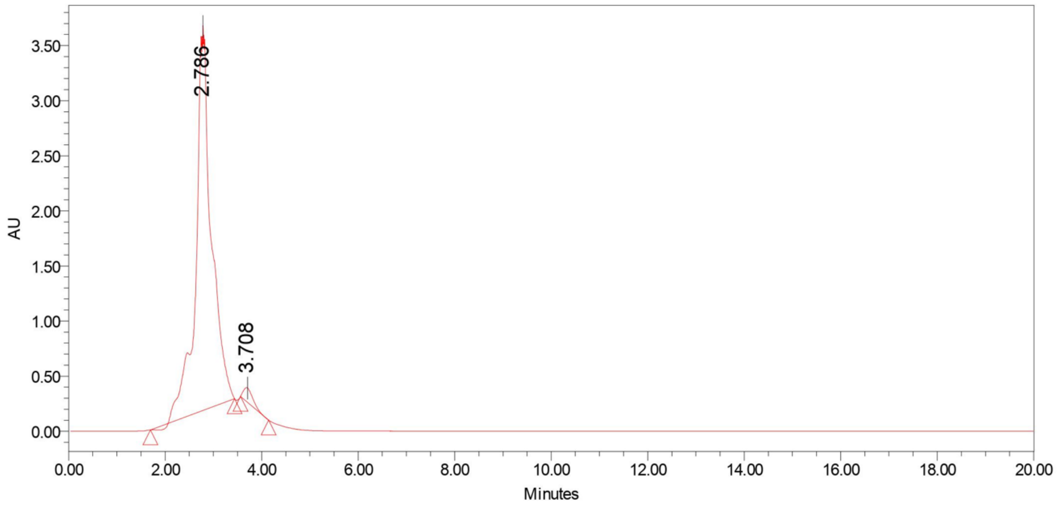

Peak Results

	Name	RT	Area	Height	Amount	Units	% Area
1		2.786	78634417	3489357			97.83
2		3.708	1745595	130357			2.17

Figure S55. UPLC of **3l**

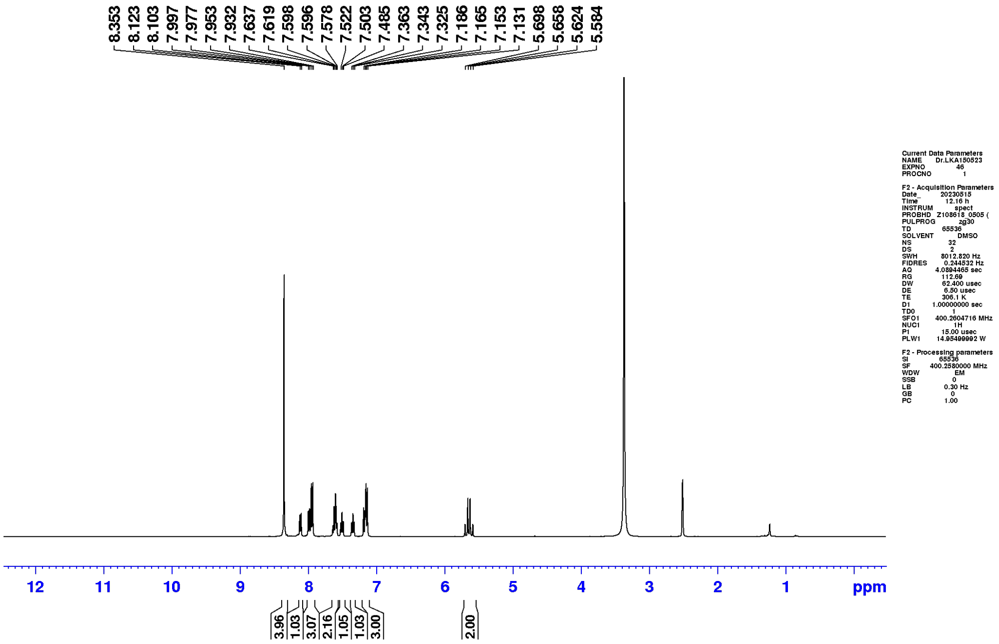

Figure S56. ^{1}H NMR of **3m**

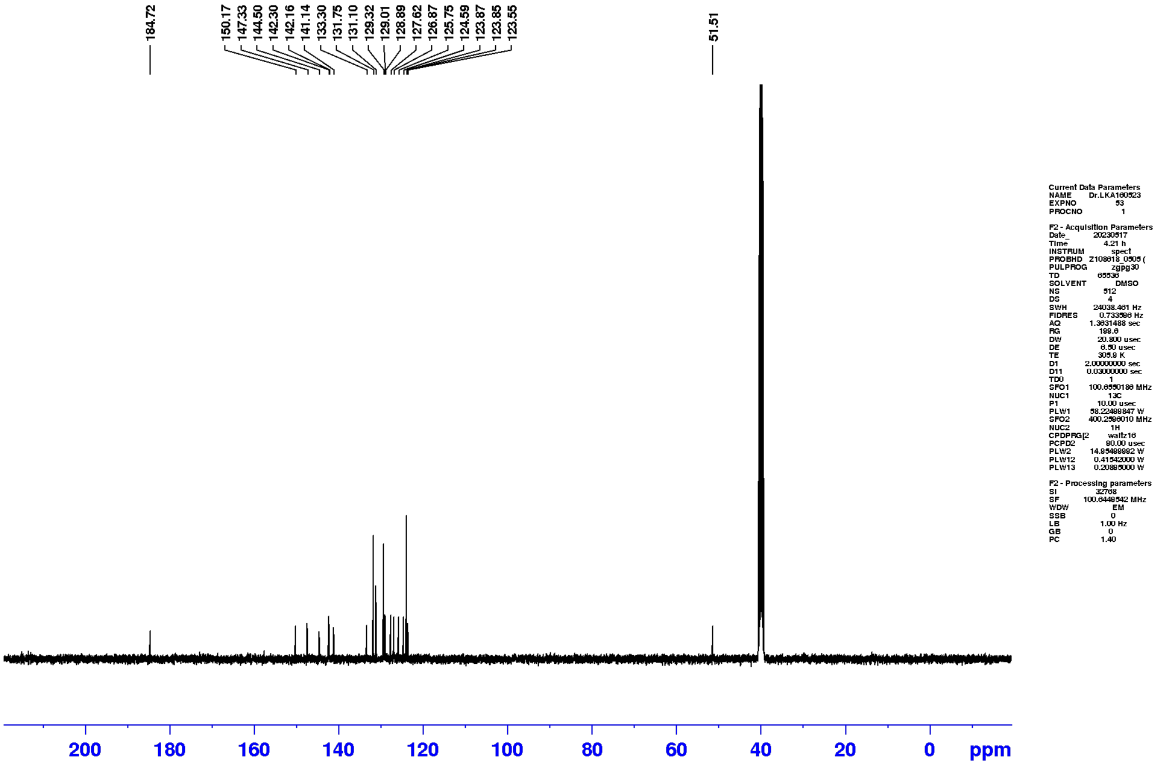

Figure S57. ^{13}H NMR of **3m**

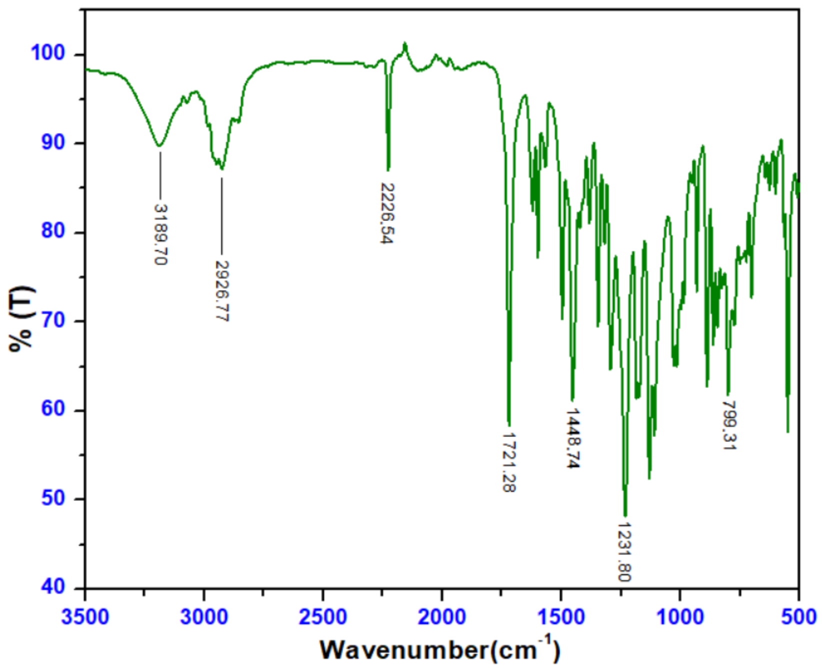

Figure S58. IR spectrum of **3m**

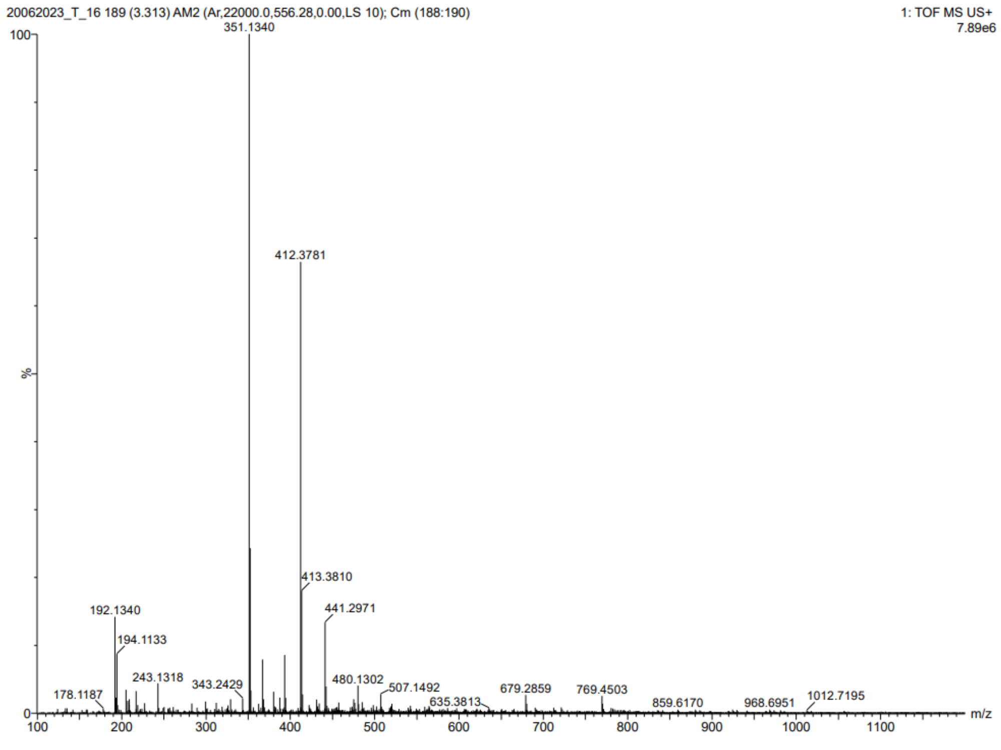

Figure S59. ESI HRMS of **3m**

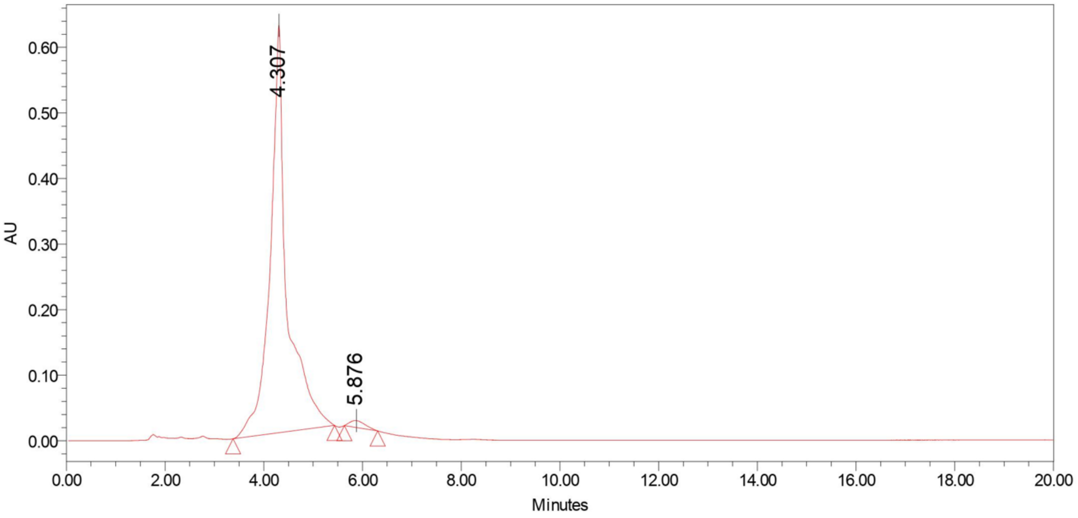

	Name	RT	Area	Height	Amount	Units	% Area
1		4.307	14679444	621499			98.47
2		5.876	228630	10517			1.53

Figure S60. UPLC of **3m**

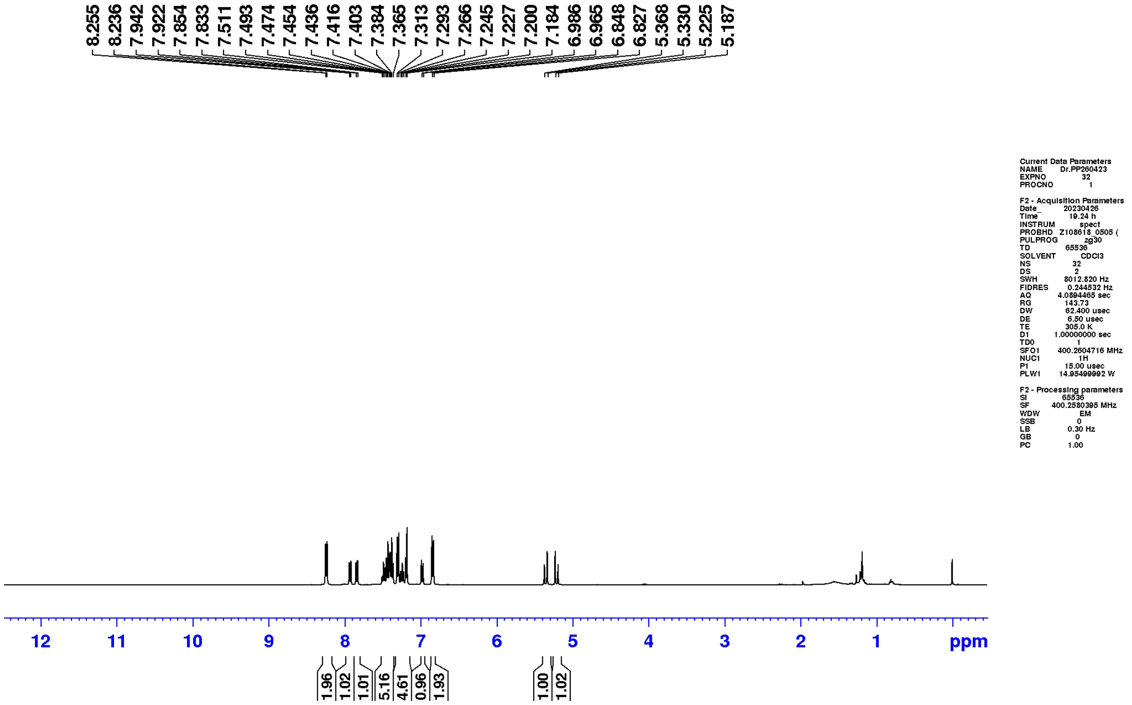

Figure S61. ^{1}H NMR of **3n**

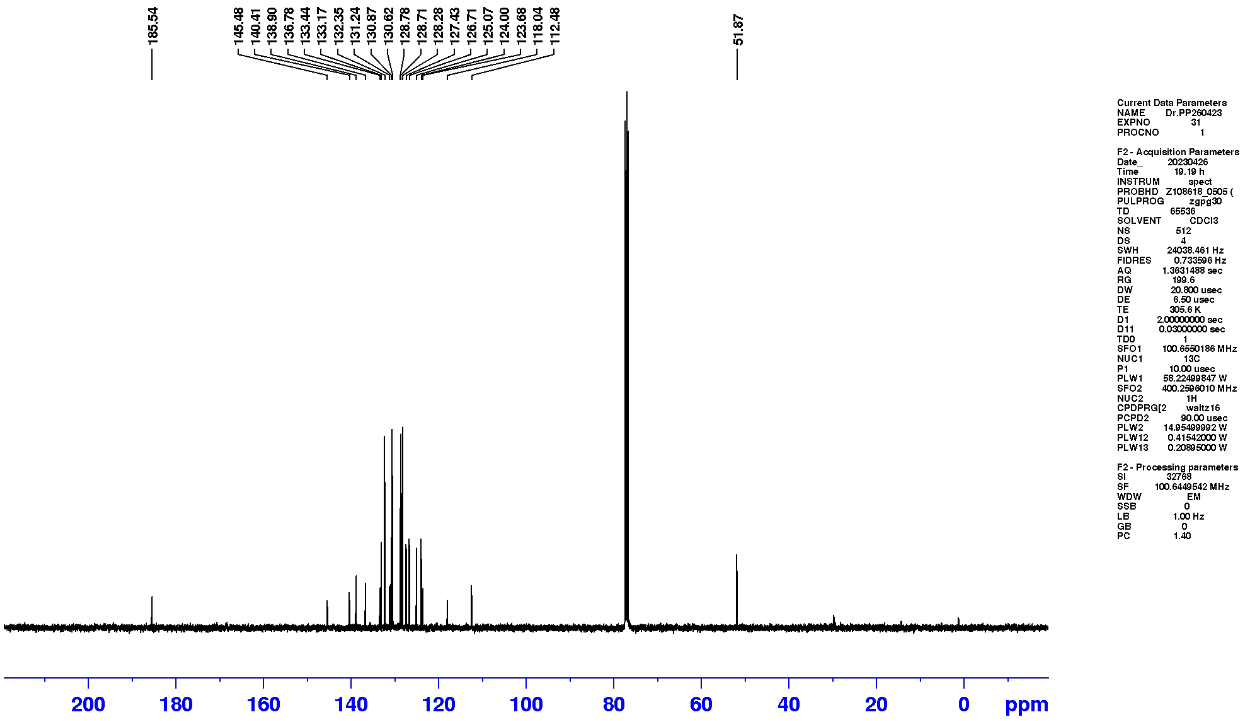

Figure S62. ^{13}H NMR of **3n**

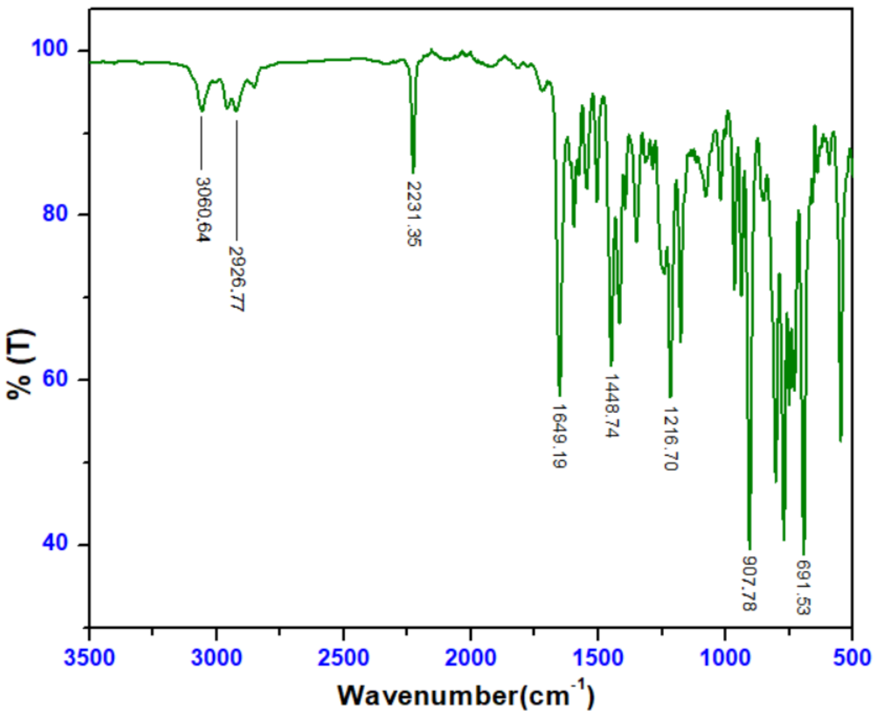

Figure S63. IR spectrum of **3n**

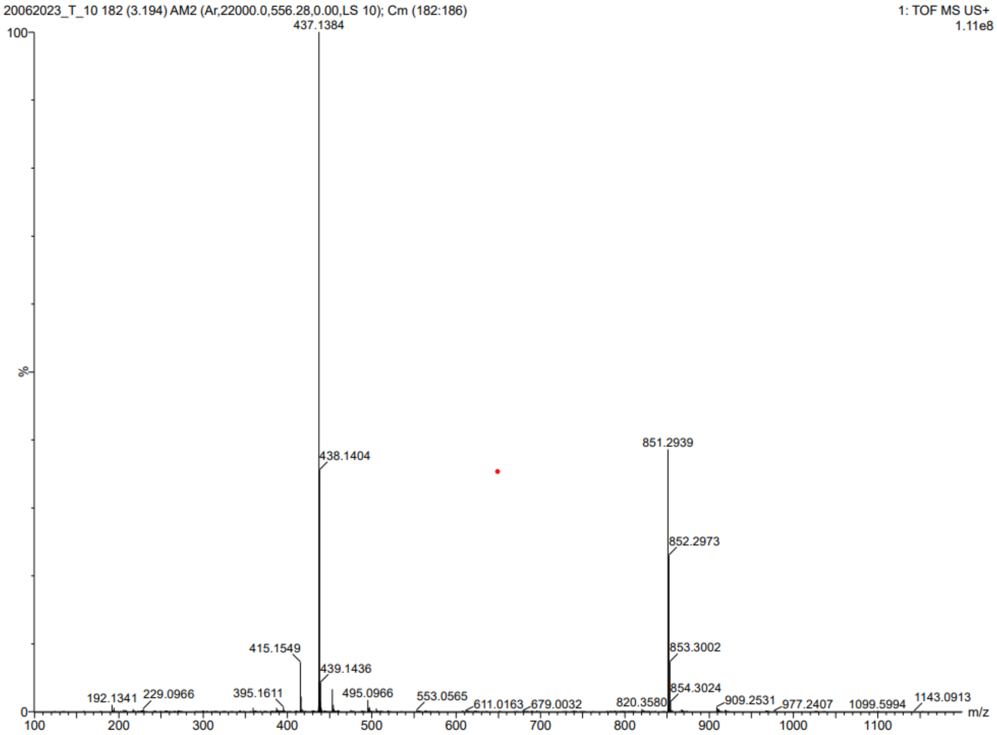

Figure S64. ESI HRMS of **3n**

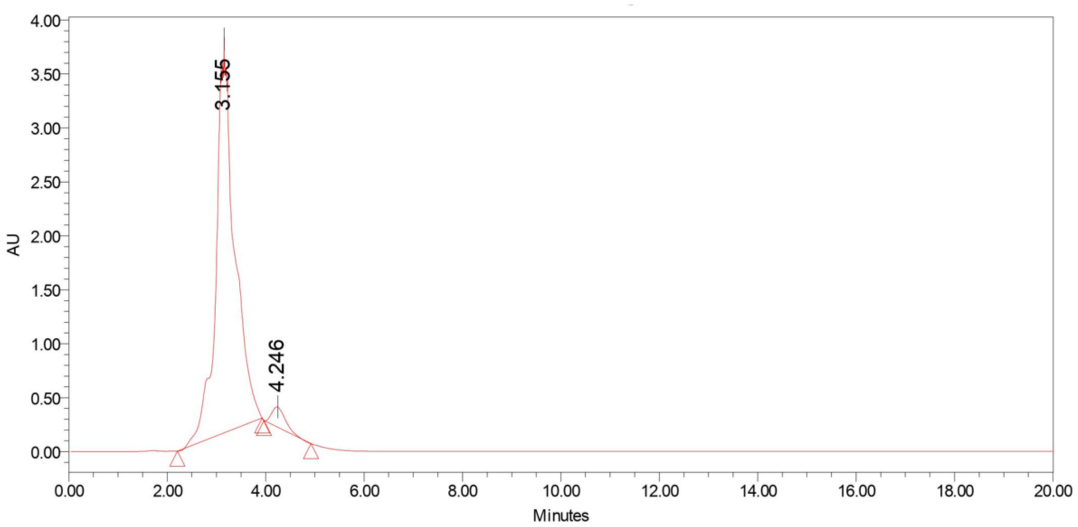

Peak Results

	Name	RT	Area	Height	Amount	Units	% Area
1		3.155	94479740	3669559			96.32
2		4.246	3612163	187229			3.68

Figure S65. UPLC of **3n**

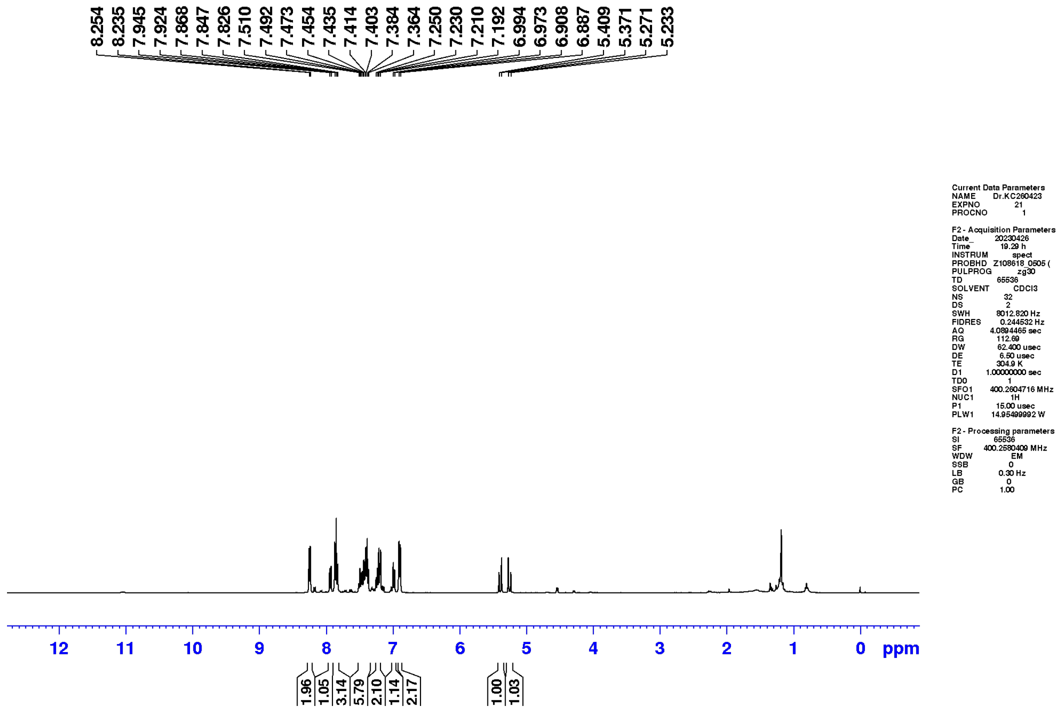

Figure S66. ^{1}H NMR of **3o**

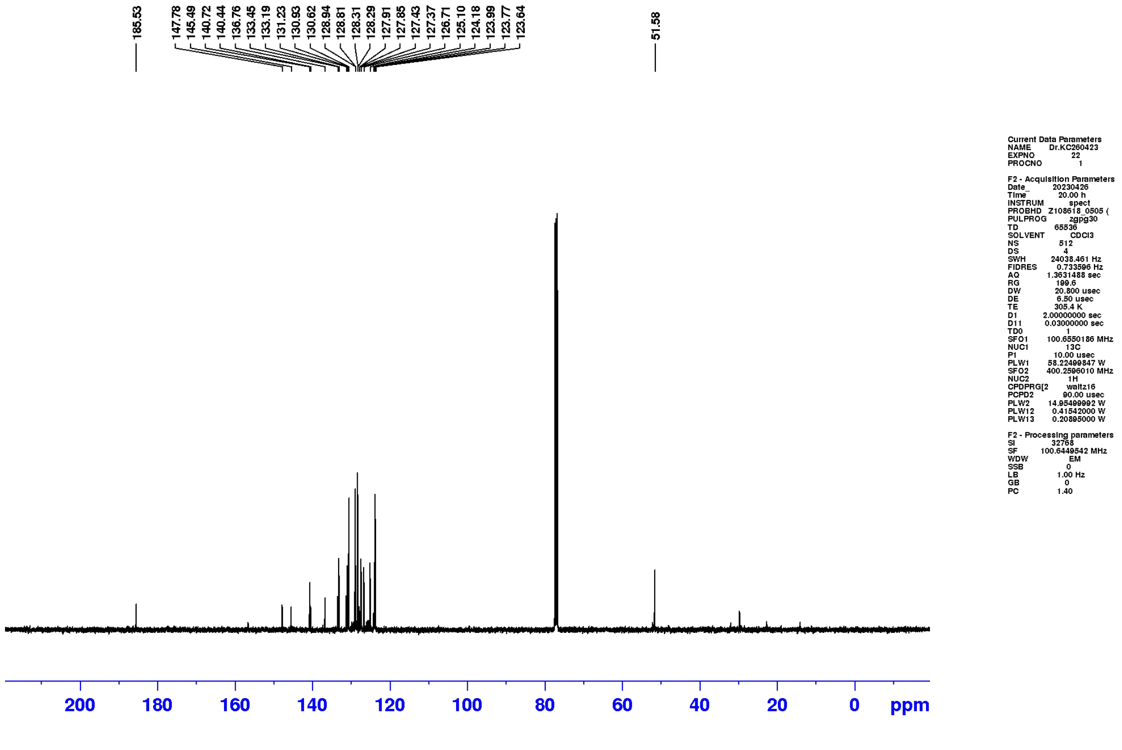

Figure S67. ^{13}C NMR of **3o**

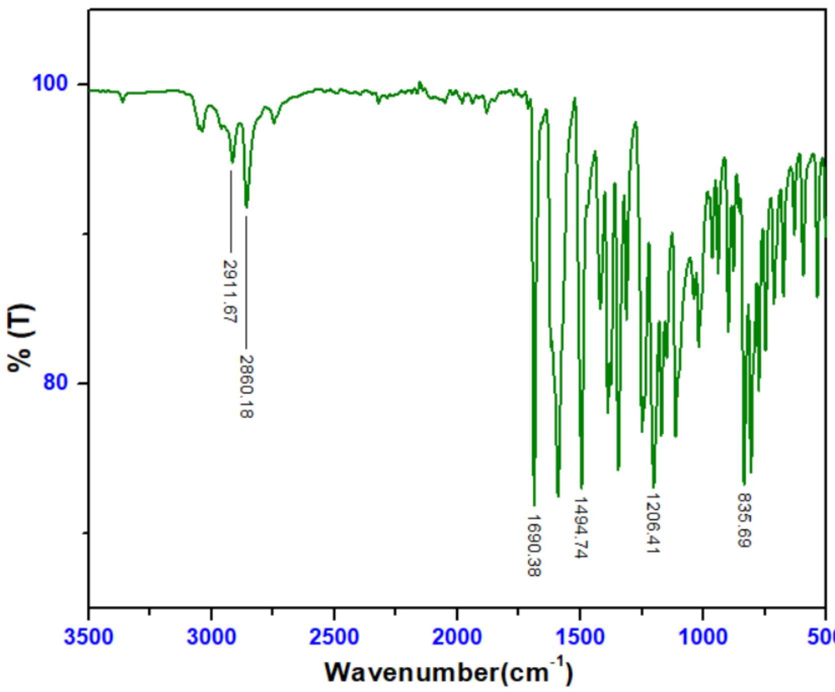

Figure S68. IR spectrum of **3o**

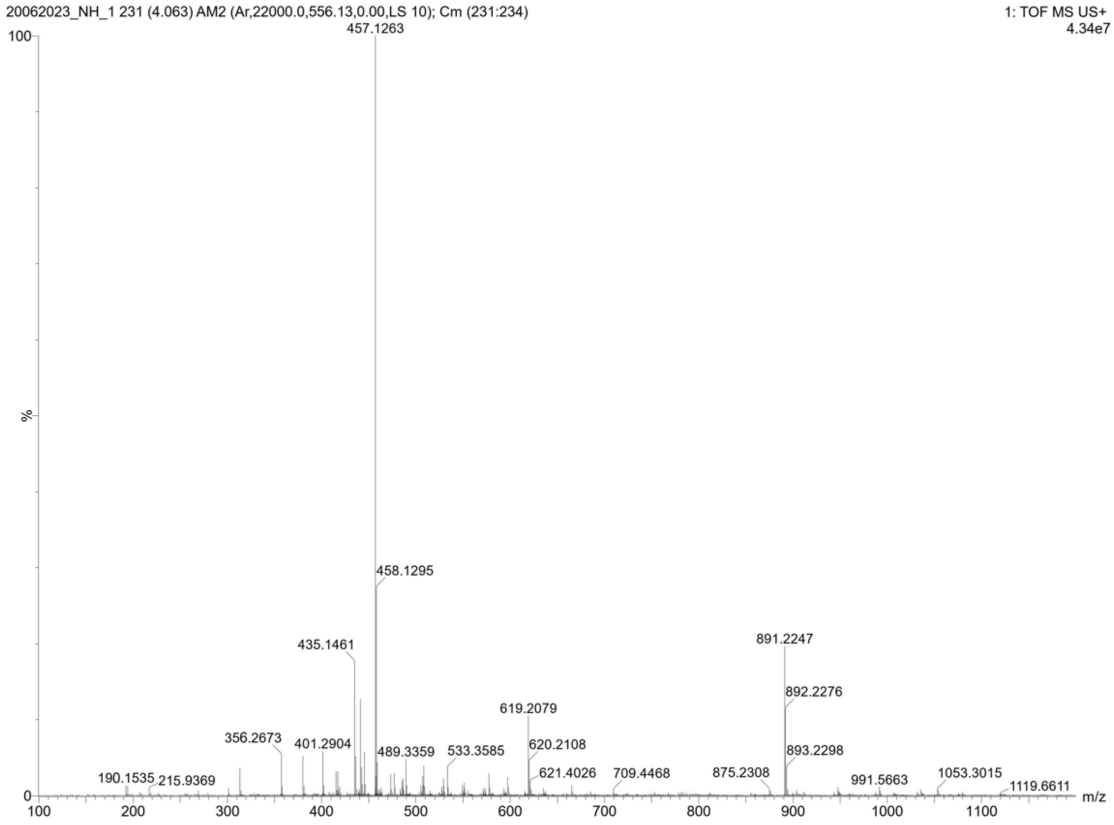

Figure S69. ESI mass of **3o**

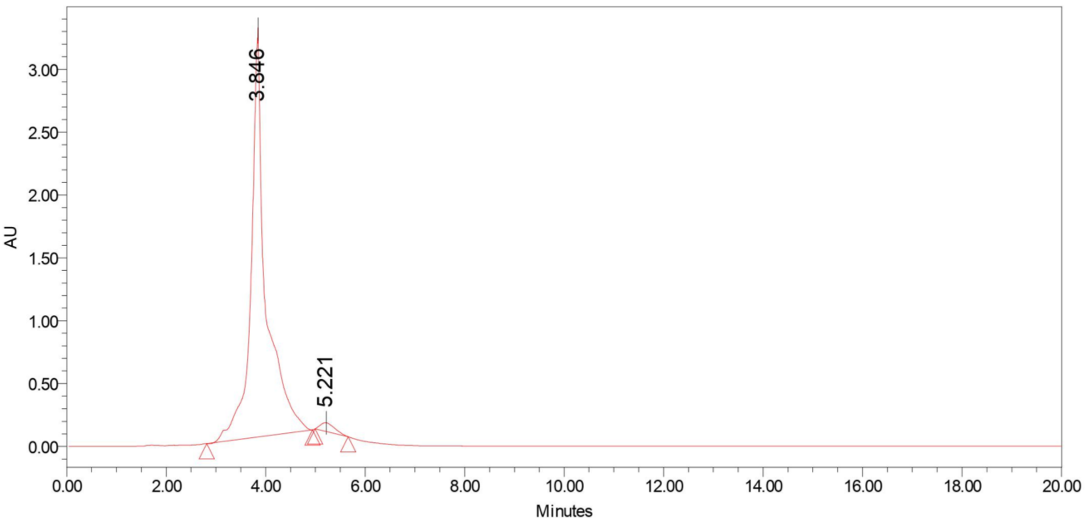

Peak Results

	Name	RT	Area	Height	Amount	Units	% Area
1		3.846	66780101	3251175			97.96
2		5.221	1392183	68818			2.04

Figure S70. UPLC of **3o**
